# Direct propylene epoxidation with molecular oxygen over titanosilicate zeolites

**DOI:** 10.1093/nsr/nwae305

**Published:** 2024-08-27

**Authors:** Weijie Li, Bin Qin, Zhuoya Dong, Yuchao Chai, Guangjun Wu, Yanhang Ma, Meng Wang, Xingwu Liu, Ding Ma, Landong Li

**Affiliations:** Key Laboratory of Advanced Energy Materials Chemistry of Ministry of Education, College of Chemistry, Nankai University, Tianjin 300071, China; Key Laboratory of Advanced Energy Materials Chemistry of Ministry of Education, College of Chemistry, Nankai University, Tianjin 300071, China; School of Physical Science and Technology, ShanghaiTech University, Shanghai 201210, China; Key Laboratory of Advanced Energy Materials Chemistry of Ministry of Education, College of Chemistry, Nankai University, Tianjin 300071, China; Key Laboratory of Advanced Energy Materials Chemistry of Ministry of Education, College of Chemistry, Nankai University, Tianjin 300071, China; School of Physical Science and Technology, ShanghaiTech University, Shanghai 201210, China; Beijing National Laboratory for Molecular Sciences, New Cornerstone Science Laboratory, College of Chemistry and Molecular Engineering, Peking University, Beijing 100871, China; Beijing National Laboratory for Molecular Sciences, New Cornerstone Science Laboratory, College of Chemistry and Molecular Engineering, Peking University, Beijing 100871, China; Beijing National Laboratory for Molecular Sciences, New Cornerstone Science Laboratory, College of Chemistry and Molecular Engineering, Peking University, Beijing 100871, China; Key Laboratory of Advanced Energy Materials Chemistry of Ministry of Education, College of Chemistry, Nankai University, Tianjin 300071, China; Frontiers Science Center for New Organic Matter, College of Chemistry, Nankai University, Tianjin 300071, China

**Keywords:** titanosilicate zeolites, propylene epoxidation, molecular oxygen

## Abstract

The direct epoxidation of propylene with molecular oxygen represents a desired route for propylene oxide (PO) production with 100% theoretical atomic economy. However, this aerobic epoxidation reaction suffers from the apparent trade-off between propylene conversion and PO selectivity, and remains a key challenge in catalysis. We report that Ti-Beta zeolites containing isolated framework Ti species can efficiently catalyze the aerobic epoxidation of propylene. Stable propylene conversion of 25% and PO selectivity of up to 90% are achieved at the same time, matching the levels of industrial ethylene aerobic epoxidation processes. H-terminated pentacoordinated Ti species in Beta zeolite frameworks are identified as the preferred active sites for propylene aerobic epoxidation and the reaction is initiated by the participation of lattice oxygen in Ti-OH. These results are expected to spark new technology for the industrial production of PO toward more sustainable chemistry and chemical engineering.

## Teaser

Titanosilicate zeolites containing isolated framework Ti species, namely Ti-Beta, can efficiently catalyze the direct epoxidation of propylene with molecular oxygen, triggering new process for the industrial production of propylene oxide.

## INTRODUCTION

**Scheme 1.**
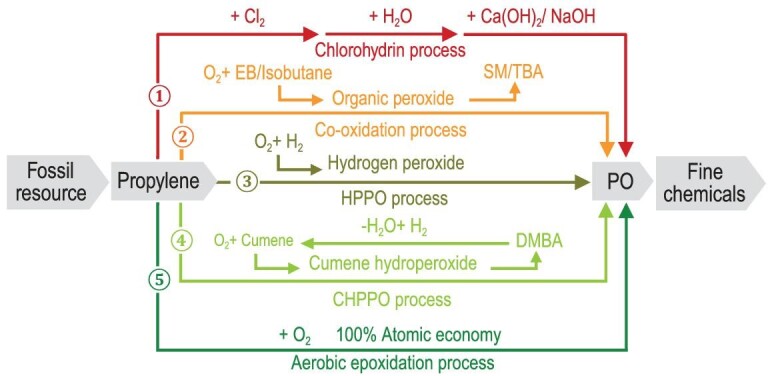
Evolution of processes for PO production from propylene. The past chlorohydrin process (1), the current co-oxidation (2), HPPO (3) and CHPPO (4) processes, as well as the future aerobic epoxidation process (5). PO: propylene oxide; EB: ethylbenzene; SM: styrene; TBA: tert-butanol; DMBA: dimethylbenzyl alcohol.

Propylene epoxide (PO) represents the third largest propylene derivative and PO production from propylene is a pivotal process in the chemical industry [1]. In the past decades, PO production processes have transformed from the chlorohydrination process to the co-oxidation, HPPO and CHPPO processes (Scheme 1), complying with the principle of green chemistry and sustainable industry [2,3]. However, all of the current processes are complicated, containing multi-staged liquid-phase reactions and suffer from low efficiency, making PO production expensive. Direct propylene epoxidation with molecular oxygen, namely aerobic epoxidation, is the ideal choice for PO production with 100% theoretical atomic economy (Scheme 1). Nowadays, the aerobic epoxidation of ethylene using supported Ag catalysts is widely employed in the industrial production of ethylene oxide (EO) [4]. For propylene, the allylic hydrogen is very sensitive to the reactive oxygen species and therefore makes controlling the selectivity toward target PO products difficult [5,6]. While several catalyst systems, namely Cu [7–10], Ag [11,12], Au [13–15], CuAu [16] and Co [17], have been explored for propylene aerobic epoxidation, it is impossible to achieve high PO selectivity at reasonable propylene conversions, not to mention catalytic stability. The reaction of propylene aerobic epoxidation is recognized as the Holy Grail in catalysis research [18], with an apparent trade-off between propylene conversion and PO selectivity.

Herein, we report the efficient aerobic epoxidation of propylene using titanosilicate zeolites containing isolated framework Ti species, namely Ti-Beta, as catalysts. Stable propylene conversion of ∼17% and PO selectivity of 85%–90% can be achieved at the same time to match the levels of industrial ethylene aerobic epoxidation processes. Such catalytic results are meaningful for the industrial production of PO and the unique reaction mechanism will pave the way for various selective catalytic oxidations.

## RESULTS AND DISCUSSION

The Ti-Beta samples are prepared by a simple solid-phase metalation route, i.e. replenishing the vacancies of Si-Beta with a TiCp_2_Cl_2_ precursor and subsequently stabilizing isolated Ti centers *via* the formation of Ti–O–Si linkages [19]. The process can be monitored by X-ray diffraction (XRD), Fourier-transform infrared spectroscopy (FTIR) and solid-state nuclear magnetic resonance (NMR) (Figs S1–S5). As a result, 1%–4% Ti atoms can be incorporated into a Beta zeolite framework with the textural structure of the zeolite being well preserved (Fig. S6 and Table S1). The existence of Ti*_x_*O*_y_* clusters or bulk TiO_2_ can be excluded by Raman spectroscopy (Fig. S7). Scanning transmission electron microscopy (STEM) implies the homogeneous distribution of Ti species within Ti-Beta samples (Figs S8, S9).

**Figure 1.**
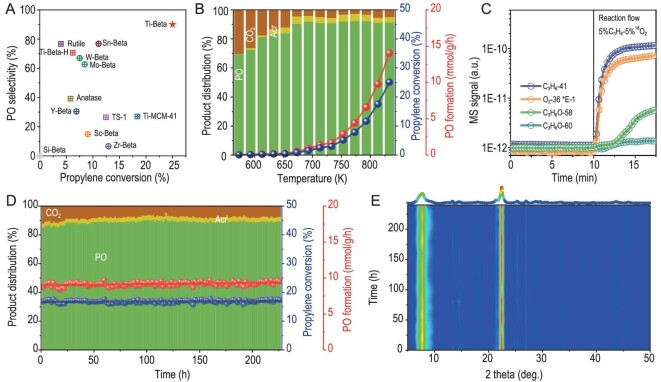
Catalytic behaviors of Ti-Beta in propylene aerobic oxidation. (A) Comparison of various catalysts for propylene aerobic oxidation in terms of propylene conversion and PO selectivity. Reaction conditions: 0.1 g catalyst, 5%C_3_H_6_-5%O_2_-90%He, GHSV = 36 000 mL/g/h. (B) Temperature-dependent behaviors of propylene aerobic oxidation over Ti-Beta-3% catalyst. Reaction conditions: 0.1 g catalyst, 5%C_3_H_6_-5%O_2_-90%He, GHSV = 36 000 mL/g/h. (C) Transient kinetic analysis results of Ti-Beta catalyst. After activation and He purging of 0.1 g Ti-Beta catalyst, the stream was switched from 60 mL/min He to 60 mL/min 5%C_3_H_6_-5%^18^O_2_-90%He, at 10 min at 773 K. (D) Stability test of Ti-Beta-3% catalyst in propylene aerobic oxidation at 813 K. Reaction conditions: 0.1 g catalyst, 5%C_3_H_6_-5%O_2_-90%He, GHSV = 36 000 mL/g/h. (E) *In situ* XRD patterns of Ti-Beta-3% during propylene aerobic epoxidation at 813 K.

Titanosilicates are known as efficient catalysts for olefin epoxidation with H_2_O_2_ [19–21] and TS-1 is currently employed in catalyzing propylene epoxidation in the HPPO process [22]. In this work, titanosilicates show considerable catalytic activity in the gas-phase direct propylene epoxidation with molecular oxygen at high reaction temperatures, with Ti-Beta prepared *via* post-synthesis modification appearing to be a better catalyst than Ti-Beta-H prepared *via* the direct hydrothermal route, TS-1 and Ti-MCM-41 (details in Supplementary Materials, Fig. S10). The presence of residual Al, i.e. the Brønsted acid sites, in Ti-Beta zeolite catalyst shows significant negative impact on the reaction and should be avoided (Fig. S11). Since Si-Beta is completely inactive for the reaction, the catalytic activity of Ti-Beta comes exclusively from Ti species. The catalytic activity of Ti-Beta is highly dependent on the Ti content and Ti-Beta-3% can be optimized for the reaction (Fig. S12). Ti-Beta is a typical solid Lewis acid [23], and therefore other Lewis acidic M-Beta zeolites are also investigated for the same reaction (Fig. S13). Sn-Beta, Mo-Beta and W-Beta catalysts are active and selective for PO formation; however they are distinctly inferior to Ti-Beta (Fig. 1A). The reaction parameters like gas-hourly space velocity (GHSV) and C_3_H_6_/O_2_ ratio show noticeable impacts on the catalytic performance (Figs S14, S15), while the intrinsic catalytic properties of Ti-Beta-3% will not be altered. The temperature-dependent behaviors of Ti-Beta-3% disclose that the propylene conversion increases with reaction temperature from 673 to 833 K while the PO selectivity is surprisingly high (80%–90%, Fig. 1B). Experimentally, ∼90% PO selectivity can be obtained at 25% propylene conversion under optimized reaction conditions, offering a state-of-the-art PO formation rate of 14.0 mmol/g/h. It is a key breakthrough in propylene aerobic oxidation, which is meaningful for industrial application, in analogy to the mature process of ethylene aerobic oxidation (Figs S16, S17, Table S2, and additional discussion in Supplementary data). A direct comparison with representative literature results on propylene aerobic epoxidation is summarized in Fig. S18 and Table S3. Furthermore, a primary comparison between chlorohydrin, HPPO and aerobic epoxidation processes is shown in Table 1, demonstrating the potential of aerobic epoxidation processes for industrial application.

**Table 1.** Comparison of PO production from chlorohydrin, HPPO and aerobic epoxidation processes.

Production process	Chlorohydrin	HPPO	Aerobic epoxidation
Atomic efficiency	31.2%	76.3%	100%
Reaction type	Liquid-phase	Liquid-phase	Gas-phase
Reaction temperature	313–363 K	303–333 K	793–833 K
Catalyst	/	TS-1	Ti-Beta
Substrate conversion	C_3_H_6_: 90%–95%	H_2_O_2_: 95%–99% C_3_H_6_: 10%–50%	C_3_H_6_: 11%–25%
Propylene recirculation	No	Yes	Yes
PO selectivity	/	90%–98%	85%–90%
PO space-time-yield	/	31–32 mmol/g_cat_/h	∼14 mmol/g_cat_/h
Cost of propylene*	$856 per ton PO	$824 per ton PO	$888 per ton PO
Cost of O_2_*	/	/	$50 per ton PO
Cost of Cl_2_*	$20 per ton PO	/	/
Cost of H_2_O_2_*	/	$236 per ton PO	/
Cost of methanol*	/	$12 per ton PO	/
Cost of Ca(OH)_2_	$123 per ton PO	/	/
Total cost	$999 per ton PO	$1072 per ton PO	$938 per ton PO
Solvent or additive	Ca(OH)_2_	Methanol	None
Waste water	Yes	Yes	No
Profit of PO	$161 per ton PO	$88 per ton PO	$222 per ton PO

* Obtained from *Global Market Outlook-Chemicals* (2022) published by the database of Independent Commodity Intelligence Services (ICIS). **Note**: The chlorohydrin and HPPO processes are quite mature through decades of protracted and unremitting efforts, while the aerobic epoxidation process is still in the nascent stage, showing potential for practical applications. For the scale-up of propylene aerobic epoxidation process, many important issues, for example the reaction system, product separation and heat management, should be affirmed and optimized. Herein, only a primary comparison between these routes of PO production is shown.

Temperature-programmed surface reaction (TPSR) profiles of C_3_H_6_-O_2_ confirm the formation of PO as the major product in the aerobic oxidation of propylene at >573 K while C_3_H_6_-TPSR profiles show that PO can be formed even in the absence of molecular oxygen (Fig. S19). Temperature-programmed desorption experiments rule out the adsorption of propylene and molecular oxygen at temperatures higher than 498 K (Fig. S20). This indicates that propylene aerobic oxidation occurs with the participation of lattice oxygen, probably *via* the Mars-van-Krevelen mechanism [24]. Indeed, when feeding propylene to the ^18^O-enriched Ti-Beta catalyst at a constant temperature of 773 K, the formation of ^18^O-labeled PO as the primary product was observed (Fig. S21). Feeding C_3_H_6_-^18^O_2_ to Ti-Beta at 673 (Fig. S22) or 773 K (Fig. 1C) leads to the formation of both ^16^O-PO (*m*/*z* = 58) and ^18^O-PO (*m*/*z* = 60) in transient kinetic analysis while the ^18^O-PO signal is much weaker and lags behind the ^16^O-PO signal, confirming the dominant pathway of PO formation from lattice oxygen and its pivotal role in the reaction process. As a result, stable intrinsic selectivity toward PO at high PO formation rates from 713 to 833 K was obtained (Fig. S23). The reaction order of C_3_H_6_ is measured as ∼0.4 (0.33–0.46) for both PO and CO*_x_* (mostly CO_2_, trace CO) formation, and the reaction order of O_2_ is measured as 0.17–0.38 and 0.62–0.97 for PO and CO*_x_* formation at 793 to 833 K (Figs S24–S26), respectively. It implies that the main and side reactions might include the same elementary step for propylene participation while the CO*_x_* formation is more sensitive to the partial pressure of molecular oxygen [25,26]. TPSR profiles of PO and acrolein in the presence of molecular oxygen confirm that the CO_2_ byproduct comes from the further oxidation of acrolein over the Ti-Beta-3% catalyst (Fig. S27). These results clearly demonstrate the unique catalytic behaviors of Ti-Beta-3% with the participation of lattice oxygen, which are beneficial to catalytic stability. Indeed, Ti-Beta-3% shows perfect stability at a high reaction temperature of 813 K with no activity loss being observed for over 240 h (propylene conversion of ∼17% and PO selectivity of ∼88%, Fig. 1D, Fig. S28). The formation of coke deposit can be ruled out by thermogravimetric analysis (Fig. S29), and the intact structure characteristics of Ti-Beta are verified by *in situ* XRD (Fig. 1E), Ar sorption (Fig. S30) and UV-vis spectroscopy (Fig. S31).

**Figure 2.**
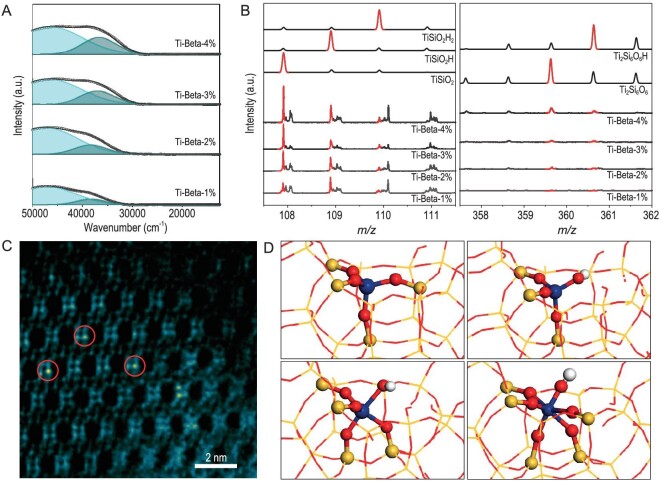
Characterization of Ti-Beta zeolite samples. (A) UV-vis spectra of Ti-Beta zeolites. (B) TOF-SIMS analyses of mononuclear Ti species in Ti-Beta zeolites. (C) *Cs*-corrected ADF-STEM image of Ti-Beta-3% taken along [100] direction. Sites with stronger contrast in the four-membered rings of Beta zeolite are highlighted by red circles. (D) Structure models for tetracoordinated Ti, H-terminated tetracoordinated Ti, H-terminated pentacoordinated and H-terminated hexacoordinated Ti species from DFT simulations. Ti: dark blue, O: red, Si: yellow, H: white.

The optical spectra of Ti-Beta samples show two distinct absorption bands at 48 000 and 37 000 cm^−1^ (Fig. 2A), assignable to ligand-to-metal charge transfer of framework tetrahedra Ti centers and Ti centers in higher coordination states like penta- or hexa-coordinated ones [27,28], respectively. In contrast, Ti-containing zeolites prepared *via* direct hydrothermal synthesis, namely TS-1 and Ti-Beta-H, show the dominant absorption band at 48 000 cm^−1^ corresponding to tetrahedra Ti centers (Fig. S32). The intensities of both tetrahedra Ti centers and Ti centers in higher coordination states in Ti-Beta samples increase with increasing Ti content from 1% to 4%, with no oligomeric or bulk Ti*_x_*O*_y_* species being observed with absorption bands below 30 000 cm^−1^ (Fig. S33). Meanwhile, the presence of H-terminated defective Ti species is characterized by Ti-OH signals as shown in FTIR (Fig. S3) and ^1^H NMR (Fig. S4) spectra. Mononuclear Ti species in Ti-Beta are characterized by the TiSiO_2_, TiSiO_2_H and TiSiO_2_H_2_ fragments from time-of-flight secondary ion mass spectrometry (TOF-SIMS, Fig. 2B). Ti_2_Si_6_O_6_ and Ti_2_Si_6_O_6_H fragments are also observed at high Ti content of 4%, corresponding to the six-membered rings of Beta zeolite with double Ti substitutions [29]. However, the fragments associated with dinuclear Ti species, namely Ti_2_O, Ti_2_OH, Ti_2_O_4_ and Ti_2_O_4_H, are not observed for Ti-Beta samples, in significant contrast to anatase/rutile TiO_2_ or TS-1 [21] (Figs S34, S35). These results clearly reveal the presence of dominant mononuclear Ti species in Ti-Beta-3%. TEM analyses (see details in Supplementary Materials, Fig. S36) show the obviously higher contrast at certain framework sites of Ti-Beta samples, which might be attributed to atomically dispersed Ti species (Fig. S37). The presence of a substantial amount of very small TiO*_x_* clusters can be resolved by UV-Vis (Fig. 2A) and TOF-SIMS (Fig. 2B) analysis. In the typical *Cs*-corrected ADF-STEM image of Ti-Beta-3%, isolated sites with stronger contrast in the four-membered rings are identified (Fig. 2C), corresponding to T1 to T6 crystallographic sites in framework of polymorph Beta_A (Fig. S38). The T6 site should be the most stable one according to density functional theory (DFT) calculations (Table S4) [29,30]. The detailed configurations of Ti species at T6 site of polymorph Beta_A are further optimized. Tetracoordinated and H-terminated pentacoordinated Ti species might exist while H-terminated hexacoordinated Ti species undergo spontaneous transformation to pentacoordinated ones (Fig. 2D).

**Figure 3.**
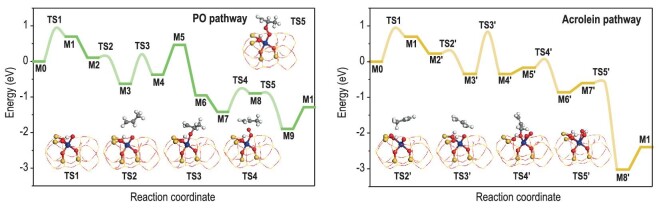
Reaction mechanism of propylene aerobic oxidation. Proposed reaction pathways, transition state structures, and the corresponding energy profiles in the formation of PO and acrolein over the H-terminated pentacoordinated Ti site in Beta zeolite at 0 K (polymorph A). Ti: dark blue, O: red, Si: yellow, H: white, C: grey.

The reaction mechanism is further interpreted with DFT calculations (Fig. 3, Figs S39, S40 and Tables S5, S6). With the H-terminated pentacoordinated Ti site, the H atom of OH* on Ti site can transfer to the adjacent O atom *via*  **TS1** (*E*_a_ = 0.92 eV, *E*_r_ = 0.70 eV), leaving O* at the Ti site. A propylene molecule adsorbs at the above O* with the C=C group close to the Ti site and the β-C atom of the CH_2_=CHCH_3_* attacks the O* to produce OCH(CH_2_)CH_3_* *via*  **TS2** (*E*_a_ = 0.00 eV, *E*_r_ = −0.74 eV). Then, the O atom of OCH(CH_2_)CH_3_* bonds with its α-C atom to form C_3_H_6_O* *via*  **TS3** (*E*_a_ = 0.83 eV, *E*_r_ = 0.26 eV) and desorbs as product, leaving one tetracoordinated Ti site. Subsequently, one dioxygen molecule adsorbs at this vacant Ti site forming Ti-OO* motif followed by the adsorption of propylene molecule. The β-C atom of the CH_2_ = CHCH_3_* attacks the terminal O atom of the Ti-OO*, which is not connected with the Ti site, to produce OOCH(CH_2_)CH_3_* *via*  **TS4** (*E*_a_ = 0.81 eV, *E*_r_ = 0.52 eV). The O atom bonded with the β-C atom in the OOCH(CH_2_)CH_3_* breaks with another O atom bonded with the Ti site and connects with the α-C atom to form C_3_H_6_O* and O* *via*  **TS5** (*E*_a_ = 0.11 eV, *E*_r_ = −1.00 eV). Once again, the formed C_3_H_6_O* desorbs as product, leaving O* at the Ti site. In the final step, the H at the O atom adjacent to the Ti site transfers back to the O* at the Ti site to close the catalytic cycle. For byproduct acrolein formation, the process of H transfer from the OH* is identical to that in the PO pathway. Starting from **M1**, a propylene molecule adsorbs above the O* atom with the −CH_3_ group close to the Ti site. One H atom of the −CH_3_ group in CH_2_=CHCH_3_* transfers to the O atom adjacent to Ti site to form CH_2_=CHCH_2_* *via*  **TS2′** (*E*_a_ = 0.06 eV, *E*_r_ = −0.57 eV). Then, the α-C atom of the CH_2_=CHCH_2_* attacks the O* at the Ti site to form OCH_2_CH=CH_2_* *via*  **TS3′** (*E*_a_ = 1.18 eV, *E*_r_ = 0.00 eV). Meanwhile, dioxygen adsorbs at the Ti site next to the OCH_2_CH=CH_2_* and one H of the −OCH_2_ group in the OCH_2_CH=CH_2_* transfers to one O atom of the O_2_* to form OCHCH=CH_2_* and O_2_H* *via*  **TS4′** (*E*_a_ = 0.21 eV, *E*_r_ = −0.69 eV). The formed OCHCH=CH_2_* desorbs from the Ti site and the H atom at the O atom adjacent to the Ti site transfers back to the O_2_H* to form H_2_O* and O* *via*  **TS5′** (*E*_a_ = 0.00 eV, *E*_r_ = −2.42 eV). Then, the H_2_O* desorbs from the Ti site to close the catalytic cycle of the side reaction. All other possible pathways competing with the formation of PO have been seriously considered and excluded (Figs S41–S45, and additional discussion in Supplementary data).

For the PO pathway at the H-terminated pentacoordinated Ti site, the process of H transfer from the OH* on the Ti site to adjacent O atom bears the highest energy barrier of 0.92 eV and is therefore recognized as the rate-determining step (RDS). Although this step also occurs in the acrolein pathway, the energy barrier of OCH_2_CH=CH_2_* formation from the α-C atom of CH_2_=CHCH_2_* attacking the O* at the Ti site is much higher (*E*_a_ = 1.18 eV), which is the RDS for byproduct acrolein formation. By comparing the energy barrier of the RDS for these two pathways, PO formation is more favorable than acrolein formation at the H-terminated pentacoordinated Ti site at 0 K, corresponding to the high PO selectivity in propylene aerobic oxidation (Fig. 1). Energy profiles at the practical reaction temperature of 813 K are given in Figs S46 and S47.

Transient kinetic analysis was employed to corroborate the proposed reaction mechanism. Both Ti-Beta and ^18^O-enriched Ti-Beta catalysts were activated and purged with He, and then the flow was shifted from He to 5% C_3_H_6_-95% He at 773 K. Immediately, PO was produced, reaching a peak before decreasing over 20 min with a yield of ∼0.08 mol_PO_/mol_Ti_ (Fig. S22). This indicates that propylene can react with the lattice oxygen of Ti-Beta catalyst to produce PO, accompanied by the formation of vacant Ti site (oxygen-deficient Ti site) after PO desorption. In the next phase, the post-reaction Ti-Beta catalyst was exposed to dioxygen (5% O_2_ in He) for chemisorption at varied temperatures. A subsequent transient kinetic analysis, switching from He to 5% C_3_H_6_-95% He at 773 K, was conducted to determine if the dioxygen chemisorption and activation in this step would foster additional PO production. Notably, the results from the second transient kinetic analysis demonstrate that PO is indeed produced in this step. Typically, 0.02–0.17 mol_PO_/mol_Ti_ can be detected on post-reaction Ti-Beta regenerated in dioxygen at 373 to 773 K, respectively (Fig. S48). That is, the step of dioxygen chemisorption and activation can be accomplished even at a low temperature of 373 K while higher temperatures are kinetically more favorable. At dioxygen regeneration temperature of 773 K, the formation peak of 0.17 mol_PO_/mol_Ti_ is resolved, nearly double the initial yield in the first step. These observations clearly suggest that our catalytic process follows the Mars-van-Krevelen mechanism, emphasizing that the vacant Ti sites formed in the initial step are ideal for dioxygen activation. The results from transient kinetic analysis align well with the mechanism predicted by DFT calculations (Fig. 3).

We also investigate the reaction mechanism of propylene epoxidation over the tetracoordinated Ti site in Beta zeolite. Tetracoordinated closed Ti site, namely Ti(SiO)_4_ is completely inactive for propylene aerobic oxidation. The H-terminated tetracoordinated Ti site, Ti(SiO)_3_OH, might catalyze the propylene aerobic epoxidation but it needs to overcome a very high energy barrier of >2 eV (C_3_H_6_O* formation from the O atom of OCH(CH_2_)CH_3_* bonding with its α-C atom *via*  **TS3**, Figs S49, S50 and Table S7). Experimentally, Ti-Beta-H with dominant tetracoordinated Ti centers (Fig. S32) is much less active than Ti-Beta with a large proportion of Ti centers in higher coordination states (Figs S10, S11), which is in good accordance with DFT simulations. The reaction mechanism of propylene aerobic epoxidation catalyzed by Ti-Beta zeolite with polymorph B and C is also investigated by DFT calculations. Briefly, H-terminated pentacoordinated Ti sites in polymorph B and C of Beta zeolites can catalyze propylene aerobic epoxidation, similar to those in polymorph A, while all other Ti sites appear to be much less active or completely inactive for the reaction (Figs S51–S61, Tables S8–S11). Ulteriorly, propylene aerobic oxidation over various Ti sites in TS-1 is investigated for comparison. Similar to the case of Ti-Beta, H-terminated pentacoordinated Ti site in TS-1 appears to be more active than H-terminated tetracoordinated Ti site for the reaction, while the tetracoordinated closed Ti site is completely inactive (Figs S62–S66, Tables S12 and S13). All these results reveal that propylene aerobic epoxidation over Ti-zeolites is a site-sensitive reaction [27,31,32]. The OH* at pentacoordinated Ti site in Beta zeolite is crucial for the remarkable performance, which participates in the catalytic cycle without consumption.

In summary, we demonstrate herein that Ti-Beta zeolites can efficiently catalyze the aerobic epoxidation of propylene, offering adequate propylene conversions and good PO selectivity at the same time. With these results, new technology for PO production, in analogy to EO production, can be expected. The Ti defective sites in the Beta zeolite framework, namely the H-terminated pentacoordinated Ti species, are identified as the preferred active sites for propylene aerobic epoxidation, with the reaction starting from the participation of lattice oxygen in Ti-OH. The unique reaction mechanism is useful for the understanding and design of other selective catalytic oxidation processes.

## MATERIALS AND METHODS

### Synthesis of Ti-Beta

Ti-Beta was synthesized *via* a two-step post-synthesis route. In a typical process, 10 g calcined H-Beta (*n*_Si_/*n*_Al_ = 13.5) was treated in 200 mL 10 M HNO_3_ at 373 K for 20 h. After dealumination, the samples were thoroughly washed with deionized water and dehydrated at 373 K overnight. The dehydrated samples denoted as Si-Beta were calcined at 823 K for 6 h with a temperature ramp of 2 K/min and adequately mixed with a specific amount of Ti(Cp)_2_Cl_2_ in the glovebox or under ambient conditions, followed by calcination at 823 K for 12 h. The as-obtained samples with different Ti contents were denoted as Ti-Beta-1%, Ti-Beta-2%, Ti-Beta-3% and Ti-Beta-4%, respectively. TiAl-Beta (2.0 wt% Ti, 1.5 wt% Al) was synthesized *via* a similar route using partially dealuminated H-Beta (dealumination for 2 h instead of 20 h) as the parent for post-synthesis modification.

### Synthesis of M-Beta

M-Beta (M = Sc, Y, Mo, W, Zr, Sn) catalysts were also synthesized *via* the two-step post-synthesis route, and metal precursors (Sc(acac)_3_, Y(NO_3_)_3_·6H_2_O, MoO_2_(acac)_2_, WCl_6_, Zr(Cp)_2_Cl_2_ and Sn(CH_3_)_2_Cl_2_) were employed instead of Ti(Cp)_2_Cl_2_.

### Characterization

Spherical aberration-corrected (*Cs*-corrected) scanning transmission electron microscopy (STEM) data were acquired using a JEOL GrandARM 300F equipped with double correctors. The microscope was equipped with a field-emission gun (FEG), two JEOL correctors, a JEOL EDS, and a Gatan quantum energy filter for spectroscopic analyses. The powder sample was dispersed in ethanol and then ultrasonicated. A few drops of the suspension were placed onto carbon copper grids. Prior to observation, the STEM corrector was aligned using a thin amorphous carbon layer, assuring a spatial resolution of 0.7 Å. The high-resolution annular dark field STEM (ADF-STEM) images were recorded at the convergence semi-angle of 16 mrad. ADF-STEM image simulations were performed using a free software package QSTEM (http://www.qstem.org), which is based on the multi-slice algorithm. Simulation parameters were roughly the same with the experimental ADF-STEM images.

Time of flight secondary ion mass spectrometry (TOF-SIMS) analyses of samples were performed on the IONTOF TOF.SIMS-5 in the Nano-X Vacuum Interconnected Nanotech Workstation at <2 × 10^−10^ mbar at SuZhou, China. The ionized secondary particles like sputtered atoms, molecules and radicals were separated by mass-charge ratio.

### Propylene aerobic epoxidation

The aerobic epoxidation of propylene was performed in a continuous flow fixed-bed quartz reactor (i.d. = 6 mm, 250 mm in length) at atmospheric pressure. The feed gas containing C_3_H_6_, O_2_ and He was controlled separately by mass flow controllers to adjust the total flow and the partial pressures of gas components. The catalyst samples were molded and sieved to collect particles (40–60 mesh) in order to limit potential mass transfer effects. In a typical experiment, 100 mg of catalyst (40–60 mesh, 0.25–0.425 mm) was placed in the constant-temperature zone of the quartz reactor and pretreated in flowing 5%O_2_/He at 673 K for 2 h. After cooling to a designated temperature, the reaction mixture containing C_3_H_6_ and O_2_ balanced by He was fed to the reactor at a total flow rate of 15−120 mL/min, corresponding to the gas hourly space velocity (GHSV) of 9000–72 000 mL/g/h. The reaction was performed step-wise warming up (20 K in each step) to obtain the catalytic data on dependences of conversion/selectivity.

### Steady-state kinetic measurement

The reaction orders were measured in the fixed-bed reactor mentioned above. The reaction order of propylene or molecular oxygen was measured by holding the total flow constant under temperatures of 793, 813 and 833 K.

### Transient kinetic analysis (TKA)

The temperature-programmed desorption (TPD), time-on-stream surface reaction and TPSR were performed on a quartz reactor equipped with a downstream gas sampling mass spectrometer (Pfeiffer Omnistar). In a typical TPD process, ∼0.2 g catalyst was fixed in the reactor and pretreated in the flowing He at 673 K. After cooling to 323 K, the sample was saturated with C_3_H_6_ or O_2_ balanced with He with a flow rate of 20 mL/min and then purged with He to remove the weakly adsorbed species. The TPD profiles were recorded in flowing He from 353 to 813 K at a heating rate of 10 K/min. In a typical time-on-stream surface reaction process, the pretreated sample was heated to a designated temperature under He and then the reaction mixture was switched to the reactor with a constant flow of 60 mL/min. The time-on-stream surface reaction profiles were recorded isothermally in flowing reaction mixture. In a typical TPSR process, the pretreated sample was cooled to 323 K and then the reaction mixture was fed to the reactor. The TPSR profiles were recorded in flowing reaction mixture from 353 to 813 K at a heating rate of 10 K/min.

## Supplementary Material

nwae305_Online_Appendix
