# Supplementary material for: Direct propylene epoxidation with molecular oxygen over titanosilicate zeolites
Source: Natl Sci Rev. 2024 Aug 27;11(11):nwae305. doi: 10.1093/nsr/nwae305 (PMC11493086; doi:10.1093/nsr/nwae305)
Supplement: nwae305_Online_Appendix [file nwae305_online_appendix.pdf]

Supplementary Materials

Direct Propylene Epoxidation with Molecular Oxygen over Titanosilicate Zeolites

Weijie Li,[1*] Bin Qin,[1*] Zhuoya Dong,[2] Yuchao Chai,[1] Guangjun Wu,[1] Yanhang Ma,[2] Meng Wang[3], Xingwu Liu[3], Ding Ma,[3†] Landong Li[1,4†]

[1] Key Laboratory of Advanced Energy Materials Chemistry of Ministry of Education, College of Chemistry, Nankai University, Tianjin 300071, P.R. China

[2] School of Physical Science and Technology, ShanghaiTech University, Shanghai 201210, China

[3] Beijing National Laboratory for Molecular Sciences, New Cornerstone Science Laboratory, College of Chemistry and Molecular Engineering, Peking University, Beijing, China.

[4] Frontiers Science Center for New Organic Matter, Nankai University, Tianjin 300071, P.R. China

[*]These authors contributed equally to this work.

[†]Corresponding authors. Email: lild@nankai.edu.cn (L. L.), dma@pku.edu.cn (D. M.)

This file includes:

Materials and Methods

Supplementary Figs. 1 to 66

Supplementary Tables 1 to 13

References (*33-70*)

Table of Contents

Materials and Methods 7

Figures and Tables 16

Supplementary Figure 1| XRD patterns of Si-Beta, $TiCp_2Cl_2$ and Ti-Beta samples. 16

Supplementary Figure 2| XRD patterns of Ti-Beta samples with different Ti contents and the interplanar d_{302} spacing of Beta zeolite as a function of Ti content. 17

Supplementary Figure 3| FTIR spectra of H-Beta, Si-Beta and Ti-Beta samples, and the enlarged-view FTIR spectra of Ti-Beta samples in the Ti-OH region. 18

Supplementary Figure 4| ^{1}H MAS NMR spectra of H-Beta, Si-Beta and Ti-Beta samples, and the enlarged-view ^{1}H NMR spectra of Ti-Beta samples in the Ti-OH region. 19

Supplementary Figure 5| ^{29}Si MAS NMR spectra of H-Beta, Si-Beta and Ti-Beta samples. 20

Supplementary Figure 6| The Ar sorption isotherms at 87 K and pore width distribution of Ti-Beta samples. 21

Supplementary Figure 7| Raman spectra of Ti-Beta and reference TiO_2 samples. 22

Supplementary Figure 8| STEM image and element mapping analyses of Ti-Beta-3% sample. 23

Supplementary Figure 9| STEM image and element mapping analyses of Ti-Beta-4% sample. 24

Supplementary Figure 10| Temperature-dependent behaviors of propylene aerobic oxidation over Ti-containing catalysts. Reaction conditions: 0.1 g catalyst, 5%C_3H_6-5%O_2-90%He, GHSV= 36000 mL/g/h. 25

Supplementary Figure 11| Temperature-dependent behavior of propylene aerobic oxidation over TiAl-Beta (2.0 wt% Ti, 1.5 wt% Al) catalyst. Reaction conditions: 0.1 g catalyst, 5%C_3H_6-5%O_2-90%He, GHSV= 36000 mL/g/h. 26

Supplementary Figure 12| Temperature-dependent behaviors of propylene aerobic oxidation over Ti-Beta catalysts. Reaction conditions: 0.1 g catalyst, 5%C_3H_6-5%O_2-90%He, GHSV= 36000 mL/g/h. 27

Supplementary Figure 13| Temperature-dependent behaviors of propylene aerobic oxidation over M-Beta catalysts. Reaction conditions: 0.1 g catalyst, 5%C_3H_6-5%O_2-90%He, GHSV= 36000 mL/g/h. 28

Supplementary Figure 14| Impacts of GHSV on propylene aerobic oxidation over Ti-Beta-3% catalyst. Reaction conditions: 0.1 g catalyst, 5%C_3H_6-5%O_2-90%He, GHSV= 9000-72000 mL/g/h. 29

Supplementary Figure 15| Impacts of C_3H_6/O_2 ratio on propylene aerobic oxidation over Ti-Beta-3% catalyst. Reaction conditions: 0.1 g catalyst, 5%C_3H_6-(2.5-20)%O_2-(92.5-75)%He, GHSV= 36000 mL/g/h. 30

Supplementary Figure 16| Process flow diagrams of HPPO and aerobic epoxidation routes. 31

Supplementary Figure 17| The flowsheet and total energy flow diagram of aerobic epoxidation route. 32

Supplementary Figure 18| Comparison of various catalysts for propylene aerobic epoxidation in terms of propylene conversion, PO selectivity and PO formation rate. Detailed information available in Supplementary Table 3. 33

Supplementary Figure 19| TPSR profiles of C_3H_6-O_2 and C_3H_6 on Ti-Beta-3% catalyst. Reaction conditions: 0.1 g catalyst, 5%C_3H_6-(0 or 5%)O_2-(95 or 90%)He, 60 mL/min. 34

Supplementary Figure 20| TPD profiles of O_2 and C_3H_6 on Ti-Beta-3%. 35

Supplementary Figure 21| TKA results of Ti-Beta catalysts. After activation and He purging of 0.1 g ^{18}O-enriched Ti-Beta-3% catalyst, the stream was switched from 60 mL/min He to 60 mL/min 5%C_3H_6-95%He, at 8 min at 773; After activation and He purging of 0.1 g Ti-Beta-3% catalyst, the stream was switched from 60 mL/min He to 60 mL/min 5%C_3H_6-95%He, at 6 min at 773 K. 36

Supplementary Figure 22| TKA results of Ti-Beta catalyst. After activation and He purging of 0.1 g Ti-Beta catalyst, the stream was switched from 60 mL/min He to 60 mL/min 5%C_3H_6-5%$^{18}O_2$-90%He, at 16 min at 673 K. 37

Supplementary Figure 23| Selectivity to PO as a function of PO formation rate over Ti-Beta-3% catalyst. 38

Supplementary Figure 24| Diagnostic tests for propylene aerobic epoxidation over Ti-Beta-3% catalyst. 39

Supplementary Figure 25| Reaction rates of PO formation as a function of C_3H_6 or O_2 concentrations catalyzed by Ti-Beta-3% at 793, 813 and 833 K. Reaction order determined by the slope of each line. 40

Supplementary Figure 26| Reaction rates of CO_x formation as a function of C_3H_6 or O_2 concentrations catalyzed by Ti-Beta-3% at 793, 813 and 833 K. Reaction order determined by the slope of each line. 41

Supplementary Figure 27| TPSR profiles of PO (A) and acrolein (B) aerobic oxidation over the Ti-Beta-3% catalyst. Reaction conditions: 0.1 g catalyst, 5%PO (or acrolein)-5%O_2-90%He, 60 mL/min. 42

Supplementary Figure 28| XRD patterns of Ti-Beta-3% before and after propylene aerobic epoxidation at 813 K for 240 h. 43

Supplementary Figure 29| Thermogravimetric analysis of Ti-Beta-3% after propylene aerobic epoxidation at 813 K for 240 h. Photographs of fresh and spent catalysts shown inset. 44

Supplementary Figure 30| Ar sorption isotherms at 87 K and pore width distribution of Ti-Beta-3% after propylene aerobic epoxidation. 45

Supplementary Figure 31| UV-vis spectra of Ti-Beta-3% before and after propylene aerobic epoxidation. 46

Supplementary Figure 32| UV-vis spectra of selected Ti-containing silicates. 47

Supplementary Figure 33| UV-vis spectra of anatase and rutile TiO_2 samples. 48

Supplementary Figure 34| TOF-SIMS analyses of dinuclear Ti species in TiO_2 and Ti-Beta samples. 49

Supplementary Figure 35| TOF-SIMS analyses of selected Ti-containing silicates. 50

Supplementary Figure 36| Simulated and experimental ADF-STEM images of Si-Beta. (A) Illustration of polymorph Beta_A structure; (B) Simulated ADF-STEM image of polymorph Beta_A structure. (Thickness: 100.3 Å); (C) Experimental ADF-STEM image of Si-Beta; (D) Enlarged-view ADF-STEM image (a 2D difference filter was applied) of Si-Beta. 51

Supplementary Figure 37| *Cs*-corrected ADF-STEM images Ti-Beta samples with different Ti contents. 52

Supplementary Figure 38| Various crystallographic T sites in Beta framework (polymorph A). 53

Supplementary Figure 39| Optimized structures involved in propylene oxidation to PO by molecular oxygen over H-terminated pentacoordinated Ti site in Beta zeolite (polymorph A). 54

Supplementary Figure 40| Optimized structures involved in propylene oxidation to acrolein by molecular oxygen over H-terminated pentacoordinated Ti site in Beta zeolite (polymorph A). 55

Supplementary Figure 41| Possible competitive reaction pathways competing with the formation of PO. 56

Supplementary Figure 42| Possible pathways competing with the formation of PO and the corresponding energy profiles-I 57

Supplementary Figure 43| Possible pathways competing with the formation of PO and the corresponding energy profiles-II 58

Supplementary Figure 44| Possible competitive reaction pathways competing with the formation of acrolein. 59

Supplementary Figure 45| Possible pathways competing with the formation of acrolein and the corresponding energy profiles. 60

Supplementary Figure 46| Calculated energy profile of propylene oxidation to PO by molecular oxygen over H-terminated pentacoordinated Ti site in Beta zeolite at 813 K (polymorph A).. 61

Supplementary Figure 47| Calculated energy profile of propylene oxidation to acrolein by molecular oxygen over H-terminated pentacoordinated Ti site in Beta zeolite at 813 K (polymorph A).. 62

Supplementary Figure 48| TKA results of C_3H_6 surface reaction on O_2-regenerated Ti-Beta-3% catalyst. After activation and He purging, the stream was switched from 60 mL/min He to 60 mL/min 5%C_3H_6-95%He, at 5-10 min at 773 K. 63

Supplementary Figure 49| Proposed reaction pathways, optimized intermediate structures, transition state structures and the corresponding energy profiles in the formation of PO and acrolein over H-terminated tetracoordinated open Ti site in Beta zeolite at 0 K (polymorph A). 64

Supplementary Figure 50| Calculated energy profile of propylene oxidation with molecular oxygen over H-terminated tetracoordinated open Ti site in Beta zeolite at 813 K (polymorph A). 65

Supplementary Figure 51| Various crystallographic T sites in Beta framework (polymorph B).. 66

Supplementary Figure 52| Structure models for the tetracoordinated Ti, H-terminated tetracoordinated Ti, and H-terminated pentacoordinated Ti sites in Beta zeolite of polymorph B (A-C) and polymorph C (D-F), as well as MFI zeolite (G-I). Ti: dark blue, O: red, Si: yellow, H: white. 67

Supplementary Figure 53| Proposed reaction pathways, optimized intermediate structures, transition state structures and the corresponding energy profiles in the formation of PO and acrolein over H-terminated pentacoordinated Ti site in Beta zeolite at 0 K (polymorph B). 68

Supplementary Figure 54| Calculated energy profile of propylene oxidation with molecular oxygen over H-terminated pentacoordinated Ti site in Beta zeolite at 813 K (polymorph B).. 69

Supplementary Figure 55| Proposed reaction pathways, optimized intermediates structures, transition state structures and the corresponding energy profiles in the formation of PO and acrolein over H-terminated tetracoordinated open Ti site in Beta zeolite at 0 K (polymorph B). 70

Supplementary Figure 56| Calculated energy profile of propylene oxidation with molecular oxygen over H-terminated tetracoordinated open Ti site in Beta zeolite at 813 K (polymorph B). 71

Supplementary Figure 57| Various crystallographic T sites in Beta framework (polymorph BEC). 72

Supplementary Figure 58| Proposed reaction pathways, optimized intermediate structures, transition state structures and the corresponding energy profiles in the formation of PO and acrolein over H-terminated pentacoordinated Ti site in Beta zeolite at 0 K (polymorph C). 73

Supplementary Figure 59| Calculated energy profile of propylene oxidation with molecular oxygen over H-terminated pentacoordinated Ti site in Beta zeolite at 813 K (polymorph C). 74

Supplementary Figure 60| Proposed reaction pathways, optimized intermediate structures, transition state structures and the corresponding energy profiles in the formation of PO and acrolein over H-terminated tetracoordinated open Ti site in Beta zeolite at 0 K (polymorph C). 75

Supplementary Figure 61| Calculated energy profile of propylene oxidation with molecular oxygen over H-terminated tetracoordinated open Ti site in Beta zeolite at 813 K (polymorph C). 76

Supplementary Figure 62| Various crystallographic T sites in MFI framework. 77

Supplementary Figure 63| Proposed reaction pathways, optimized intermediate structures, transition state structures and the corresponding energy profiles in the formation of PO and acrolein over H-terminated pentacoordinated Ti site in MFI zeolite at 0 K. 78

Supplementary Figure 64| Calculated energy profile of propylene oxidation with molecular oxygen over H-terminated pentacoordinated Ti site in MFI zeolite at 813 K. 79

Supplementary Figure 65| Proposed reaction pathways, optimized intermediate structures, transition state structures and the corresponding energy profiles in the formation of PO and acrolein over H-terminated tetracoordinated open Ti site in MFI zeolite at 0 K. 80

Supplementary Figure 66| Calculated energy profile of propylene oxidation with molecular oxygen over H-terminated tetracoordinated open Ti site in MFI zeolite at 813 K. 81

Supplementary Table 1| Texture properties of Ti-Beta zeolite samples. 82

Supplementary Table 2| Comparison between EO and PO production *via* aerobic epoxidation[4,60] 83

Supplementary Table 3| Comparison of various catalysts for propylene aerobic epoxidation 84

Supplementary Table 4| T-O distances (*r*, Å), cell volumes (V*c*, Å^3) and relative energies (ΔE, eV) for the nine distinct T sites in Ti-Beta and H-Beta (polymorph A). ... 85

Supplementary Table 5| All symbols of intermediates in the energy profile in Figure 3 and Supplementary Figs. 39-47. 86

Supplementary Table 6| Elementary reaction steps involved in the energy profile in Figure 3 and Supplementary Figs. 46-47. 87

Supplementary Table 7| Elementary reaction steps involved in the energy profile in Supplementary Figs. 49-50. 88

Supplementary Table 8| Elementary reaction steps involved in the energy profile in Supplementary Figs. 53-54. 89

Supplementary Table 9| Elementary reaction steps involved in the energy profile in Supplementary Figs. 55-56. 90

Supplementary Table 10| Elementary reaction steps involved in the energy profile in Supplementary Figs. 58-59. 91

Supplementary Table 11| Elementary reaction steps involved in the energy profile in Supplementary Figs. 60-61. 92

Supplementary Table 12| Elementary reaction steps involved in the energy profile in Supplementary Figs. 63-64. 93

Supplementary Table 13| Elementary reaction steps involved in the energy profile in Supplementary Figs. 65-66. 94

References 95

Materials and Methods

Reagents and gases

Nitric acid (HNO_3), chloroform ($CHCl_3$), triethylamine (TEA), tetrabutyl orthotitanate (TBOT), tetraethoxysilane (TEOS), tetrapropylammonium hydroxide (TPAOH, 25wt%), tetraethylammonium hydroxide (TEAOH, 35 wt%), hydrogen peroxide (H_2O_2, 35%), hydrofluoric acid (HF, 40%), titanocene dichloride ($Ti(Cp)_2Cl_2$), cyclopentene and cyclohexene were from Sigma-Aldrich. Rutile and anatase TiO_2 were provided by J&K Scientific. The metal $Sc(acac)_3$, $Y(NO_3)_3·6H_2O$, $MoO_2(acac)_2$, WCl_6, $Zr(Cp)_2Cl_2$ and $Sn(CH_3)_2Cl_2$ were from Innochem Science & Technology Co. Ltd. High purity oxygen (≥99.99%, O_2), high purity propylene (≥99.99%, C_3H_6) and He (≥99.99%) were supplied by Dalian Special Gas Co. Ltd. $^{18}O_2$ (98% enrichment) was obtained from Isotec Laboratories Inc. All the raw reagents and gases were used as received without further purification. Deionized water was home-made. H-Beta and MCM-41 samples were provided by Tianjin Shenneng Co. Ltd.

Synthesis

Synthesis of Ti-Beta. Ti-Beta was synthesized *via* a two-step post-synthesis route. In a typical process, 10 g calcined H-Beta (n_{Si}/n_{Al} = 13.5) was treated in 200 mL 10 M HNO_3 at 373 K for 20 h. After dealumination, the samples were thoroughly washed with deionized water and dehydrated at 373 K overnight. The dehydrated samples denoted as Si-Beta were calcined at 823 K for 6 h with a temperature ramp of 2 K/min and adequately mixed with specific amount of $Ti(Cp)_2Cl_2$ in the glovebox or under ambient conditions, followed by calcination at 823 K for 12 h. The as-obtained samples with different Ti contents were denoted as Ti-Beta-1%, Ti-Beta-2%, Ti-Beta-3% and Ti-Beta-4%, respectively. TiAl-Beta (2.0 wt% Ti, 1.5 wt% Al) was synthesized *via* the similar route using partially dealuminated H-Beta (dealumination for 2 h instead of 20 h) as the parent for post-synthesis modification.

Synthesis of M-Beta. M-Beta (M=Sc, Y, Mo, W, Zr, Sn) catalysts were also synthesized *via* the two-step post-synthesis route, and metal precursors ($Sc(acac)_3$, $Y(NO_3)_3·6H_2O$, $MoO_2(acac)_2$, WCl_6, $Zr(Cp)_2Cl_2$ and $Sn(CH_3)_2Cl_2$) were employed instead of $Ti(Cp)_2Cl_2$.

Synthesis of Ti-MCM-41. Ti-MCM-41 was synthesized *via* the method reported by Thomas *et al*[31]. Typically, 0.4 g calcined MCM-41 was added to the solution of 0.02 g $Ti(Cp)_2Cl_2$ and 10 mL $CHCl_3$ at room temperature under argon protection. Then,

1 mL TEA was added dropwise to the mixture and stirred for 2 h with the change of color from red to yellow. The residue from centrifugal separation was washed with $CHCl_3$ three times, dried and calcined at 823 K for 12 h.

Synthesis of TS-1. TS-1 was synthesized following the open patent[32]. Typically, TBOT was added to the TEOS dropwise under rigorous stirring in the ice water bath. A clear solution was obtained after two hours and added into TPAOH and deionized water. The final mixture with mole ratio of 0.022 TiO_2: 1.0 SiO_2: 0.35 TPAOH: 35 H_2O was stirred at 353 K for 5 h and transformed into an autoclave reactor. The reactor was rapidly heated at 448 K and kept for 48 h for static crystallization. Solid samples were washed with deionized water, dried at 373 K and calcinated at 823 K for 12 h to derive TS-1 for catalysis.

Synthesis of Ti-Beta by direct hydrothermal route. Ti-containing Beta zeolite, namely Ti-Beta-H, was synthesized *via* direct hydrothermal route as reported by Corma *et al.*[33] Typically, a gel with mole ratio of TiO_2: 60SiO_2: 32.9TEAOH: 32.9HF: 20H_2O_2: 457.5H_2O was prepared by adding TEOS, TEAOH, H_2O_2 solution, TBOT and HF solution successively under rigorous stirring and then transferred to stainless-steel autoclave for dynamic crystallization at 413 K for 8 days. Solid samples were washed with deionized water, dried at 373 K and calcinated at 823 K for 12 h to derive Ti-Beta-H for catalysis.

<u>Characterization</u>

X-ray diffraction (XRD) analysis was performed on Rigaku Smart Lab 3 kW with a HyPix-3000 detector. Diffraction patterns were recorded with Cu Kα radiation (λ=1.5418 Å, 40 mA, 40 kV) in the range of 5-50 °. The time-resolved *in situ* XRD analyses of Ti-Beta-3% during propylene aerobic epoxidation were conducted on the Rigaku Smart Lab 3 kW with the scan speed of 4 °/min. The catalyst was fixed in the reaction chamber and the reaction gas (C_3H_6/O_2/He = 5/5/90) was fed to the chamber at 813 K. The *in situ* XRD patterns were recorded every 5 h.

Fourier transform infrared spectra (FTIR) of samples were measured on a Bruker Tensor 27 spectrometer in the diffuse reflectance mode with 128 scans at a resolution of 2 cm^{-1}. Self-supporting pellets made of samples were placed in the reaction chamber and pretreated in vacuum at 573 K for 1 h. After that, the spectra were recorded against dehydrated KBr as the background.

The chemical compositions of zeolite samples were analyzed on a Thermo Fisher ICAP 7400 inductively coupled plasma optical emission spectrometer (ICP-OES). The textural properties of the samples were derived from Ar adsorption/desorption

measurement at 87 K on Quantachrome iQ-MP. The sample was outgassed under vacuum at 573 K for 3 h. The Brunauer-Emmett-Teller (BET) surface area was calculated from the adsorption data and the pore width distribution was calculated from the adsorption branches using the *t*-plot method.

UV-vis spectra of Ti-containing samples were recorded on a PerkinElmer Lambda-750 spectrometer against $BaSO_4$ as the background.

Thermogravimetric analysis (TG) of sample was performed with an SDT Q600 instrument with flowing dry air from 300 to 1200 K at a heating rate of 10 K/min.

Raman spectra of Ti-containing samples were recorded on a high-resolution, RTS-B Raman system excited by Nd:YAG laser (532 nm, output power of 50 mW).

^{1}H solid-state nuclear magnetic resonance (NMR) experiments were performed on a Bruker Avance III 400WB spectrometer at resonance frequencies of 400.1 MHz. Single pulse excitation with repetition time of 20 s and a sample spinning rate of 10 kHz were employed. ^{1}H NMR measurements were performed using dehydrated samples, which were dehydrated at 673 K at a pressure below 10^{-2} Pa for 12 h. ^{29}Si NMR spectra of zeolite samples were recorded at resonance frequencies of 79.5 MHz. The ^{29}Si spectra were referenced to tetramethyl-silane.

Spherical aberration-corrected (*Cs*-corrected) scanning transmission electron microscopy (STEM) data were acquired using a JEOL GrandARM 300F equipped with double correctors. The microscope was equipped with a field-emission gun (FEG), two JEOL correctors, a JEOL EDS, and a Gatan quantum energy filter for spectroscopic analyses. The powder sample was dispersed in ethanol and then ultrasonicated. Few drops of the suspension were placed onto carbon copper grids. Prior to observation, the STEM corrector was aligned using a thin amorphous carbon layer, assuring a spatial resolution of 0.7 Å. The high-resolution annular dark field STEM (ADF-STEM) images were recorded at the convergence semi-angle of 16 mrad. ADF-STEM image simulations were performed using a free software package QSTEM (http://www.qstem.org), which is based on the multi-slice algorithm. Simulation parameters were roughly same with the experimental ADF-STEM images.

Time of flight secondary ion mass spectrometry (TOF-SIMS) analyses of samples were performed on the IONTOF TOF.SIMS-5 in the Nano-X Vacuum Interconnected Nanotech Workstation at $<2*10^{-10}$ mbar at SuZhou, China. The ionized secondary particles like sputtered atoms, molecules and radicals were separated by mass-charge ratio.

Catalysis

Propylene aerobic epoxidation. The aerobic epoxidation of propylene was performed in a continuous flow fixed-bed quartz reactor (i.d. = 6 mm, 250 mm in length) at atmospheric pressure. The feed gas containing C_3H_6, O_2 and He was controlled separately by mass flow controllers to adjust the total flow and the partial pressures of gas components. The catalyst samples were molded and sieved to collect particles (40-60 mesh) in order to limit potential mass transfer effects. In a typical experiment, 100 mg of catalyst (40-60 mesh, 0.25-0.425 mm) was placed in the constant-temperature zone of the quartz reactor and pretreated in flowing 5%O_2/He at 673 K for 2 hours. After cooling down to designated temperature, the reaction mixture containing C_3H_6 and O_2 balanced by He was fed to the reactor at a total flow rate of 15-120 mL/min, corresponding to the gas hourly space velocity (GHSV) of 9000-72000 mL/g/h. The reaction was performed step-wise warming up (20 K in each step) to obtain the catalytic data on dependences of conversion/selectivity.

The outlet gas composition was analyzed online by gas chromatograph (Techcomp GC7900, equipped with a Porapak Q packed column, a nickel conversion furnace, and a flame ionization detector (FID), together with a gas chromatograph SP-7890-plus equipped with FID and TCD dual-detector, and further confirmed by the mass spectrometer (Pfeiffer Omnistar GSD 320). The reactant conversion, selectivity to the products and the PO formation rate were calculated based on corrected peak area normalization method (the difference in carbon number in each product was considered.):

$$\text{Corrected Peak Area } A_i = A_i' \, F_i \, v_i$$

Where *Ai* is the relative quantity of the product *i*, *Ai'* is the peak area directly obtained by GC, *Fi* is the relative correction factor based on propylene as a standard and *vi* is the carbon number of reactants or products.

Conversion of propylene was calculated as follow:

$$Conversion\ (\%) = \frac{\sum_i A_i}{\sum_i A_i + A_{propylene}} \times 100\%$$

Selectivity of various products was calculated as follow:

$$Selectivity\ of\ i\ (\%) = \frac{A_i}{\sum_i A_i} \times 100\%$$

Formation rate of PO was calculated as follow:

$$Formation\ rate = \frac{Flow_{propylene} * Pressure * Conversion * PO\ selectivity}{R \cdot T \cdot Mass_{catalyst}}$$

The carbon balance, including propylene and all oxygen-containing products (PO,

acrolein, CO_x), was over 96% for all catalytic tests conducted. Traces of propylene oligomers (C_6, C_9...) were detected at high reaction temperatures. Carbon balance was calculated as follow.

$$Carbon\ balance = \frac{3 * n_{propylene\ outlet} + 3 * n_{PO\ outlet} + 3 * n_{acrolein\ outlet} + n_{COx\ outlet}}{3 * n_{propylene\ inlet}} * 100\%$$

Related reaction equations[2, 34]:

$$C_3H_6(g) + \frac{1}{2}O_2(g) = C_3H_6O(l);\ \Delta H^o_{298\ K} = -113.2\ kJ/mol$$

$$C_3H_6(g) + \frac{9}{2}O_2(g) = 3CO_2(g) + 3H_2O(l);\ \Delta H^o_{298\ K} = -2049.0\ kJ/mol$$

$$C_3H_4O(l) + \frac{7}{2}O_2(g) = 3CO_2(g) + 2H_2O(l);\ \Delta H^o_{298\ K} = -1625.7\ kJ/mol$$

$$C_3H_6O(l) + 4O_2(g) = 3CO_2(g) + 3H_2O(l);\ \Delta H^o_{298\ K} = -1915.8\ kJ/mol$$

Free of heat and mass transfer limitations[35-38]:

First, we confirm the plug-flow behaviors by the following relations:

$$\frac{d_t}{d_p} = \frac{6 * 10^{-3}\ m}{300 * 10^{-6}\ m} = 20 > 10$$

$$\frac{L_{cb}}{d_p} = \frac{1.8 * 10^{-2}\ m}{300 * 10^{-6}\ m} = 60 > 50$$

where d_t is the reactor tube diameter, d_p is the particle diameter and L_{cb} is the catalyst bed length.

For propylene aerobic epoxidation with Ti-Beta catalyst, the diameters of reactants propylene, dioxygen and product PO are 4.0, 3.4 and 4.3 Å, respectively, which are distinctly smaller than the pore width of Beta zeolite (6.0 Å). Hence, the exploration of the reaction kinetics is theoretically feasible.

Mears criterion for interphase heat transfer limitations[39]:

$$\frac{|\Delta H_R| r_{obs} R_p E_a}{R_g h T_b^2} < 0.15$$

Where ΔH_R is the reaction heat (J/mol), r_{obs} is the rate per catalyst volume (mol/m^3/s), R_p is the particle radius, E_a is the activation energy (J/mol), R_g is the ideal gas constant (J/mol/K), h is the gas-solid heat transfer coefficient (W/m^2/K) and T_b is the temperature of bulk fluid (K).

$$h = \frac{2\lambda_g}{d_p}$$

Where λ_g is the thermal conductivity of the gas

$$\lambda_g = y_{C_3H_6}\lambda_{C_3H_6} + y_{O_2}\lambda_{O_2} + y_{He}\lambda_{He} = 0.05(0.0379) + 0.05(0.028) + 0.9(0.165) =$$

$$0.1518\ \mathrm{W/m/K}\ \ (0.1542@773\ \mathrm{K})$$

$$\text{giving}\ \ h = \frac{2*0.1518}{300*10^{-6}} = 1012\ \mathrm{W/m^2/K}\ \ (1028@773\ \mathrm{K})$$

Where ΔH_R = -113.2*10^3 J/mol, r_{obs} = 0.573 mol/m^3/s (1.833 mol/m^3/s @773 K), R_p = 1.5*10^{-4} m, E_a = 82*10^3 J/mol, R_g = 8.314 J/mol/K, T_b = 713 (773) K.

Mears criterion at 713 K:

$$\frac{|\Delta H_R| r_{obs} R_p E_a}{R_g h T_b^2} = \frac{113.2 * 10^3 * 0.573 * 1.5 * 10^{-4} * 82 * 10^3}{8.314 * 1012 * 713^2} \approx 1.862 * 10^{-4} < 0.15$$

Therefore, our experimental conditions can satisfy the Mears criterion for the absence of heat transfer limitations (at 773 K Mears criterion is 4.977*10^{-4}< 0.15).

Mears criterion for mass transport limitations:

$$\frac{r_{obs} R_p}{k_c C_{Ab}} < 0.15$$

where k_c is the mass transfer coefficient of the reactant (m/s) and C_{ab} is the concentration of the reactant in the bulk gas phase (mol/m^3).

$$k_c \sim \frac{2 D_{C_3H_6-He}}{d_p}$$

$D_{C_3H_6-He}$ – diffusion coefficient of C_3H_6 in He (m^2/s).

$$D_{C_3H_6-He} = \frac{0.0101 {T_b}^{1.75} \sqrt{\frac{1}{M_{He}} + \frac{1}{M_{C_3H_6}}}}{P[(\sum v_{He})^{\frac{1}{3}} + (\sum v_{C_3H_6})^{\frac{1}{3}}]^2} = 1.826 * 10^{-4}\ \mathrm{m^2/s}\ (2.103 * 10^{-4} @773K)$$

$$k_c = \frac{2 * 1.826 * 10^{-4}}{300 * 10^{-6}} = 1.217\ m/s\ \ (1.402@773\ \mathrm{K}).\ \ C_{Ab} = 0.78\ \mathrm{mol/m^3}\ (0.69@773\ K)$$

$$\frac{r_{obs} R_p}{k_c C_{Ab}} = \frac{0.573 * 150 * 10^{-6}}{1.217 * 0.78} = 9.05 * 10^{-5} < 0.15$$

Therefore, our experimental conditions can satisfy the Mears criterion for the absence of external mass transfer limitations (at 773 K Mears criterion is 2.84*10^{-4} < 0.15).

Experimental diagnostic tests

To further validate that the catalytic data were obtained in the kinetic regime, we performed additional diagnostic tests using our most active catalyst Ti-Beta-3%. We adjusted the amount of catalyst loaded in the reactor and the overall flow at the same time, to keep the constant ratio between the flow rate and catalyst weight. As shown in **Supplementary Fig. 24** the reaction rate profiles are nearly identical, indicating the reaction kinetics not affected by the transport phenomena.

Steady-state kinetic measurement

The reaction orders were measured in the fixed-bed reactor mentioned above. The reaction order of propylene or molecular oxygen was measured by holding the total flow constantly under temperatures of 793, 813 and 833 K.

Transient kinetic analysis (TKA)

The temperature-programmed desorption (TPD), time-on-stream surface reaction and temperature-programmed surface reaction (TPSR) were performed on a quartz reactor equipped with a downstream gas sampling mass spectrometer (Pfeiffer Omnistar). In a typical TPD process, *ca.* 0.2 g catalyst was fixed in the reactor and pretreated in the flowing He at 673 K. After cooling down to 323 K, the sample was saturated with C_3H_6 or O_2 balanced with He with a flow rate of 20 mL/min and then purged with He to remove the weakly adsorbed species. The TPD profiles were recorded in flowing He from 353 to 813 K at a heating rate of 10 K/min. In a typical time-on-stream surface reaction process, the pretreated sample was heated to designated temperature under He and then the reaction mixture was switched to the reactor with a constant flow of 60 mL/min. The time-on-stream surface reaction profiles were recorded in flowing reaction mixture isothermally. In a typical TPSR process, the pretreated sample was cooled down to 323 K and then the reaction mixture was fed to the reactor. The TPSR profiles recorded in flowing reaction mixture from 353 to 813 K at a heating rate of 10 K/min.

Computational Methods

All spin-polarized DFT calculations were performed using the Vienna ab initio simulation package (VASP)[40,41]. The electron-ion interaction was described by the Perdew–Burke–Ernzerhof (PBE) exchange–correlation functional[42] and the projector-augmented wave (PAW) potentials[43]. The Bayesian error estimation functional with van der Waals correlation (BEEF-vdW) and an energy cut-off of 400 eV were employed in this work. Γ point was used to optimize all structures[44]. The convergence criterion of the electronic energy of the supercell was 10^{-4} eV and that of the force on all unconstrained atoms was 0.03 eV Å. The zero-point energies (ZPE), enthalpies, entropies, and Gibbs free energies were calculated from harmonic frequencies[45]. The climbing image nudged elastic band (CI-NEB) method was used to find transition states, which was further confirmed by harmonic frequencies[46,47].

The Ti-Beta zeolite of polymorph A was represented by a tetragonal 64T unit cell with optimized lattice constants (a = b = 12.65 Å, c = 26.23 Å; α=β=γ=90 °), consistent with the experimental data (a = b = 12.66 Å, c = 26.40 Å; α=β=γ=90 °)[48]. Atomic positions were fixed to their crystallographic positions before the lattice constants of Beta zeolite were optimized. Then, global optimization was performed with the optimized lattice constants. There are 9 crystallographic T

sites in the framework of polymorph Beta_A shown in **Supplementary Fig. 38**. Si atoms in 9 crystallographic T sites are replaced by Ti atoms in sequence to obtain 9 Ti-Beta structures, of which the structure optimization was performed respectively. Ti-O distances, cell volumes and relative energies of the 9 crystallographic T sites in these Ti-Beta structures are shown in **Supplementary Table 4**. The T6 site in the Ti-Beta structure is the most stable. The tetracoordinated Ti, H-terminated tetracoordinated Ti, H-terminated pentacoordinated and H-terminated hexacoordinated Ti sites are shown in **Fig. 2D** and the subsequent reaction mechanisms on these sites are calculated based on the T6 site in Ti-Beta structure.

The Ti-Beta zeolite of polymorph B was represented by a monoclinic 64T unit cell with the optimized lattice constants of a = 17.90 Å, b = 17.92 Å, and c = 14.33 Å; α=γ=90 °, β=114.8 °, consistent with the experimental data (a = 17.90 Å, b = 17.92 Å, and c = 14.33 Å; α=γ=90 °, β=114.8 °)[48]. There are also 9 crystallographic T sites in the framework of polymorph Beta_B shown in **Supplementary Fig. 51**. Si atoms in 9 crystallographic T sites are replaced by Ti atoms in sequence to obtain 9 Ti-Beta structures, of which the structure optimization was performed respectively. The T1 site in the Ti-Beta structure is the most stable. According to T1 site in the Ti-Beta structure, the tetracoordinated Ti, H-terminated tetracoordinated Ti, and H-terminated pentacoordinated Ti species are shown in **Supplementary Fig. 52** and the subsequent reaction mechanisms on the H-terminated tetracoordinated Ti and H-terminated pentacoordinated Ti species are calculated.

The Ti-Beta zeolite of polymorph C was represented by a tetragonal 32T unit cell with the optimized lattice constants of a = b = 12.76 Å, c = 13.01 Å; α=β=γ=90 °, consistent with the experimental data (a = b = 12.82 Å, c = 13.34 Å; α=β=γ=90 °)[49]. There are 3 crystallographic T sites in the framework of BEC shown in **Supplementary Fig. 57**. Si atoms in 3 crystallographic T sites are replaced by Ti atoms in sequence to obtain 3 Ti-Beta structures, of which the structure optimization was performed respectively. The T1 site in the Ti-Beta structure is the most stable. According to T1 site in the Ti-Beta structure, the tetracoordinated Ti, H-terminated tetracoordinated Ti, and H-terminated pentacoordinated Ti species are shown in **Supplementary Fig. 52** and the subsequent reaction mechanisms on the H-terminated tetracoordinated Ti and H-terminated pentacoordinated Ti species are calculated.

The MFI zeolite was represented by an orthorhombic 96T unit cell with the optimized lattice constants of a = 20.24 Å, b = 19.89 Å, and c = 13.33 Å; $\alpha=\beta=\gamma=90$ °, consistent with the experimental data (a = 20.93 Å, b = 19.93 Å, and c = 13.40 Å; $\alpha=\beta=\gamma=90$ °)[50]. There are 12 crystallographic T sites in the MFI framework shown in **Supplementary Fig. 62**. Si atoms in 12 crystallographic T sites are replaced by Ti atoms in sequence to obtain 12 TS-1 structures, of which the structure optimization was performed respectively. The T7 and T4 site in the MFI framework is stable, and T7 is the most populated sites on the basis of neutron diffraction data[50]. According to T7 site in the MFI framework, the tetracoordinated Ti, H-terminated tetracoordinated Ti, and H-terminated pentacoordinated Ti species are shown in **Supplementary Fig. 52** and the subsequent reaction mechanisms on the H-terminated tetracoordinated Ti and H-terminated pentacoordinated Ti species are calculated.

Figures and Tables

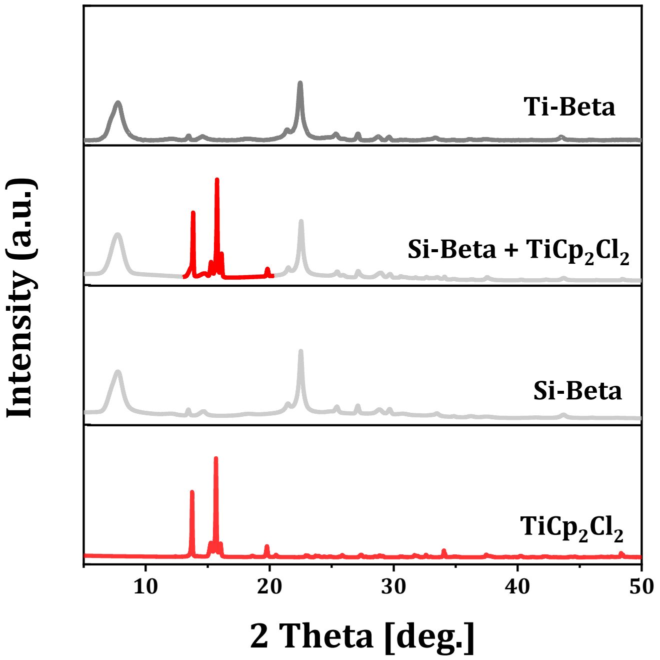

Supplementary Figure 1| XRD patterns of Si-Beta, $TiCp_2Cl_2$ and Ti-Beta samples.

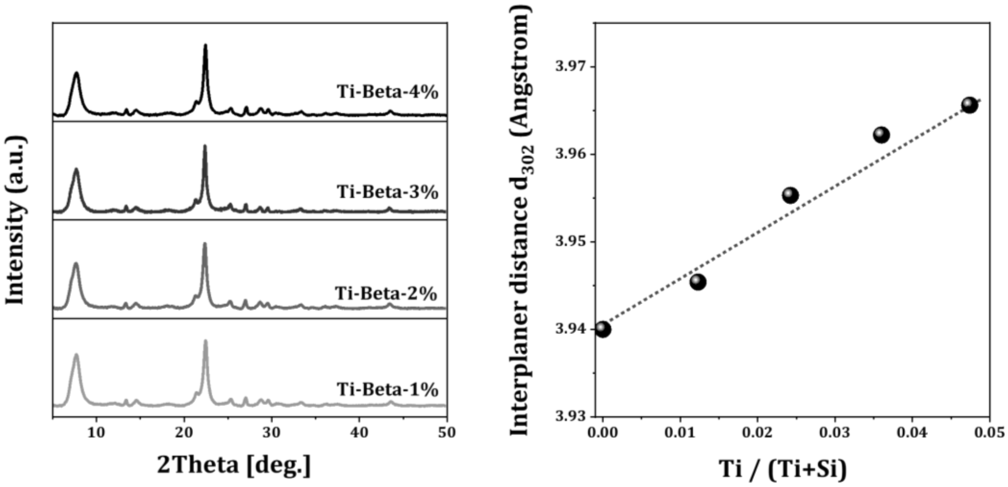

Supplementary Figure 2| XRD patterns of Ti-Beta samples with different Ti contents and the interplanar d_{302} spacing of Beta zeolite as a function of Ti content.

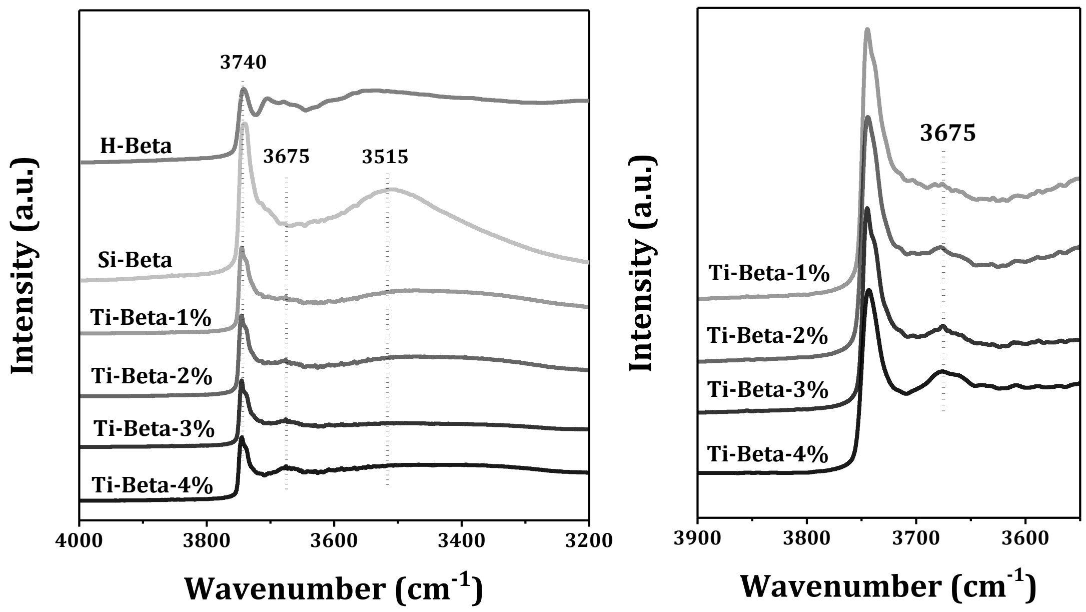

Supplementary Figure 3| FTIR spectra of H-Beta, Si-Beta and Ti-Beta samples, and the enlarged-view FTIR spectra of Ti-Beta samples in the Ti-OH region.

Note: All the spectra have been normalized according to the peaks at 1865 cm^{-1} for comparison. The FTIR spectrum of H-Beta shows serval IR bands attributed to Al-OH groups (3660 cm^{-1}), bridging acidic hydroxyls Si-OH-Al (3610 cm^{-1}) and isolated Si-OH groups (3740 cm^{-1}), respectively[51]. Through dealumination, the bands related to Al-OH and Si-OH-Al groups disappear while the bands related to isolated silanol groups at 3740 cm^{-1} get intensified accompanied by the appearance of IR bands at 3515 cm^{-1} related to associated Si-OH groups. Upon Ti incorporation, the intensities of bands related to isolated Si-OH groups decrease distinctly and the bands related to associated Si-OH groups almost disappear due to the formation of Si-O-Ti linkages between Ti and Si-OH. Meanwhile, new weak IR bands at 3675 cm^{-1} due to Ti-OH groups appear[52-54].

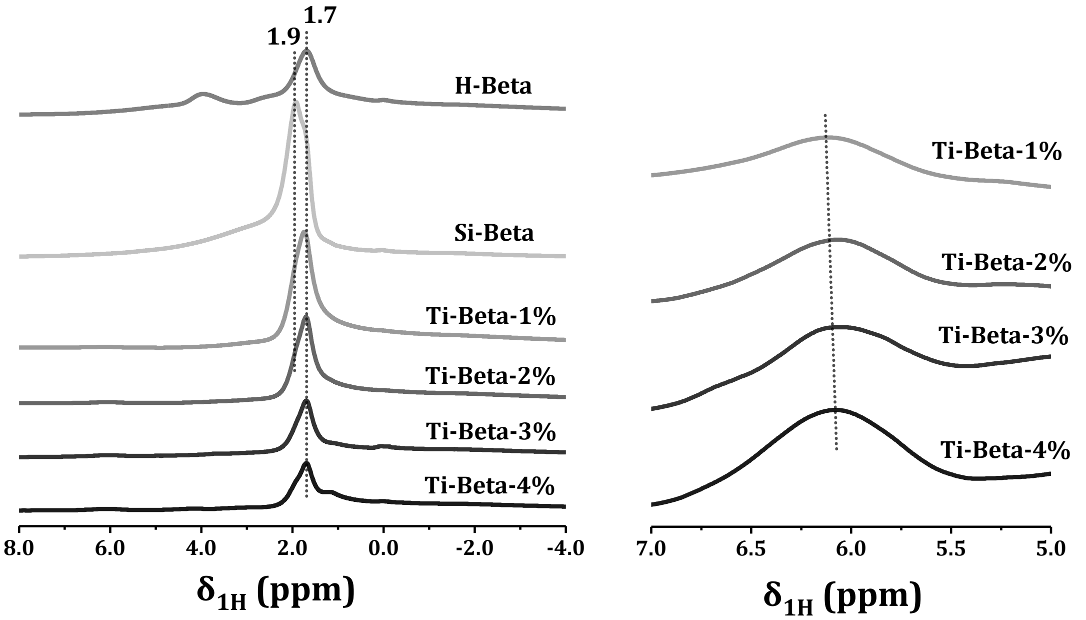

Supplementary Figure 4| ^{1}H MAS NMR spectra of H-Beta, Si-Beta and Ti-Beta samples, and the enlarged-view ^{1}H NMR spectra of Ti-Beta samples in the Ti-OH region.

Note: The ^{1}H MAS NMR spectrum of H-Beta shows serval signals attributed to Al-OH groups (2.5 ppm), bridging acidic hydroxyls Si-OH-Al (3.9 ppm) and Si-OH groups (1.7 ppm), respectively[55]. Through dealumination, the signals related to Al-OH and Si-OH-Al groups disappear while the signals related to isolated silanol groups at 1.7 ppm get intensified accompanied by the appearance of signals at 1.9 ppm related to associated Si-OH groups. Upon Ti incorporation, the intensities of signals related to isolated Si-OH decrease distinctly and the signals related to associated Si-OH groups almost disappear. Meanwhile, new signals at ~6.1 ppm due to Ti-OH groups appear[56-58].

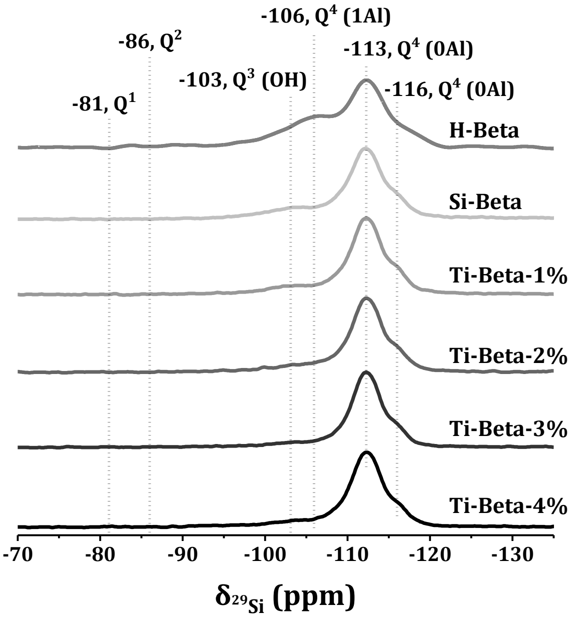

Supplementary Figure 5| ^{29}Si MAS NMR spectra of H-Beta, Si-Beta and Ti-Beta samples.

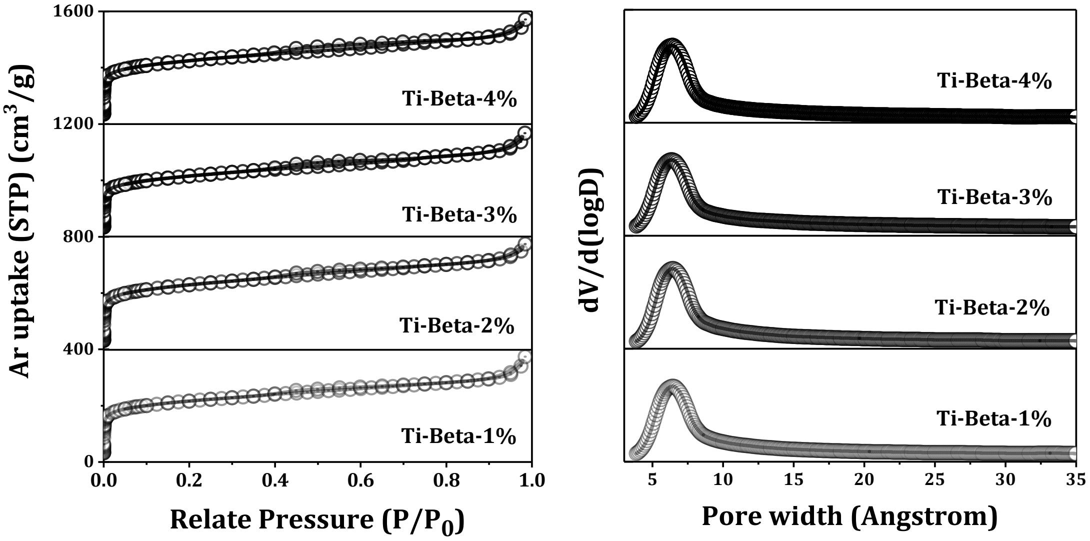

Supplementary Figure 6| The Ar sorption isotherms at 87 K and pore width distribution of Ti-Beta samples.

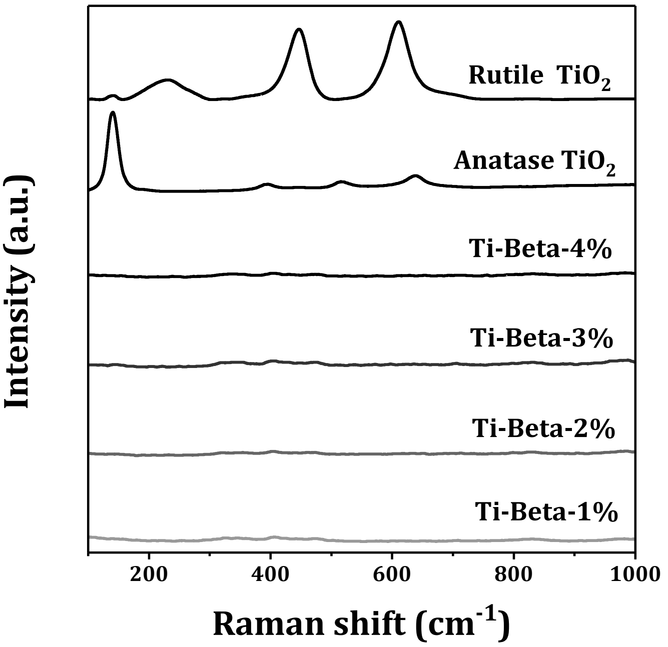

Supplementary Figure 7| Raman spectra of Ti-Beta and reference TiO_2 samples.

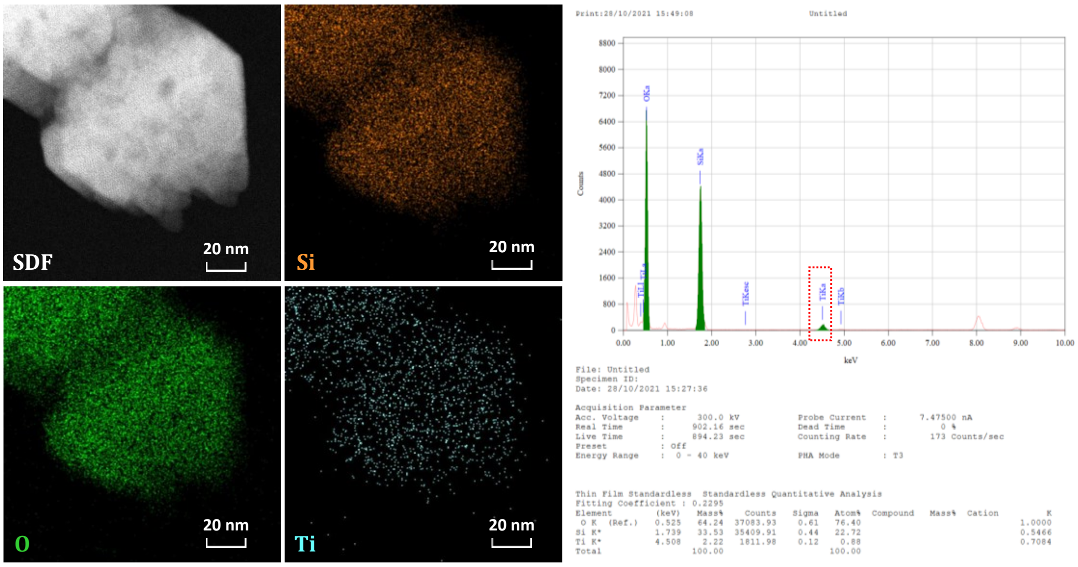

Supplementary Figure 8| STEM image and element mapping analyses of Ti-Beta-3% sample.

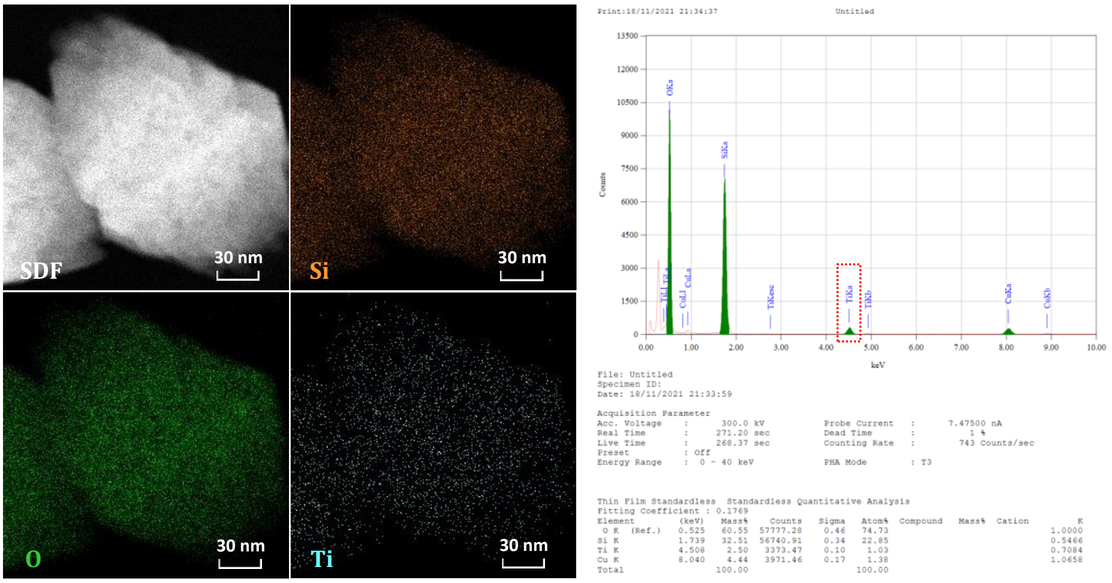

Supplementary Figure 9| STEM image and element mapping analyses of Ti-Beta-4% sample.

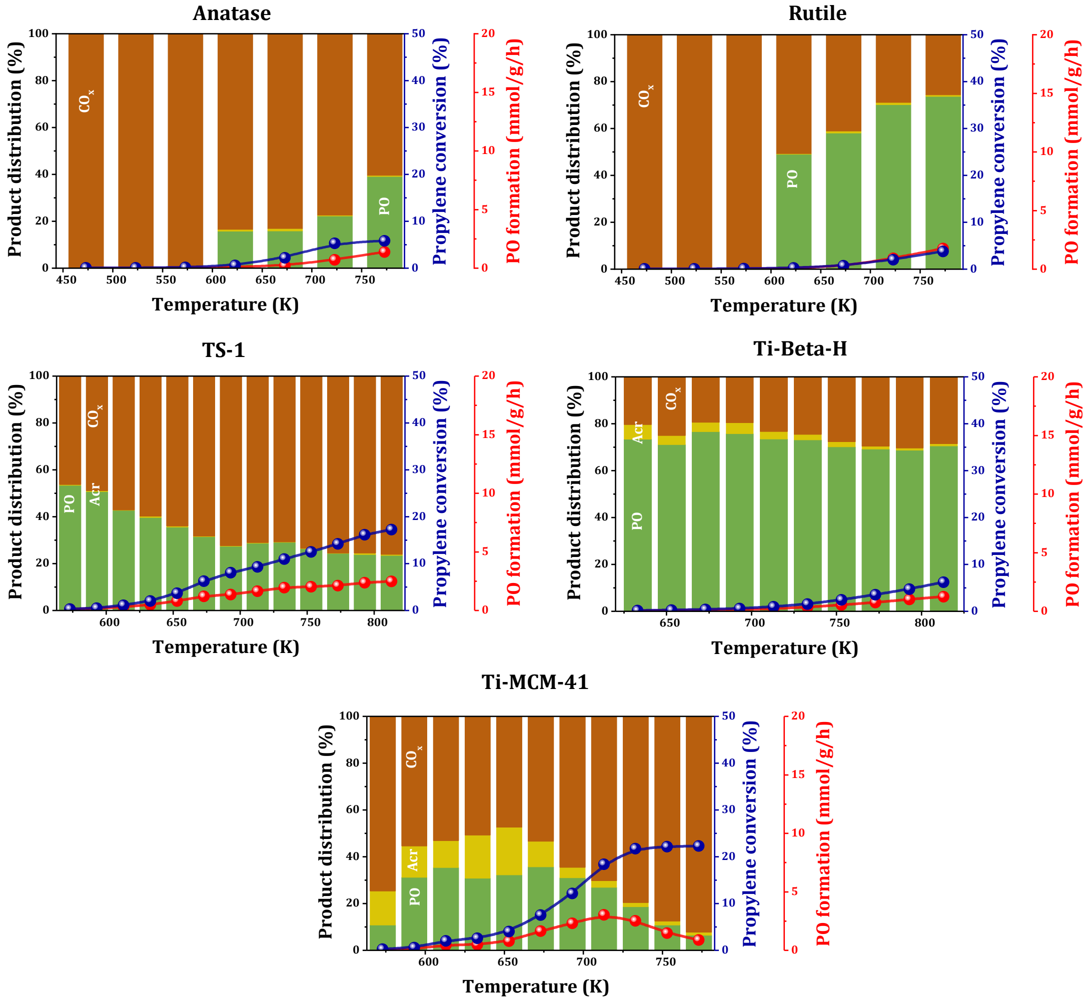

Supplementary Figure 10| Temperature-dependent behaviors of propylene aerobic oxidation over Ti-containing catalysts. Reaction conditions: 0.1 g catalyst, 5%C_3H_6-5%O_2-90%He, GHSV= 36000 mL/g/h.

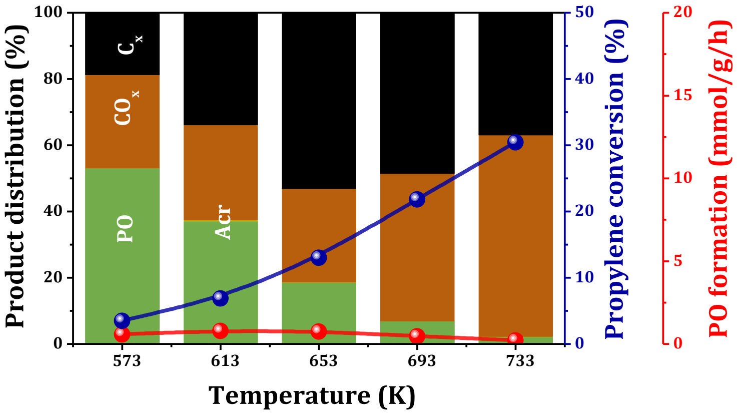

Supplementary Figure 11| Temperature-dependent behavior of propylene aerobic oxidation over TiAl-Beta (2.0 wt% Ti, 1.5 wt% Al) catalyst. Reaction conditions: 0.1 g catalyst, 5%C_3H_6-5%O_2-90%He, GHSV= 36000 mL/g/h.

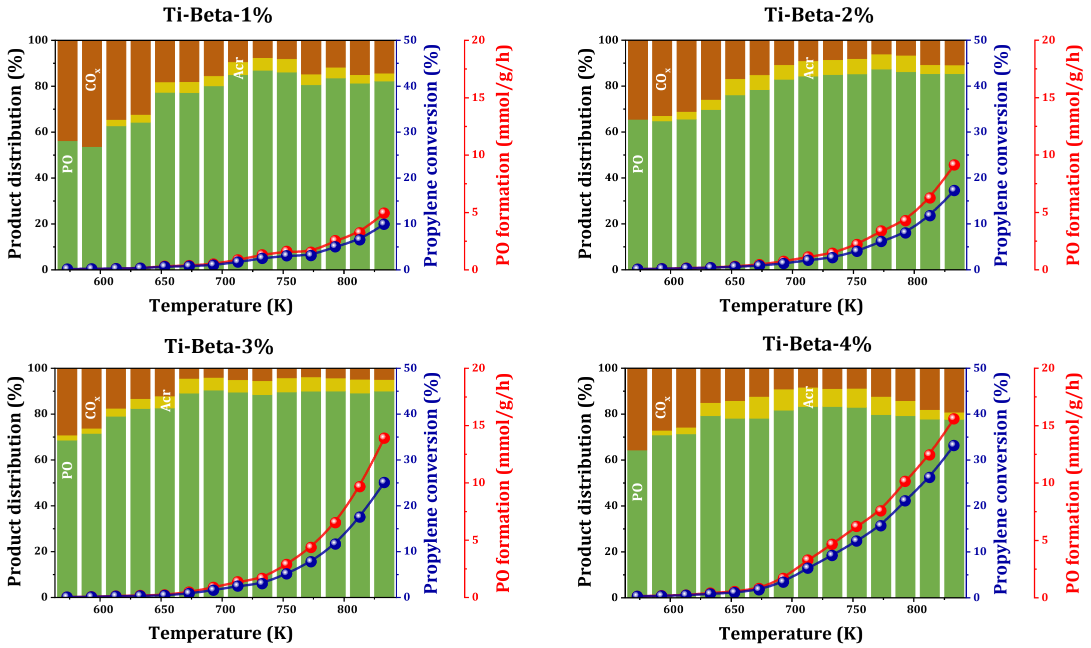

Supplementary Figure 12| Temperature-dependent behaviors of propylene aerobic oxidation over Ti-Beta catalysts. Reaction conditions: 0.1 g catalyst, 5%C_3H_6-5%O_2-90%He, GHSV= 36000 mL/g/h.

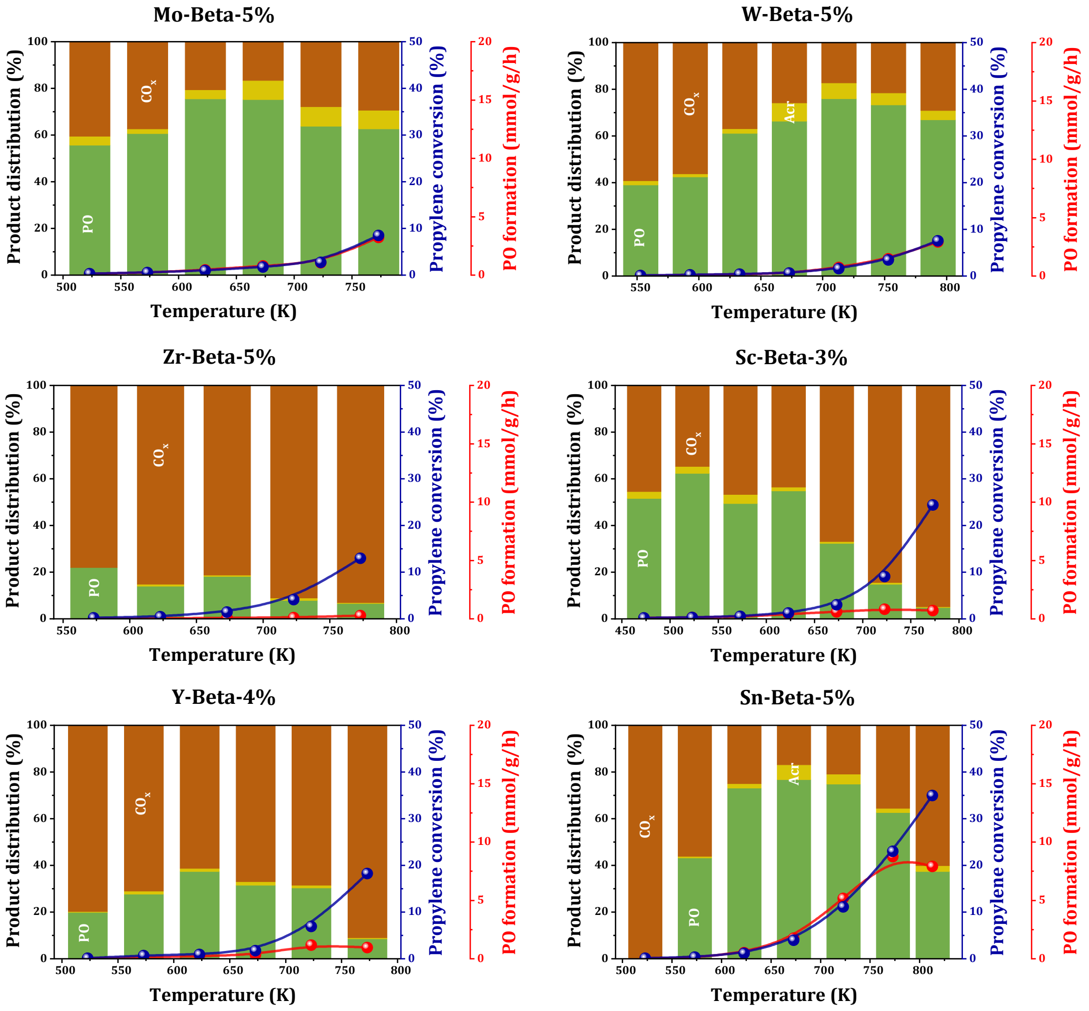

Supplementary Figure 13| Temperature-dependent behaviors of propylene aerobic oxidation over M-Beta catalysts. Reaction conditions: 0.1 g catalyst, 5%C_3H_6-5%O_2-90%He, GHSV= 36000 mL/g/h.

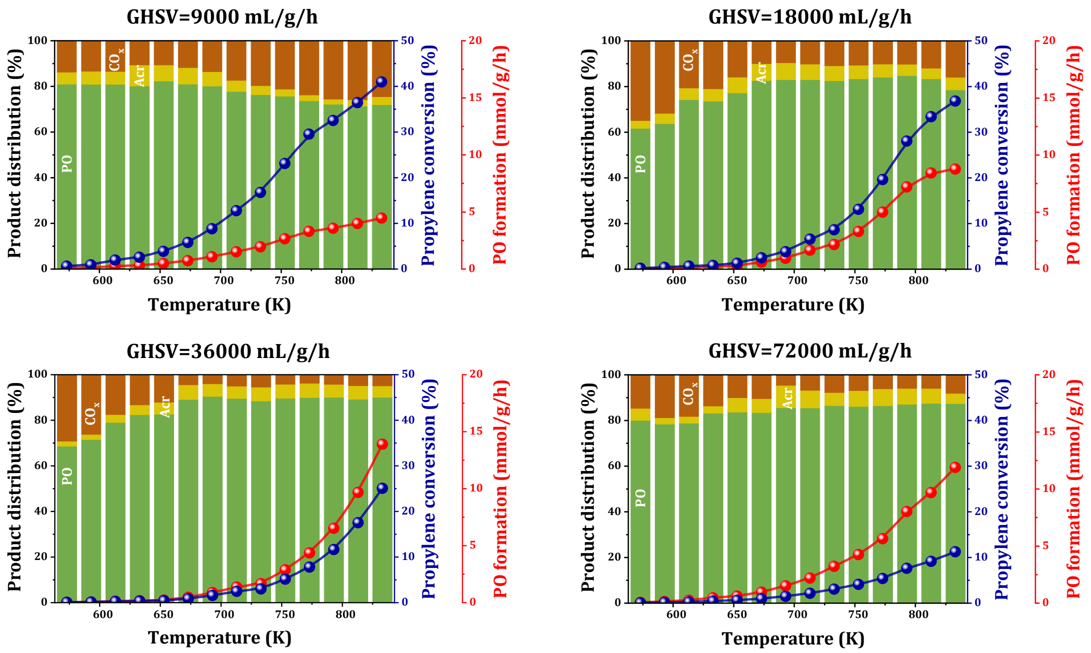

Supplementary Figure 14| Impacts of GHSV on propylene aerobic oxidation over Ti-Beta-3% catalyst. Reaction conditions: 0.1 g catalyst, 5%C_3H_6-5%O_2-90%He, GHSV= 9000-72000 mL/g/h.

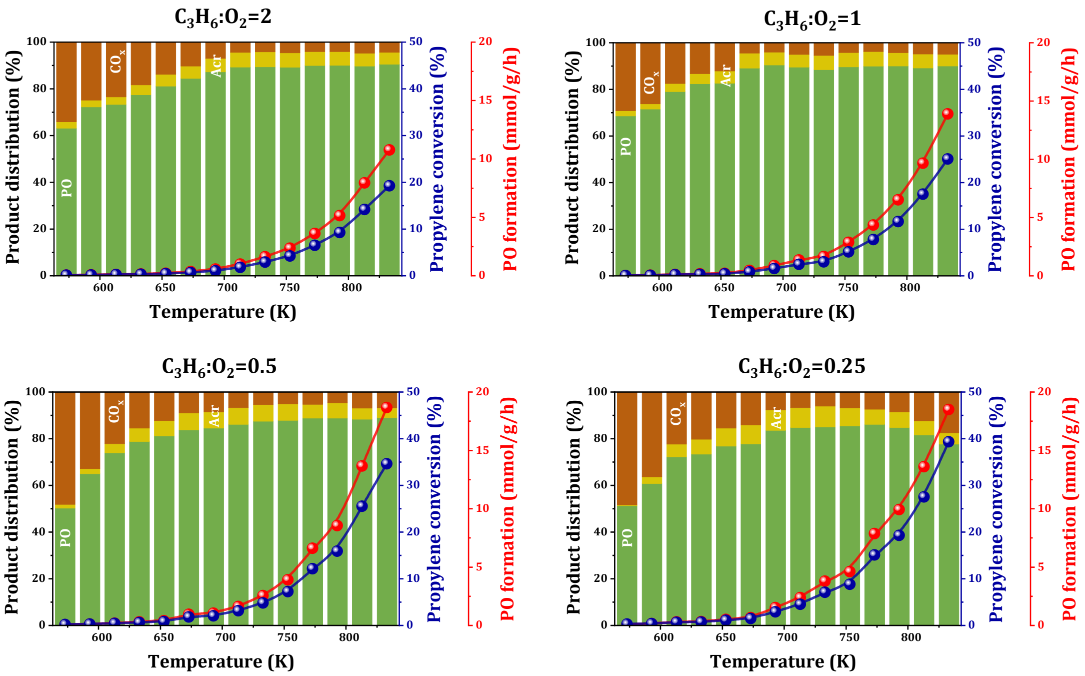

Supplementary Figure 15| Impacts of C_3H_6/O_2 ratio on propylene aerobic oxidation over Ti-Beta-3% catalyst. Reaction conditions: 0.1 g catalyst, 5%C_3H_6-(2.5-20)%O_2-(92.5-75)%He, GHSV= 36000 mL/g/h.

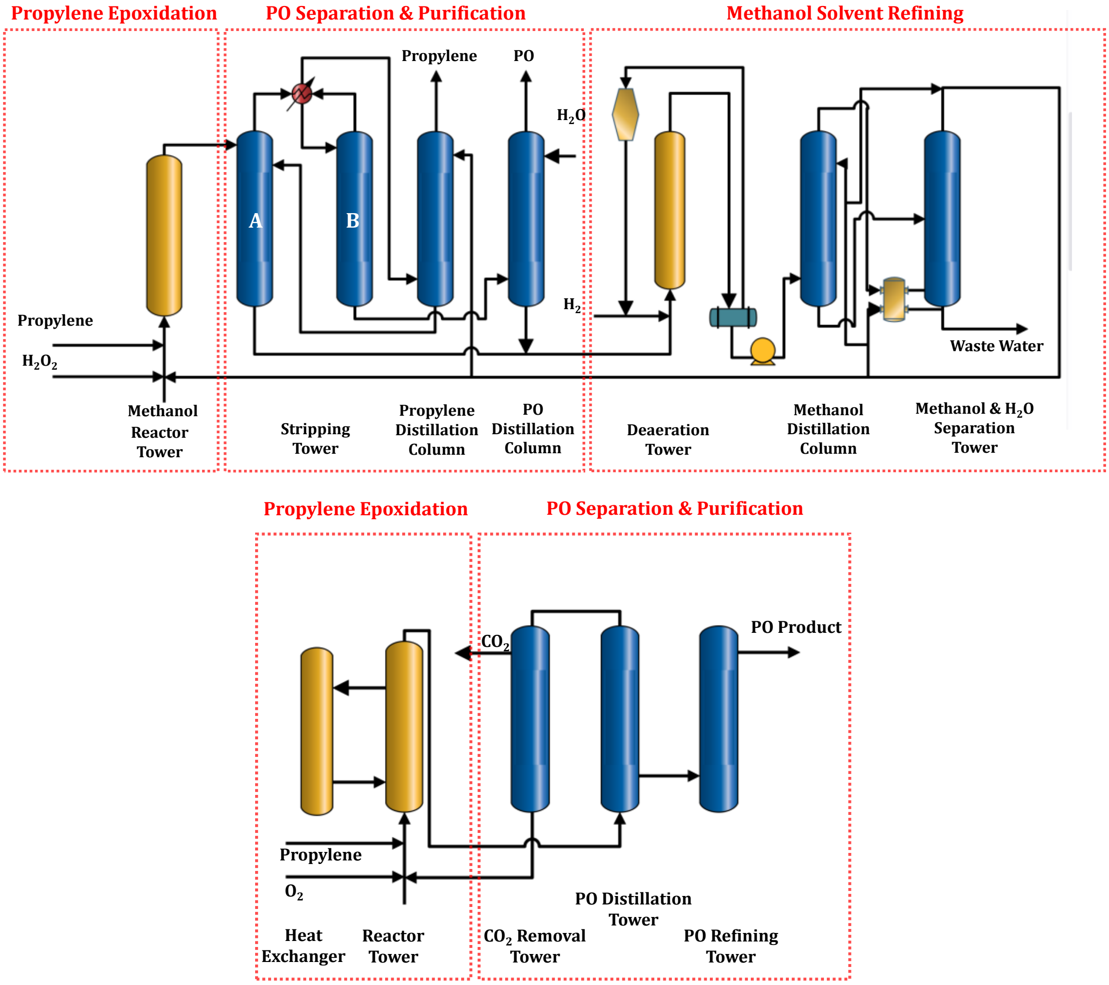

Supplementary Figure 16| Process flow diagrams of HPPO and aerobic epoxidation routes.

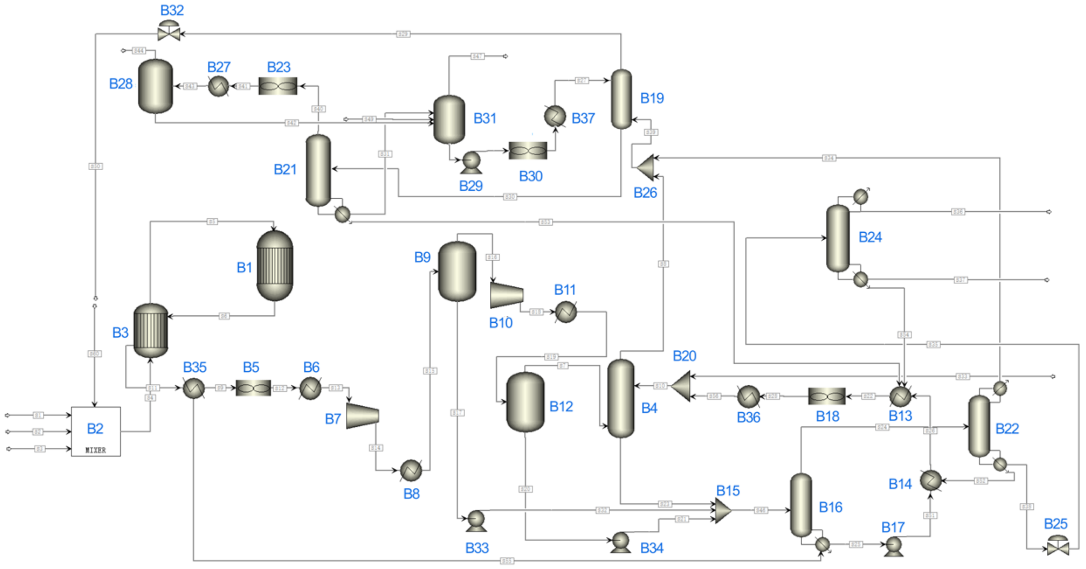

Supplementary Figure 17| The flowsheet and total energy flow diagram of aerobic epoxidation route.

Note: The simulation was based on defined material and energy balances of propylene epoxidation over Ti-Beta, with consideration for reaction kinetics, mass transfer, and heat transfer phenomena. The total energy consumption of propylene epoxidation was calculated to be 1239 kgce per ton PO under 20,000-ton classification apparatus. Sensitivity analyses were conducted to investigate the influence of various parameters on the overall process performance. The energy consumption of propylene epoxidation was predominantly influenced by the refinement efficiency of PO, which could be lessened by increasing the concentration of propylene reactant, enhancing the separation efficiency, and establishing continuous production methodology such as PO to propylene glycol. It is crucial to highlight that since the aerobic epoxidation method is still in its early stages, there is substantial potential for further development and optimization. This will likely lead to further reductions in energy consumption and enhanced the process efficiency.

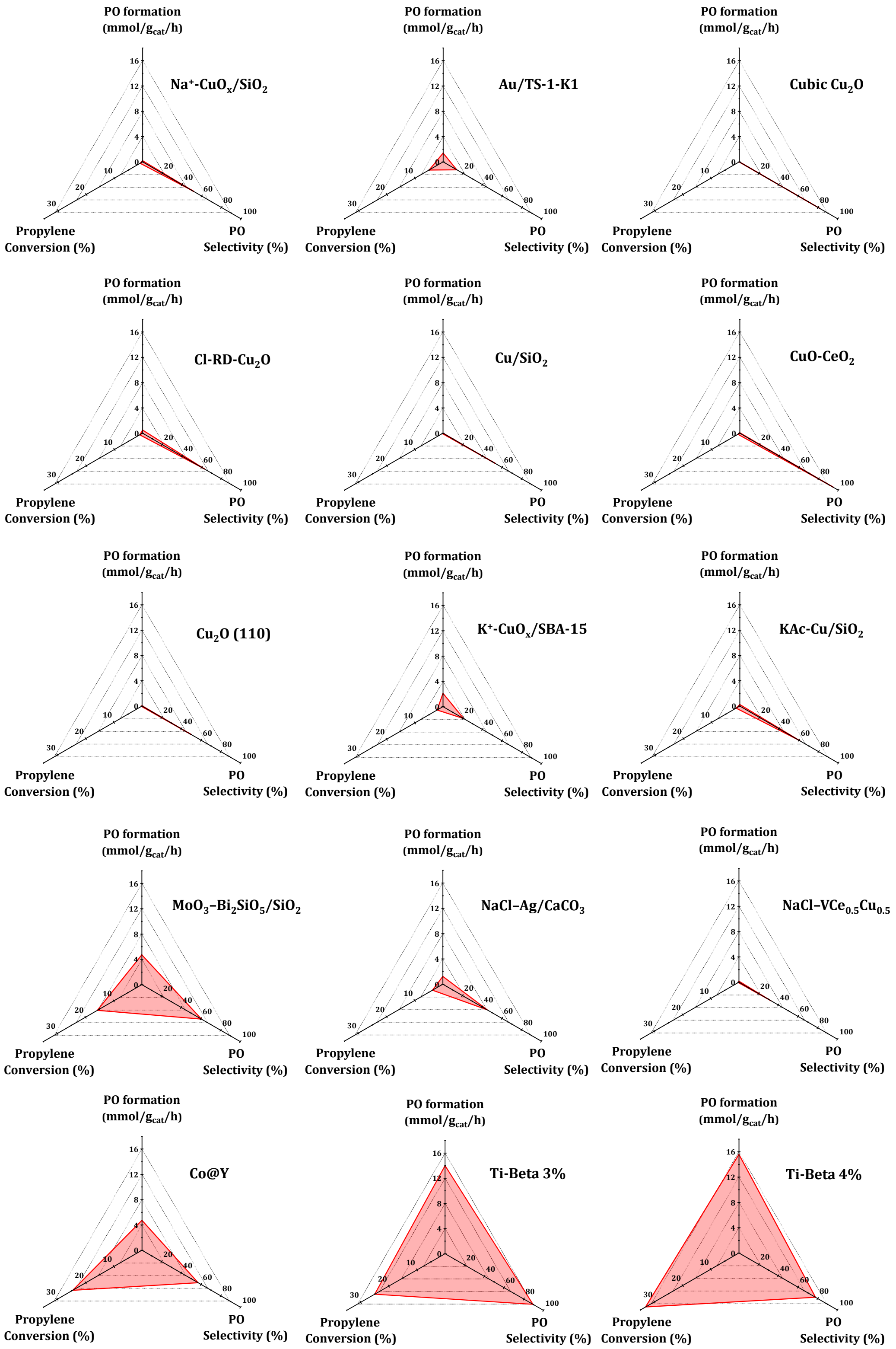

Supplementary Figure 18| Comparison of various catalysts for propylene aerobic epoxidation in terms of propylene conversion, PO selectivity and PO formation rate. Detailed information available in **Supplementary Table 3**.

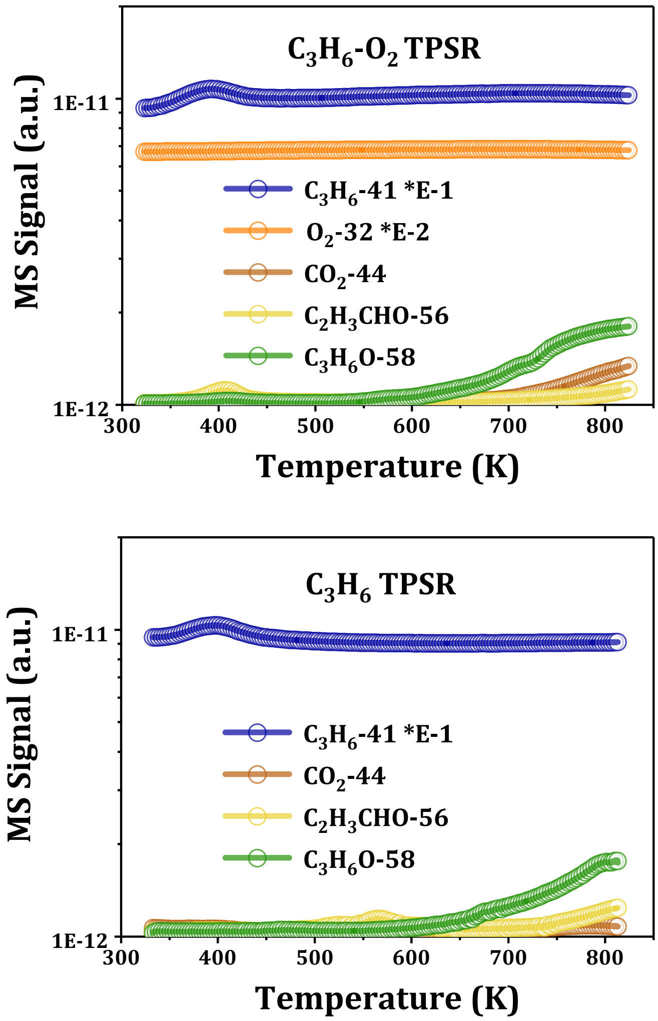

Supplementary Figure 19| TPSR profiles of C_3H_6-O_2 and C_3H_6 on Ti-Beta-3% catalyst. Reaction conditions: 0.1 g catalyst, 5%C_3H_6-(0 or 5%)O_2-(95 or 90%)He, 60 mL/min.

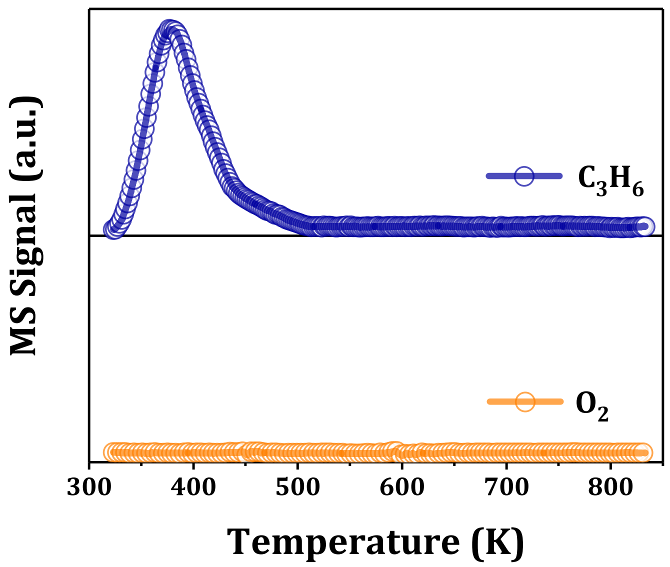

Supplementary Figure 20| TPD profiles of O_2 and C_3H_6 on Ti-Beta-3%.

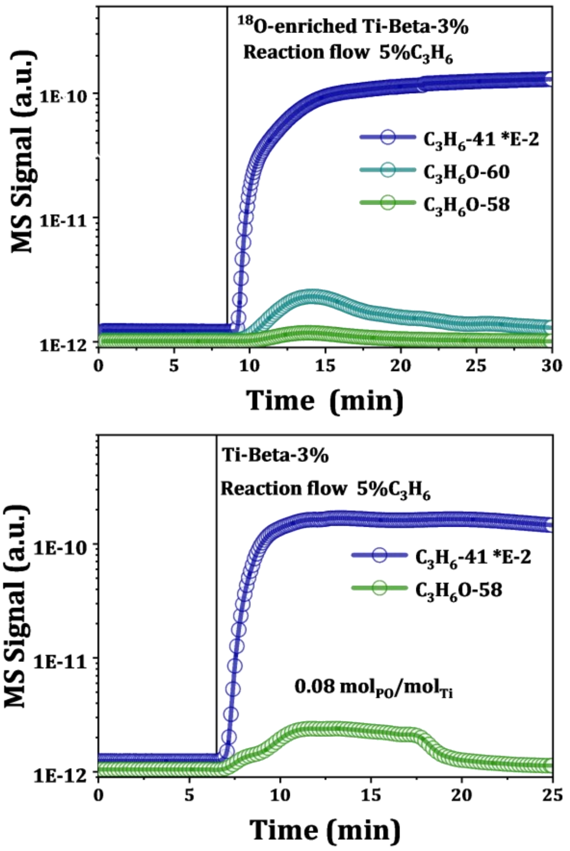

Supplementary Figure 21| TKA results of Ti-Beta catalysts. After activation and He purging of 0.1 g ^{18}O-enriched Ti-Beta-3% catalyst, the stream was switched from 60 mL/min He to 60 mL/min 5%C_3H_6-95%He, at 8 min at 773; After activation and He purging of 0.1 g Ti-Beta-3% catalyst, the stream was switched from 60 mL/min He to 60 mL/min 5%C_3H_6-95%He, at 6 min at 773 K.

Note: The fresh Ti-Beta sample was pretreated in vacuum (<10^{-2} Pa) at 673 K for 12 h, followed by thermal treatment in the atmosphere of 500 mbar $^{18}O_2$ at 873 K for 20 h to derive the ^{18}O-enriched Ti-Beta-3% sample[59].

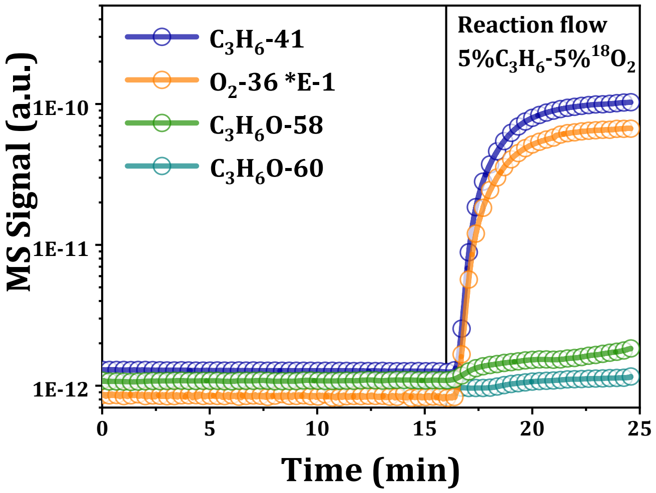

Supplementary Figure 22| TKA results of Ti-Beta catalyst. After activation and He purging of 0.1 g Ti-Beta catalyst, the stream was switched from 60 mL/min He to 60 mL/min 5%C_3H_6-5%$^{18}O_2$-90%He, at 16 min at 673 K.

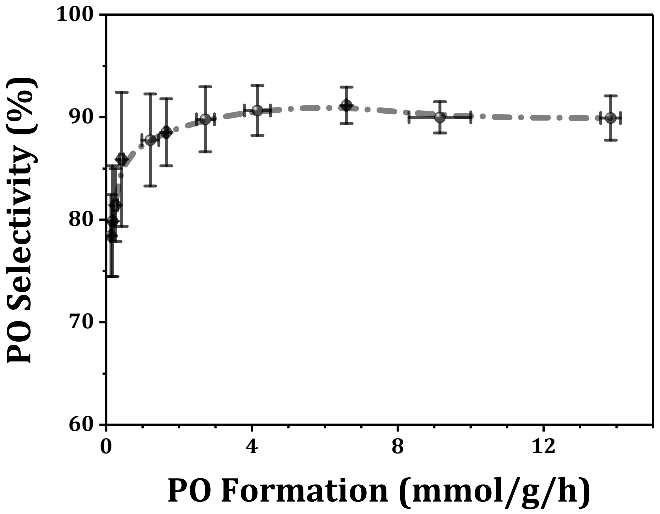

Supplementary Figure 23| Selectivity to PO as a function of PO formation rate over Ti-Beta-3% catalyst.

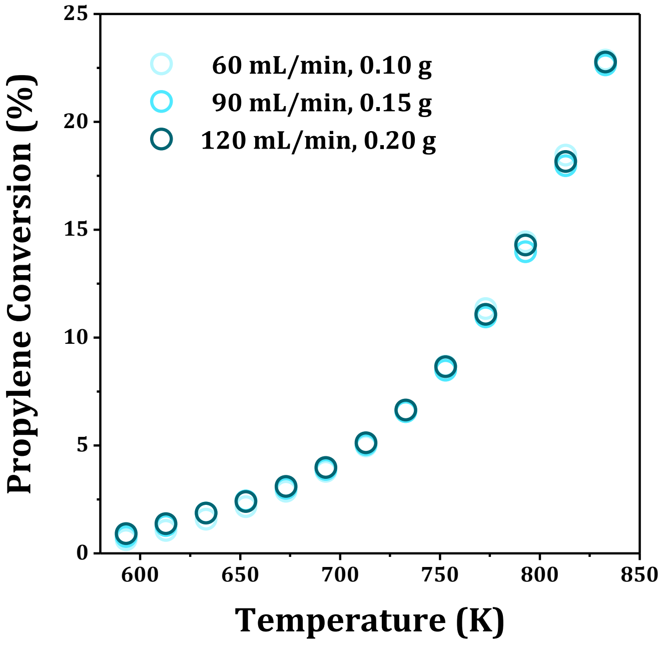

Supplementary Figure 24| Diagnostic tests for propylene aerobic epoxidation over Ti-Beta-3% catalyst.

Note: The overall flow of reaction feed (5%C_3H_6-5%O_2-95%He) was controlled at 60-120 mL/min, and the weight of catalyst loaded in the reactor was adjusted to keep the constant GHSV of 36000 mL/g/h. The catalyst was pretreated in 20%O_2/He at 573 K for 1 h, and then the reaction mixture of 5%C_3H_6-5%O_2 in He was fed to the catalyst. The temperature was ramped up at a rate of 2 K/min.

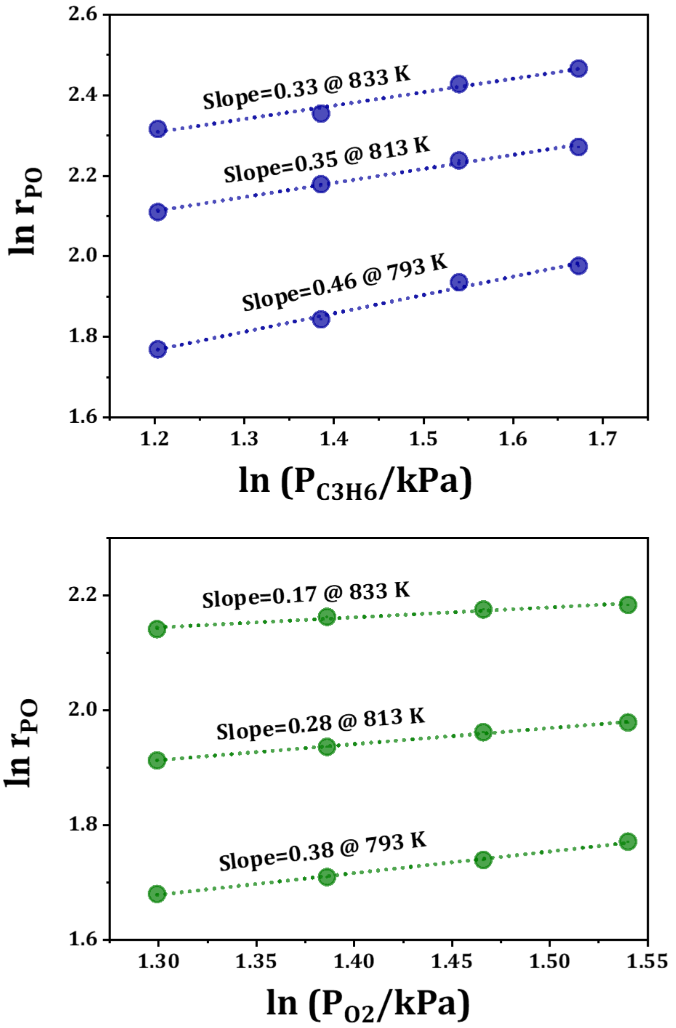

Supplementary Figure 25| Reaction rates of PO formation as a function of C_3H_6 or O_2 concentrations catalyzed by Ti-Beta-3% at 793, 813 and 833 K. Reaction order determined by the slope of each line.

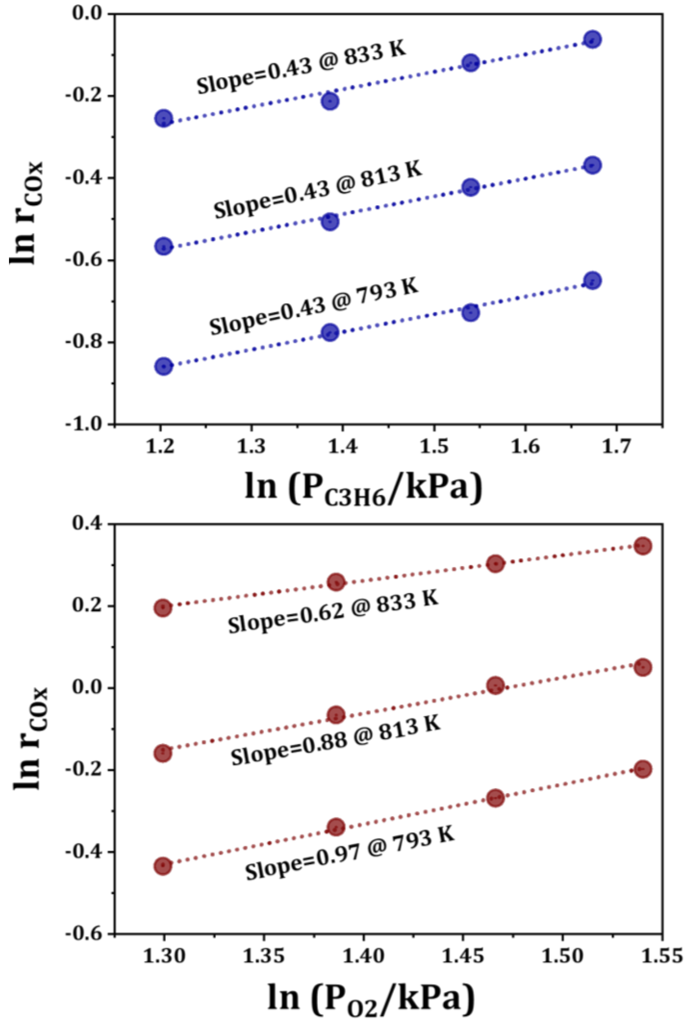

Supplementary Figure 26| Reaction rates of CO_x formation as a function of C_3H_6 or O_2 concentrations catalyzed by Ti-Beta-3% at 793, 813 and 833 K. Reaction order determined by the slope of each line.

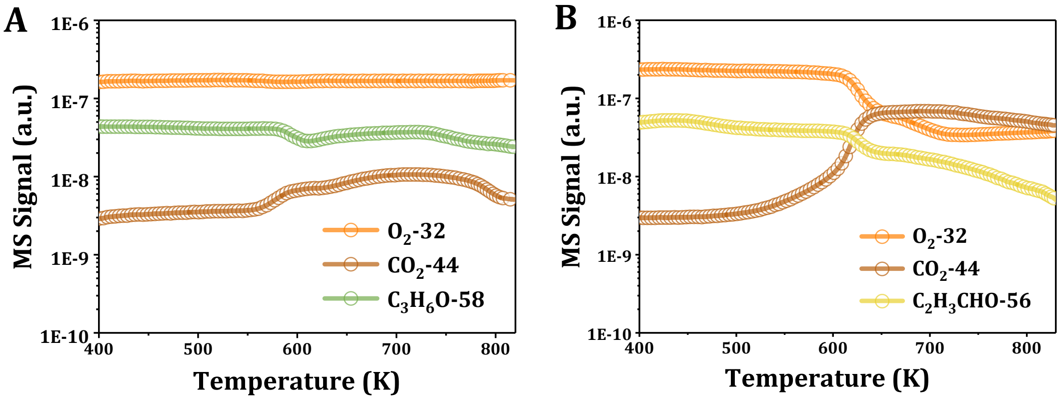

Supplementary Figure 27| TPSR profiles of PO **(A)** and acrolein **(B)** aerobic oxidation over the Ti-Beta-3% catalyst. Reaction conditions: 0.1 g catalyst, 5%PO (or acrolein)-5%O_2-90%He, 60 mL/min.

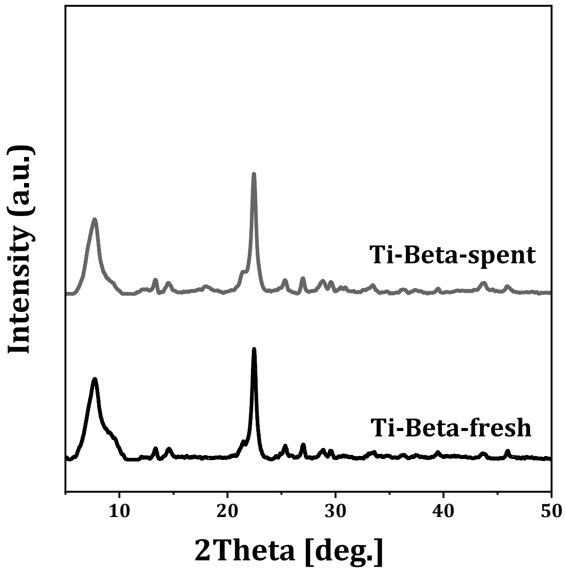

Supplementary Figure 28| XRD patterns of Ti-Beta-3% before and after propylene aerobic epoxidation at 813 K for 240 h.

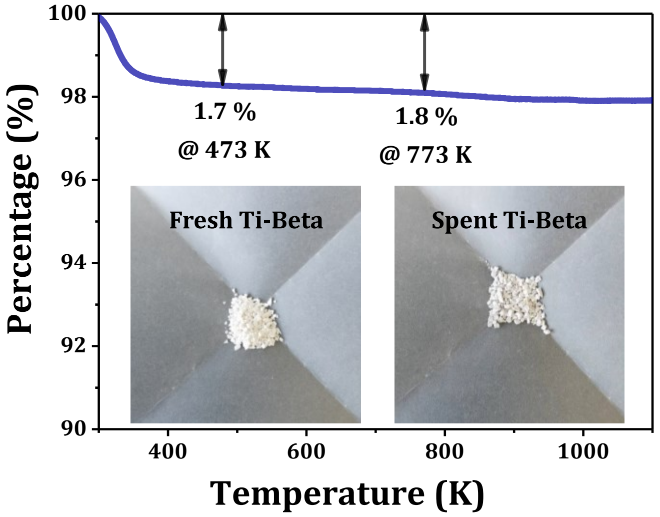

Supplementary Figure 29| Thermogravimetric analysis of Ti-Beta-3% after propylene aerobic epoxidation at 813 K for 240 h. Photographs of fresh and spent catalysts shown inset.

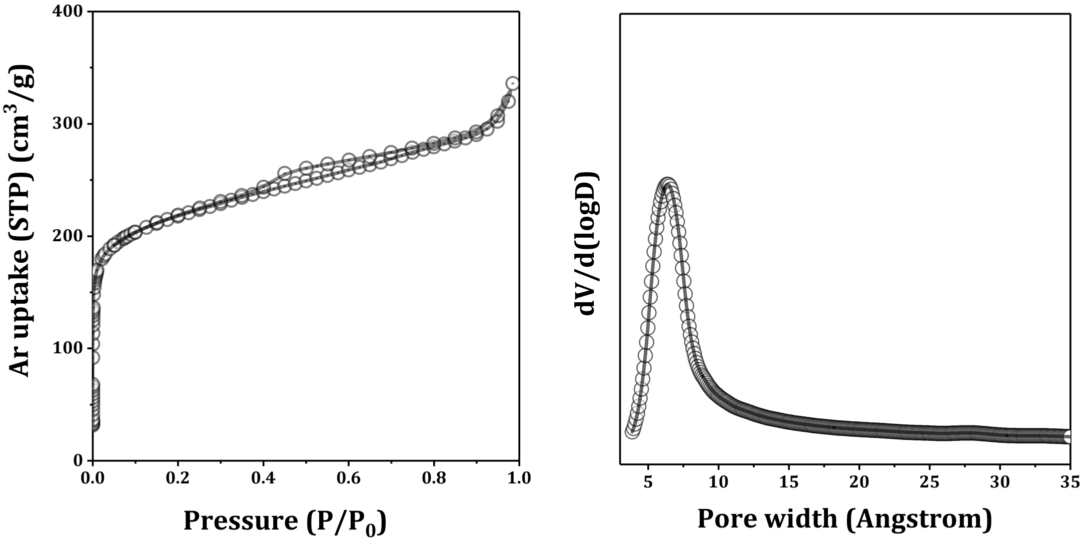

Supplementary Figure 30| Ar sorption isotherms at 87 K and pore width distribution of Ti-Beta-3% after propylene aerobic epoxidation.

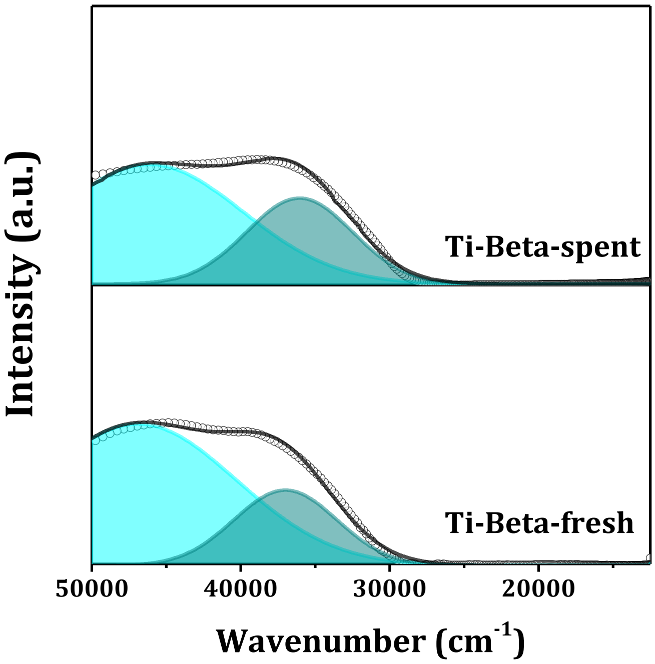

Supplementary Figure 31| UV-vis spectra of Ti-Beta-3% before and after propylene aerobic epoxidation.

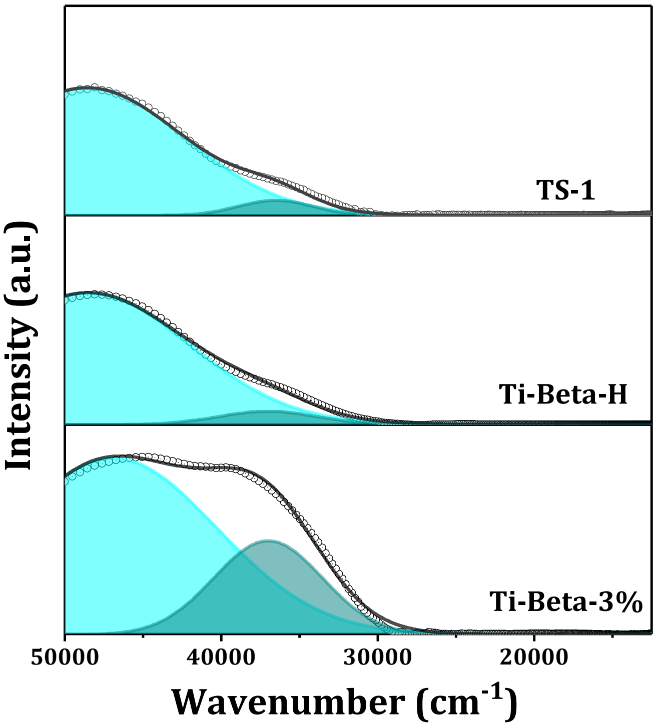

Supplementary Figure 32| UV-vis spectra of selected Ti-containing silicates.

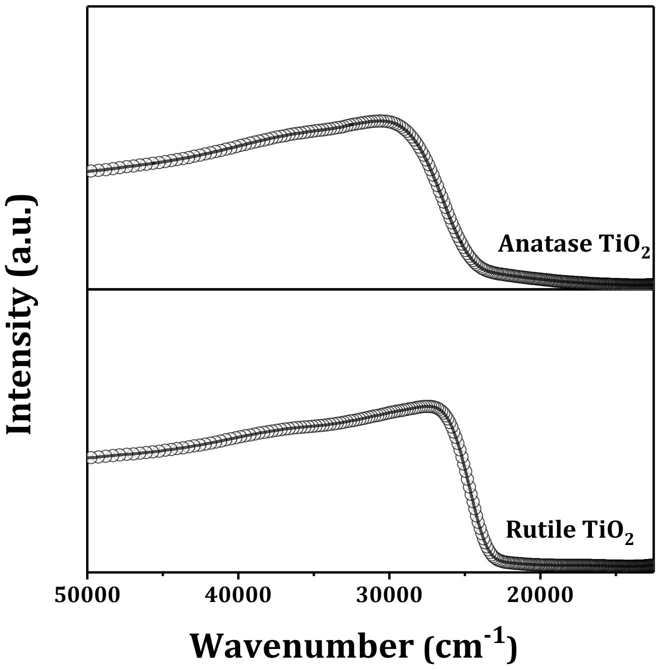

Supplementary Figure 33| UV-vis spectra of anatase and rutile TiO_2 samples.

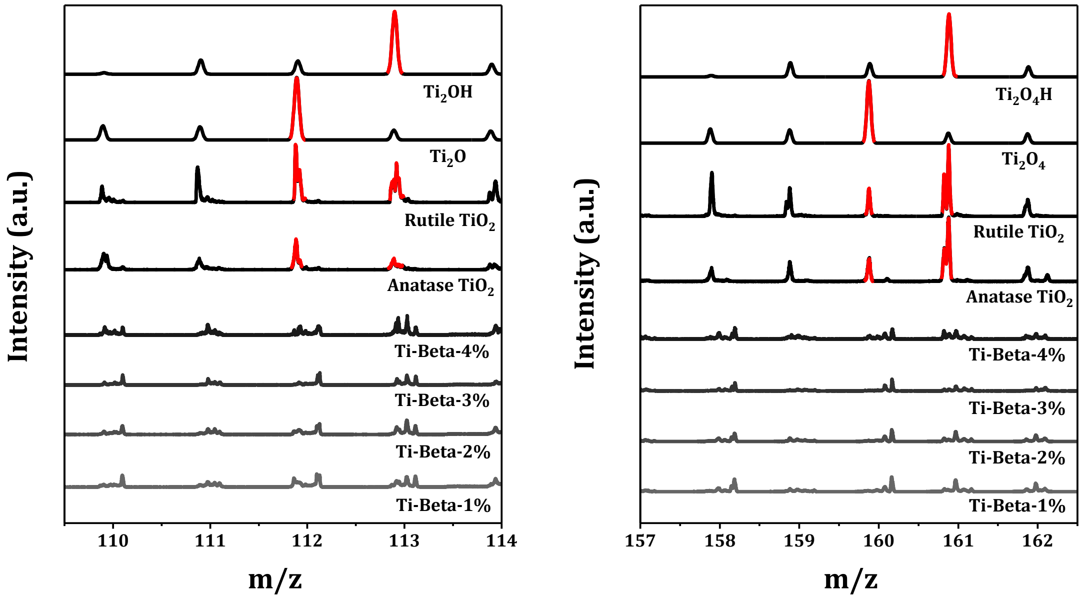

Supplementary Figure 34| TOF-SIMS analyses of dinuclear Ti species in TiO_2 and Ti-Beta samples.

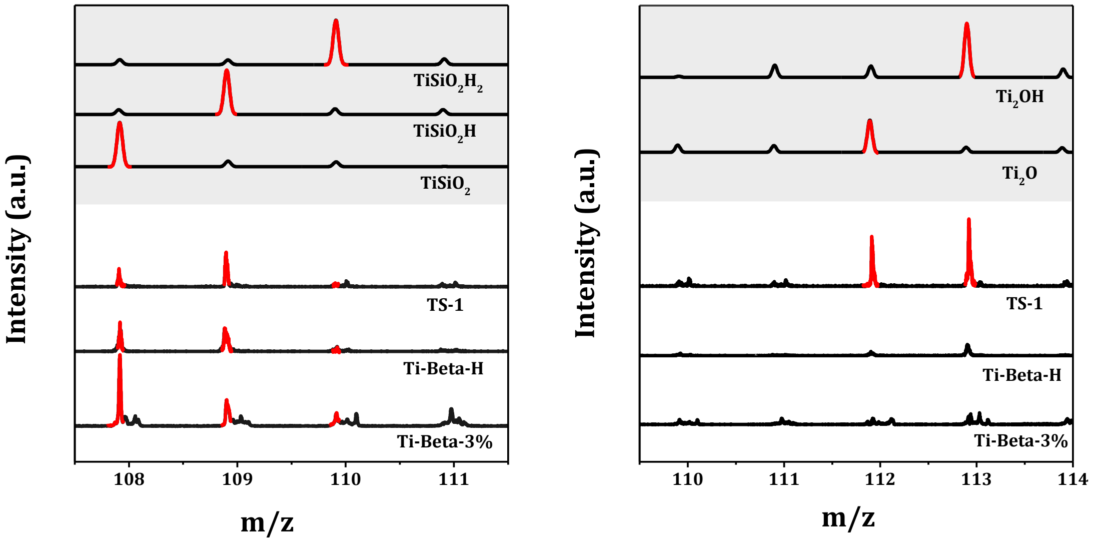

Supplementary Figure 35| TOF-SIMS analyses of selected Ti-containing silicates.

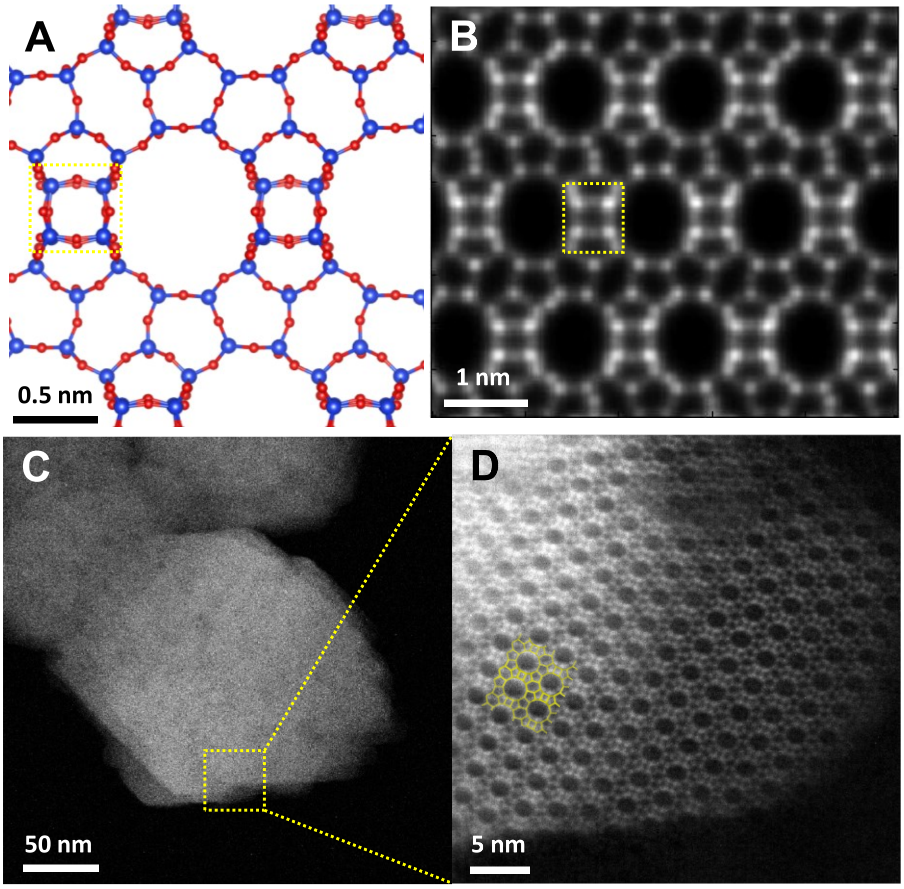

Supplementary Figure 36| Simulated and experimental ADF-STEM images of Si-Beta. (**A**) Illustration of polymorph Beta_A structure; (**B**) Simulated ADF-STEM image of polymorph Beta_A structure. (Thickness: 100.3 Å); (**C**) Experimental ADF-STEM image of Si-Beta; (**D**) Enlarged-view ADF-STEM image (a 2D difference filter was applied) of Si-Beta.

Note: ADF-STEM image of Si-Beta shows slightly brighter contrast in the four-membered rings due to the different amounts of silicon atoms at those positions in the projection.

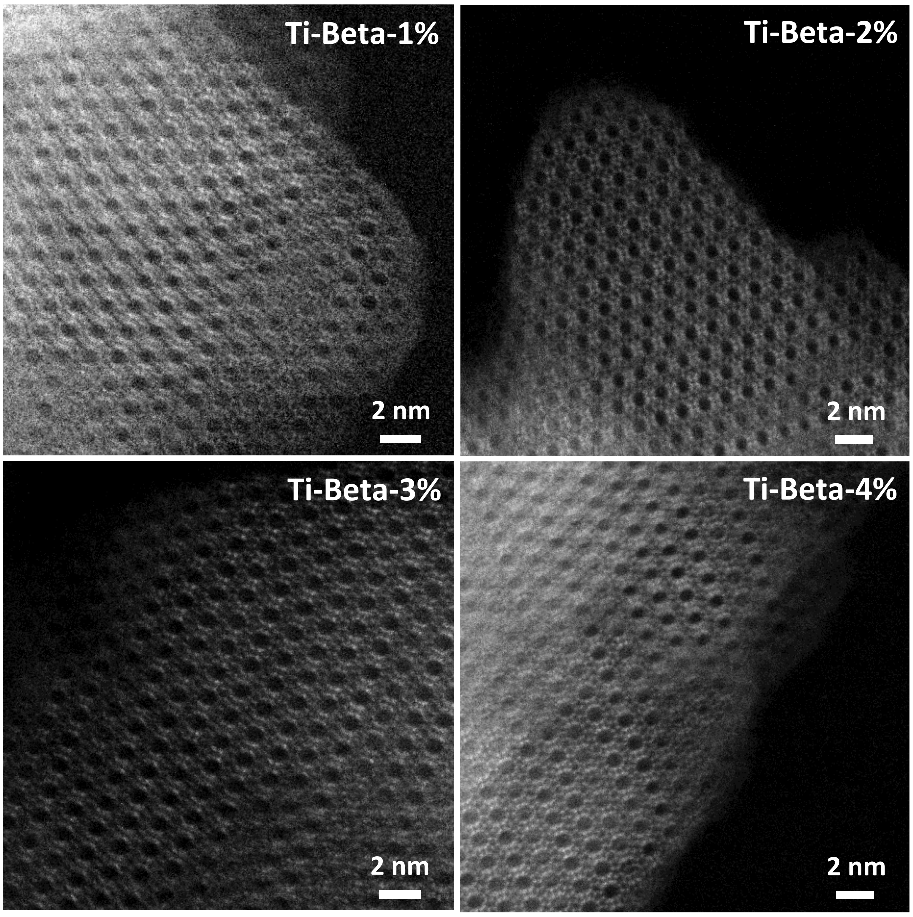

Supplementary Figure 37| *Cs*-corrected ADF-STEM images Ti-Beta samples with different Ti contents.

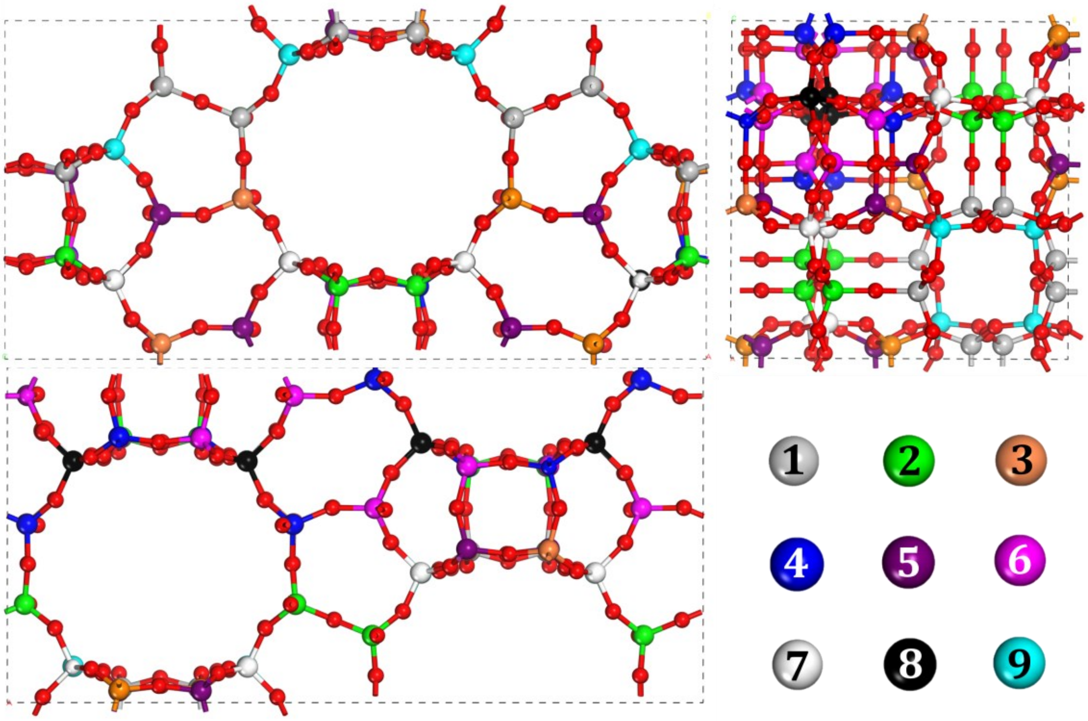

Supplementary Figure 38| Various crystallographic T sites in Beta framework (polymorph A).

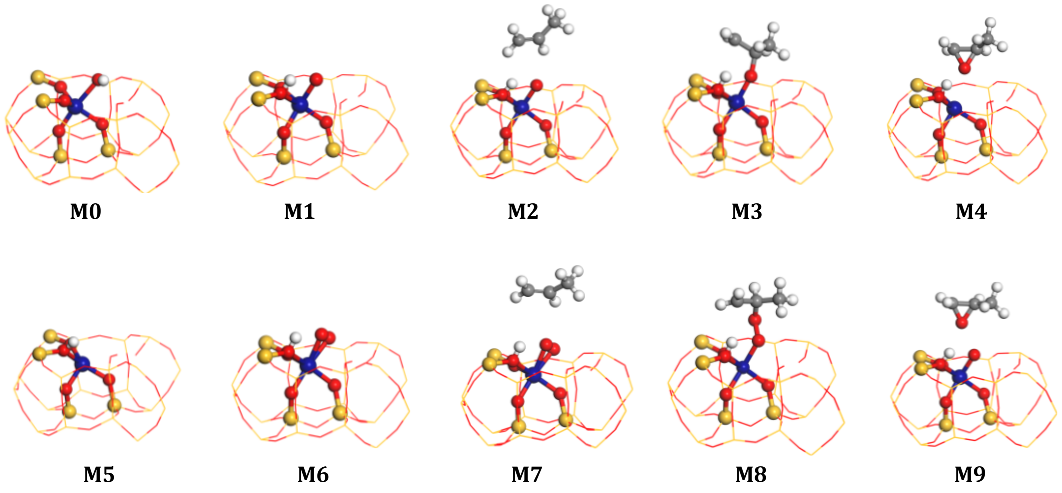

Supplementary Figure 39| Optimized structures involved in propylene oxidation to PO by molecular oxygen over H-terminated pentacoordinated Ti site in Beta zeolite (polymorph A).

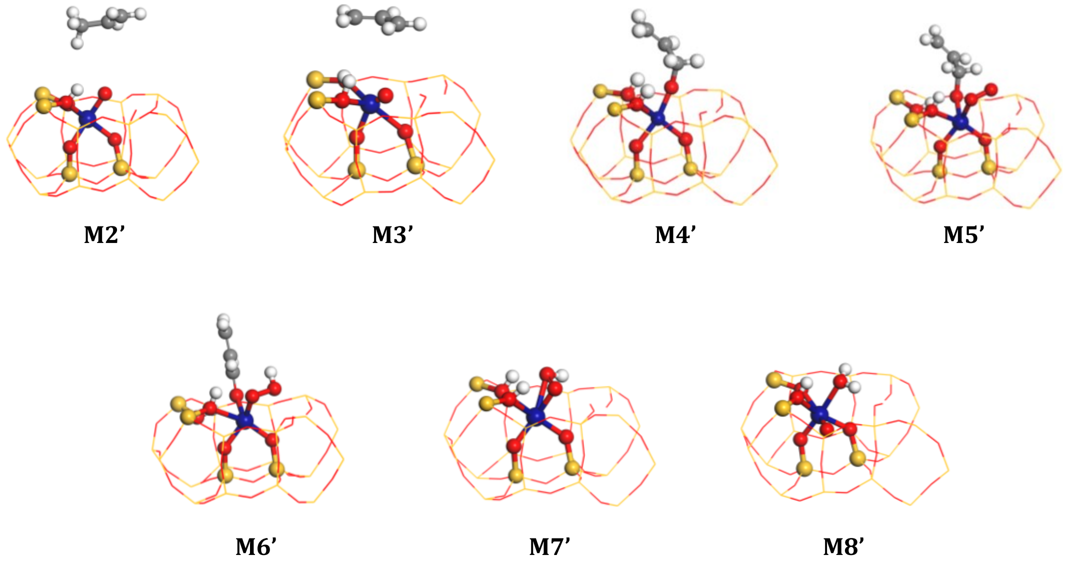

Supplementary Figure 40| Optimized structures involved in propylene oxidation to acrolein by molecular oxygen over H-terminated pentacoordinated Ti site in Beta zeolite (polymorph A).

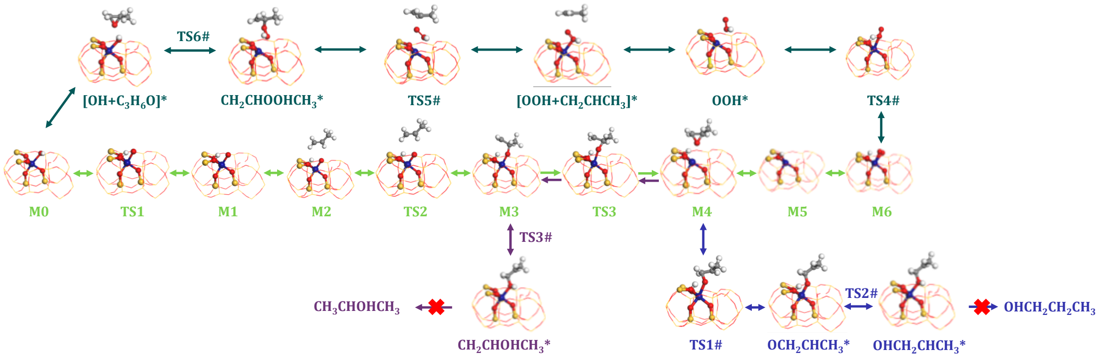

Supplementary Figure 41| Possible competitive reaction pathways competing with the formation of PO.

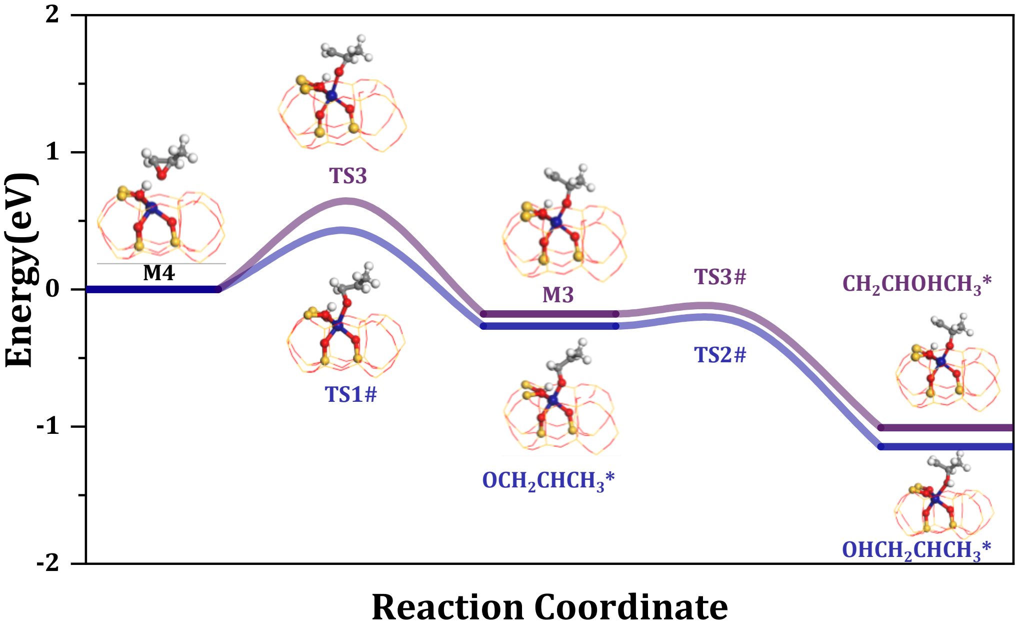

Supplementary Figure 42| Possible pathways competing with the formation of PO and the corresponding energy profiles-I

Note: Intuitively, the **H (proton)** might transfer to the epoxide to compete with epoxide desorption. There are two possible pathways for this competitive reaction, as shown in **Supplementary Fig. 41**. The first pathway is as follows: M4→ OCH_2CHCH_3*→ $OHCH_2CHCH_3$*→$OHCH_2CH_2CH_3$*. The second pathway is as follows: M4 →M3 →$CH_2CHOHCH_3$* → $CH_3CHOHCH_3$*.

For the first pathway shown in the navy line in **Supplementary Fig. 41**, started from M4 **(the intermediate in Supplementary Fig. 422)**, considering the attack of proton toward epoxide (C_3H_6O), the C_3H_6O molecule should adsorb at the Ti site firstly to form OCH_2CHCH_3* *via* TS1# (E_a=0.43 eV). Then, the proton transfers to the O atom of OCH_2CHCH_3* to form $OHCH_2CHCH_3$* without energy barrier. Alternatively, for the second pathway shown in the violet line in **Supplementary Fig. 42**, the C_3H_6O molecule adsorbs at the Ti site to form CH_2CHOCH_3*(M3) *via* TS3 (E_a=0.64 eV), *i.e.*, M4 back to M3, and then the proton transfers to the O atom of the CH_2CHOCH_3* to form $CH_2CHOHCH_3$* without energy barrier. Although the formation of $OHCH_2CHCH_3$* and $CH_2CHOHCH_3$* is feasible, these two pathways cannot be achieved due to the lack of extra H atoms provided for these two intermediates to form the corresponding propanol ($OHCH_2CH_2CH_3$* and $CH_2CH_2OHCH_3$*) in the oxidizing reaction environment. The reverse processes of these two pathways are energetically feasible, resulting in the formation of C_3H_6O from the $OHCH_2CHCH_3$* and $CH_2CHOHCH_3$*.

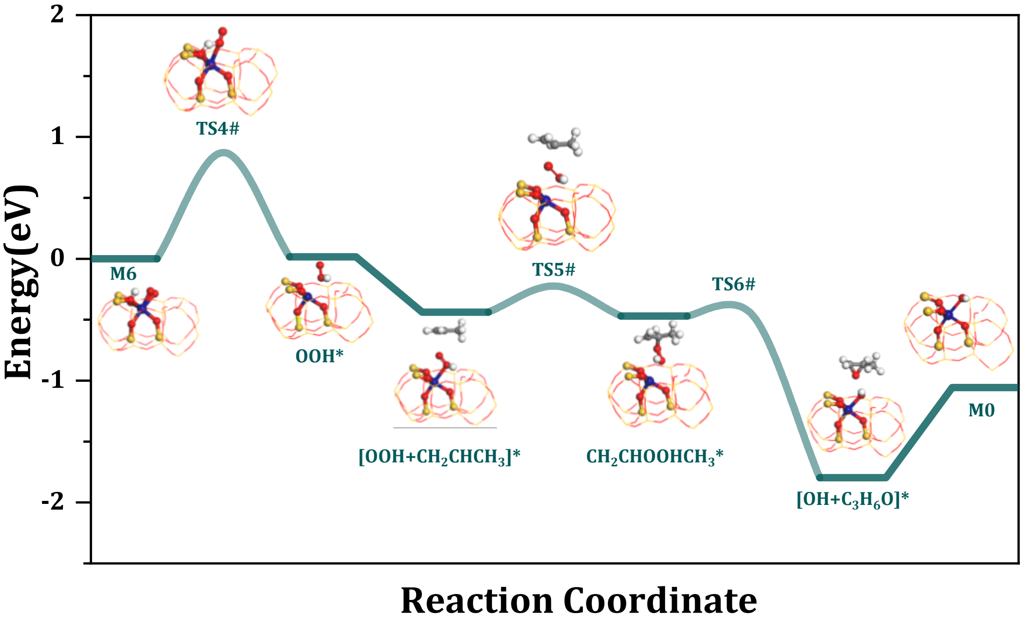

Supplementary Figure 43| Possible pathways competing with the formation of PO and the corresponding energy profiles-II

Note: For the main reaction pathway from M0 to M9 (**Supplementary Fig. 39**), there might exist a competitive reaction caused by the transfer of the **H (proton)** to the OO* as shown in dark green line in **Supplementary Fig. 41**. The detailed pathway is as follows: M6 → OOH*→ [OOH+CH_2CHCH_3]* → $CH_2CHOOHCH_3$ →[OH+C_3H_6O]* →M0+C_3H_6O. The corresponding energy profile is shown in **Supplementary Fig. 43**.

Started from M6 in **Supplementary Fig. 43**, the transfer of proton at the framework O atom next to Ti site to the adsorbed OO* to form OOH* *via* TS4# needs to overcome an energy barrier of 0.87 eV. The formed OOH* may react with a CH_2CHCH_3* intermediate to form $CH_2CHOOHCH_3$* *via* TS5# (E_a=0.21 eV) or desorb from the Ti site (E_{des}=0.46 eV). For the former situation, the O-O bond in OOH* in the $CH_2CHOOHCH_3$* breaks to form OH* adsorbing at the Ti site and C_3H_6O* above the OH* (E_a=0.00 eV) with the active site recovering to the origin M0. Essentially, this pathway is identical to the pathway from M6 to M9 in **Supplementary Fig. 39** that has already taken into account. For the later situation, after the OOH* desorption from Ti site, an another O_2 molecule will adsorb at the Ti site to react with a CH_2CHCH_3* intermediate, also following the pathway from M6 to M9 shown in **Supplementary Fig. 39**. Therefore, the pathway shown in **Supplementary Fig. 39** is already complete, and the step of the proton transfer to the adsorbed OO* and the relevant pathways do not need to be considered.

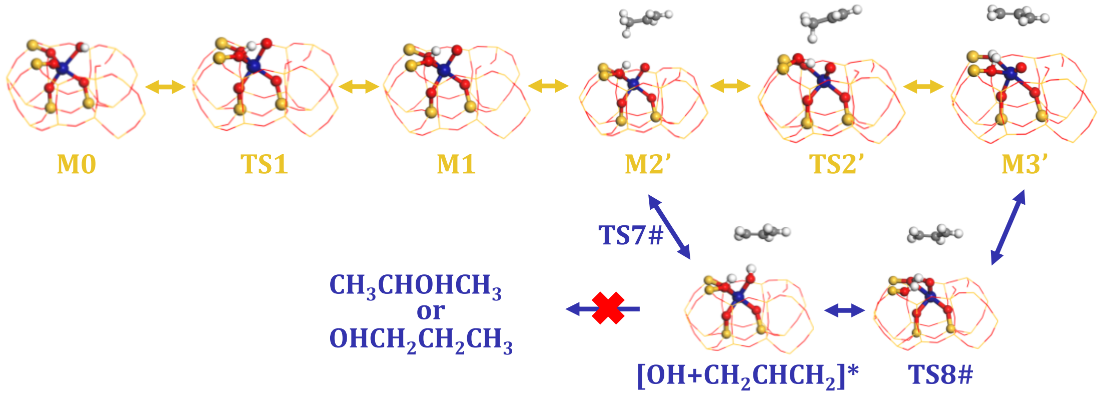

Supplementary Figure 44| Possible competitive reaction pathways competing with the formation of acrolein.

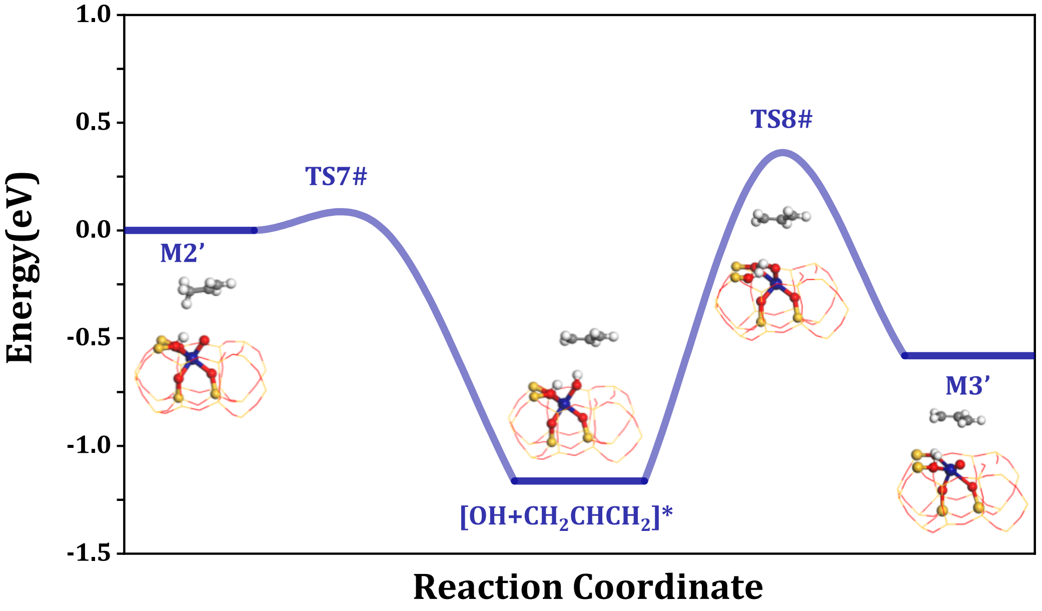

Supplementary Figure 45| Possible pathways competing with the formation of acrolein and the corresponding energy profiles.

Note: For the side reaction pathway from M2’ to M8’ (propylene oxidation to acrolein, **Supplementary Fig. 40**), there might exist a competitive reaction caused by the transfer of the H atom of -CH_3 in the CH_2CHCH_3* to the O* at the Ti site (the non-coordinated O atom), as shown in the blued line in **Supplementary Fig. 44**. The detailed pathway is as follows: M2’ →[OH+CH_2CHCH_2]* →propanol ($OHCH_2CH_2CH_3$* and $CH_2CH_2OHCH_3$*) or M3’. The corresponding energy profile is shown in **Supplementary Fig. 45.**

Started from M2’ in **Supplementary Figs. 44 and 45**, the H atom of -CH_3 in the CH_2CHCH_3* transfers to the active O* at the Ti site to form OH* and CH_2CHCH_2* without energy barrier. The attack of OH* toward CH_2CHCH_2* aiming at the propanol ($OHCH_2CH_2CH_3$* or $CH_2CH_2OHCH_3$*) formation is unachievable due to the lack of H atoms in this oxidizing reaction environment. Thus, the proton of the formed OH* must transfer to the adjacent framework O atom, producing active O* atom to react with the CH_2CHCH_3* (M3’). This proton transfer needs to overcome a high energy barrier of 1.58 eV *via* TS8#, which is higher than the energy barrier (E_a=0.92 eV, **Fig. 3**) of the H transfer from the -CH_3 in the CH_2CHCH_3* directly to the framework O atom next the active O* at the Ti site (the chosen O atom), *i.e.*, the step of M2’ directly to M3’. Therefore, although H from the -CH_3 in the CH_2CHCH_3* may transfer to the non-coordinated O atom (the active O*) than the chosen one, the subsequent reaction processes of the propanol formation are infeasible.

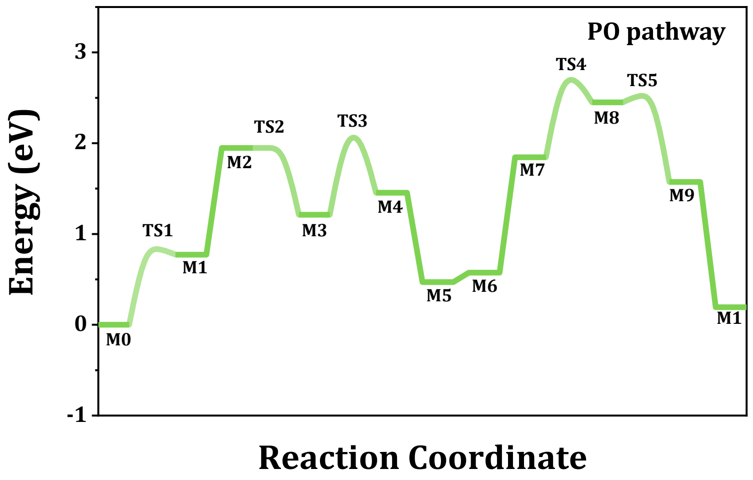

Supplementary Figure 46| Calculated energy profile of propylene oxidation to PO by molecular oxygen over H-terminated pentacoordinated Ti site in Beta zeolite at 813 K (polymorph A).

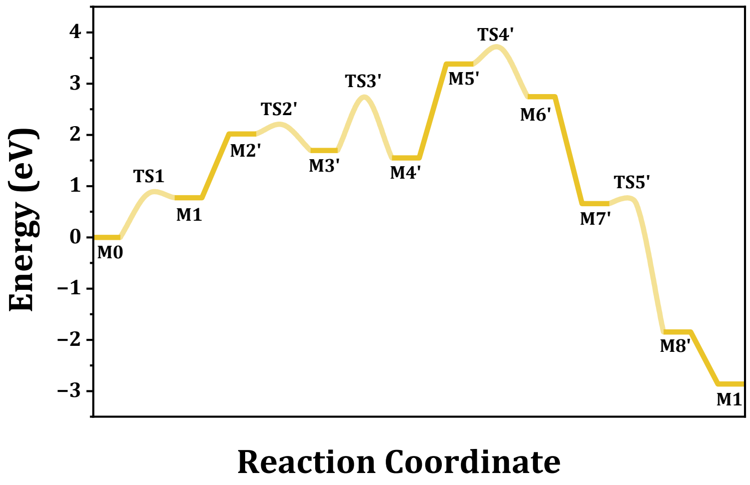

Supplementary Figure 47| Calculated energy profile of propylene oxidation to acrolein by molecular oxygen over H-terminated pentacoordinated Ti site in Beta zeolite at 813 K (polymorph A).

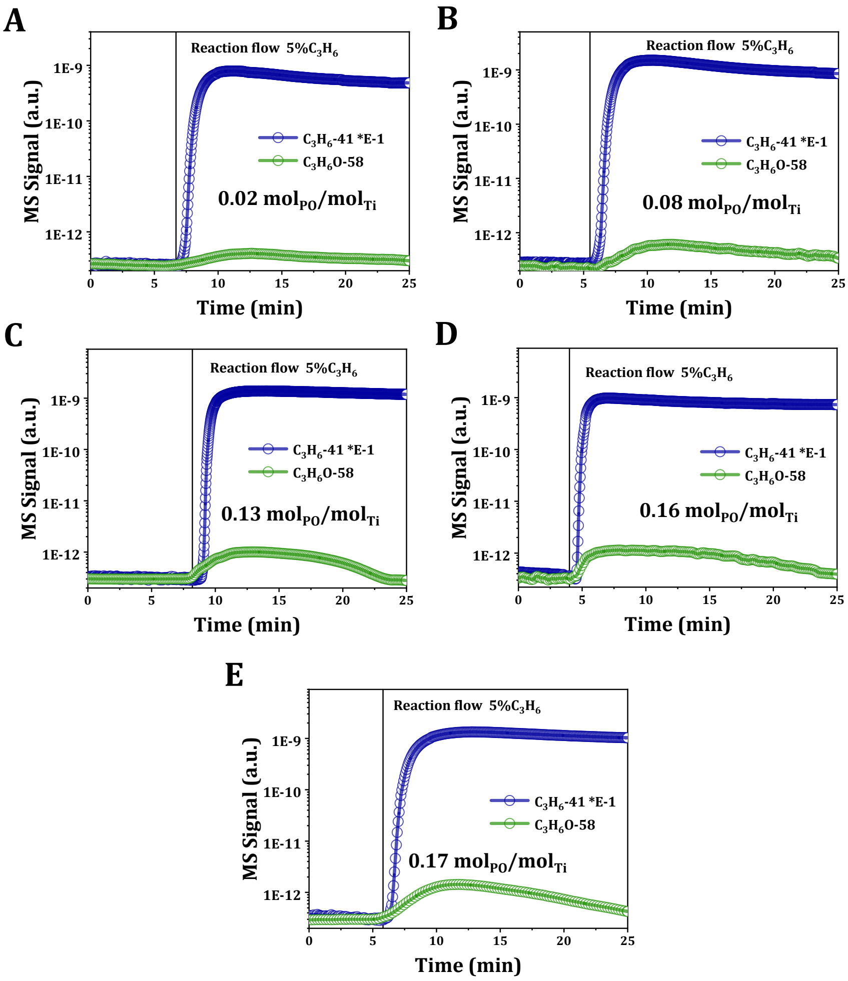

Supplementary Figure 48| TKA results of C_3H_6 surface reaction on O_2-regenerated Ti-Beta-3% catalyst. After activation and He purging, the stream was switched from 60 mL/min He to 60 mL/min 5%C_3H_6-95%He, at 5-10 min at 773 K.

Note: The fresh Ti-Beta-3% sample was subjected to reaction in 5%C_3H_6 flow at 773 K for 1 h to remove all reactive oxygen species. Subsequently, the sample was purged by He purging for 30 min and regenerated in 5% O_2 atmosphere at 373 (**A**), 473 (**B**), 573 (**C**), 673 K (**D**) or 773 K (**E**) for 30 min to derive the O_2-regenerated Ti-Beta-3% catalyst.

In the regenerated step, dioxygen molecule adsorbs on the sample, forming activated oxygen species, which accounted for the production of the second PO molecule when reacting with propylene (the first PO molecule produced from the reaction between propylene and the lattice oxygen). Significantly, the formation of the activated oxygen species from molecular oxygen could be accomplished even at low temperature of 373 K. For the O_2-regenerated Ti-Beta-3% catalyst, a maximum PO formation of ~0.17 mol_{PO}/mol_{Ti} was obtained upon feeding propylene at 773 K, which was about twice that of first step.

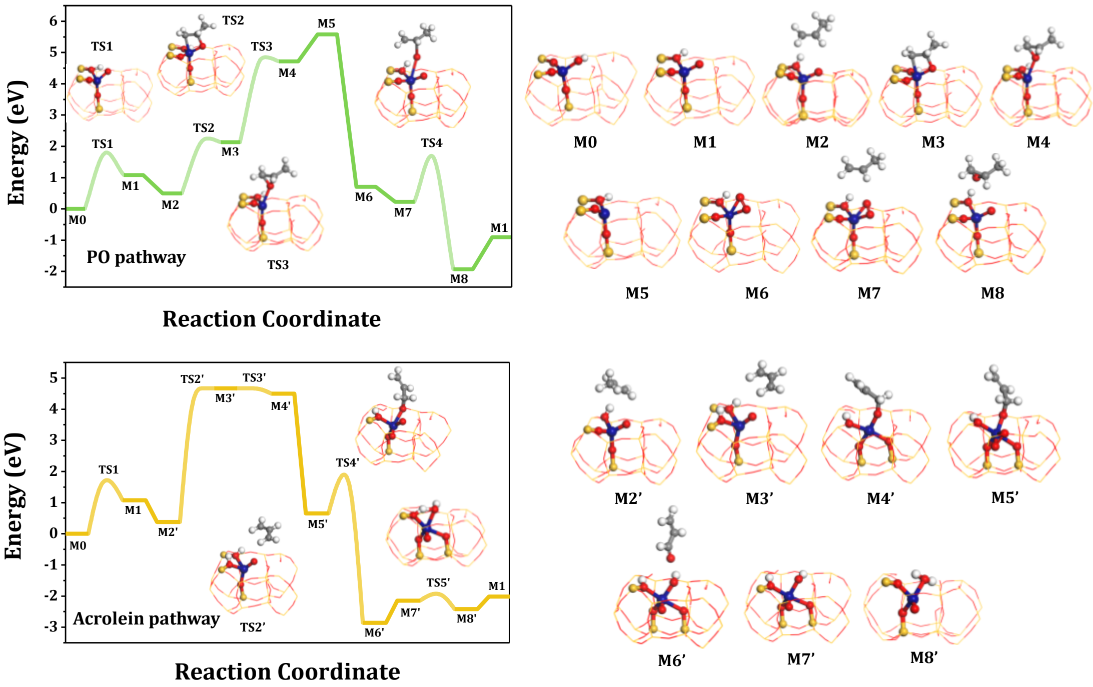

Supplementary Figure 49| Proposed reaction pathways, optimized intermediate structures, transition state structures and the corresponding energy profiles in the formation of PO and acrolein over H-terminated tetracoordinated open Ti site in Beta zeolite at 0 K (polymorph A).

Note: The energy barrier of PO formation over H-terminated tetracoordinated open Ti site in Beta zeolite at 0 K (polymorph A) (**TS3**, E_a= 2.59 eV) is much lower than that of acrolein formation (**TS2'**, E_a= 4.29 eV).

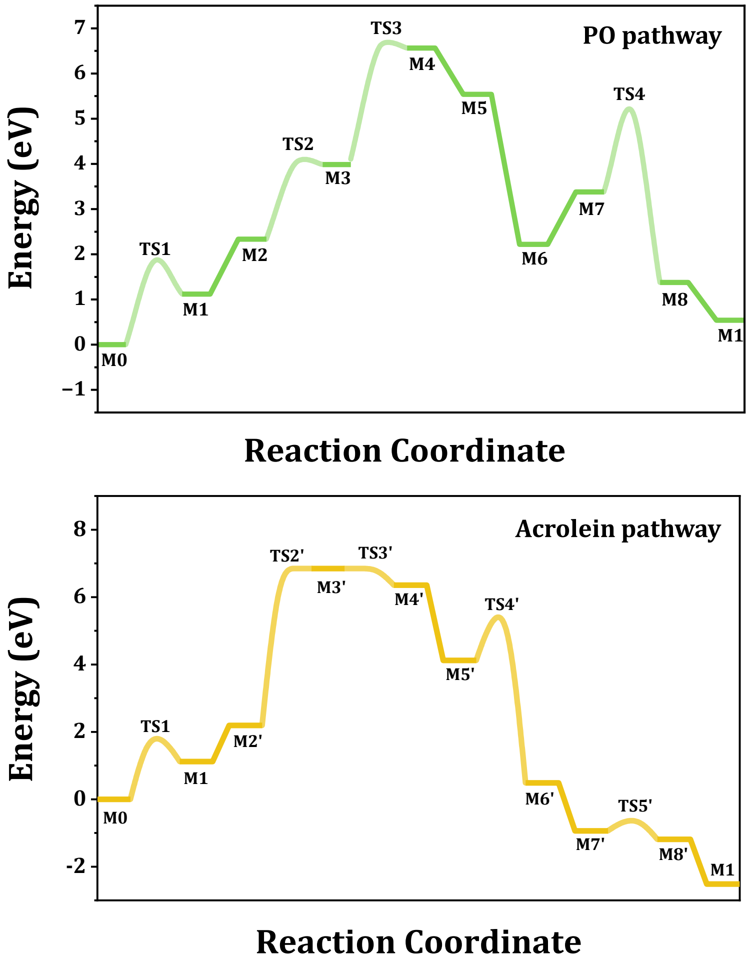

Supplementary Figure 50| Calculated energy profile of propylene oxidation with molecular oxygen over H-terminated tetracoordinated open Ti site in Beta zeolite at 813 K (polymorph A).

Note: The energy barrier of PO formation over H-terminated tetracoordinated open Ti site in Beta zeolite at 813 K (polymorph A) (**TS3**, E_a= 2.58 eV) is much lower than that of acrolein formation (**TS2'**, E_a= 4.66 eV).

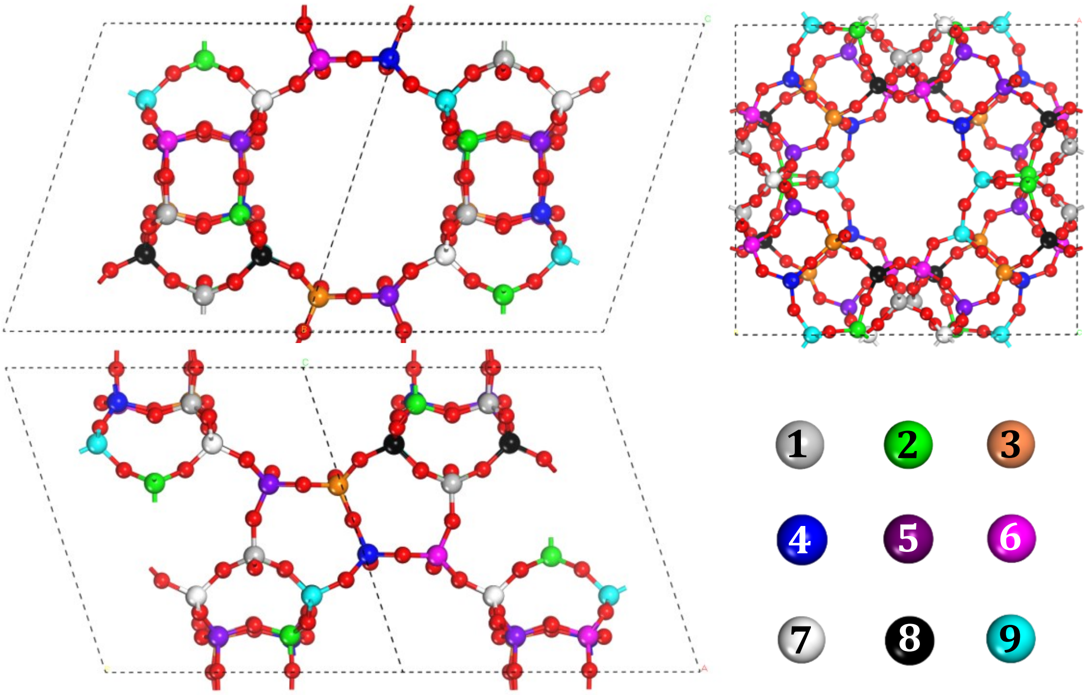

Supplementary Figure 51| Various crystallographic T sites in Beta framework (polymorph B).

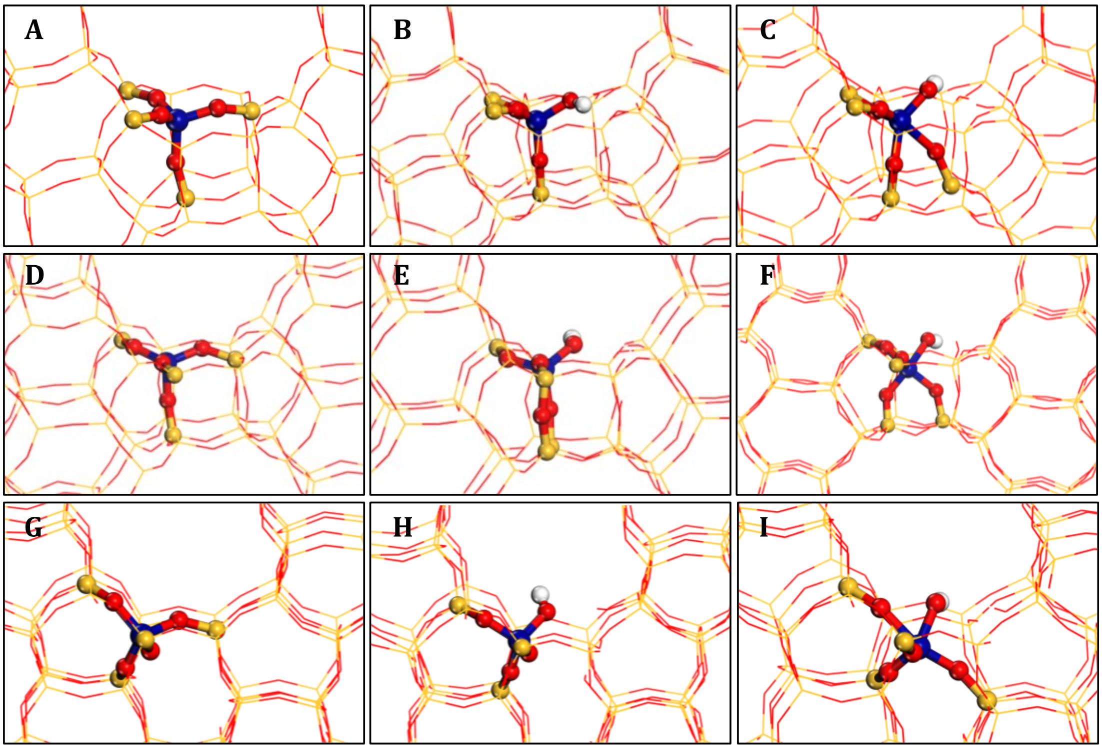

Supplementary Figure 52| Structure models for the tetracoordinated Ti, H-terminated tetracoordinated Ti, and H-terminated pentacoordinated Ti sites in Beta zeolite of polymorph B (A-C) and polymorph C (D-F), as well as MFI zeolite (G-I). Ti: dark blue, O: red, Si: yellow, H: white.

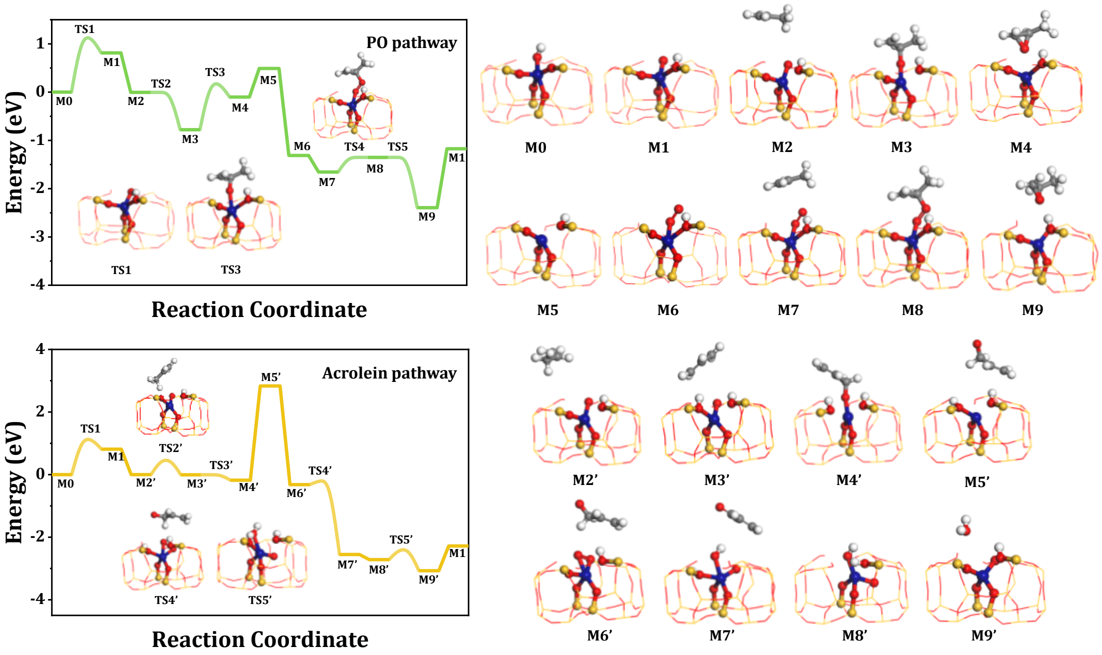

Supplementary Figure 53| Proposed reaction pathways, optimized intermediate structures, transition state structures and the corresponding energy profiles in the formation of PO and acrolein over H-terminated pentacoordinated Ti site in Beta zeolite at 0 K (polymorph B).

Note: The preferential desorption of intermediate in **M4'** is a prerequisite for subsequent steps as the co-adsorption of this intermediate and dioxygen on Ti site is impossible. The desorption energy of this step is as high as 3.01 eV, which renders the occurrence of this step challenging. The energy required for this step can be regarded as an exceedingly high transition state energy barrier (**M4'**→**M5'**) in comparison to that of PO formation (**TS1**, E_a= 1.15 eV).

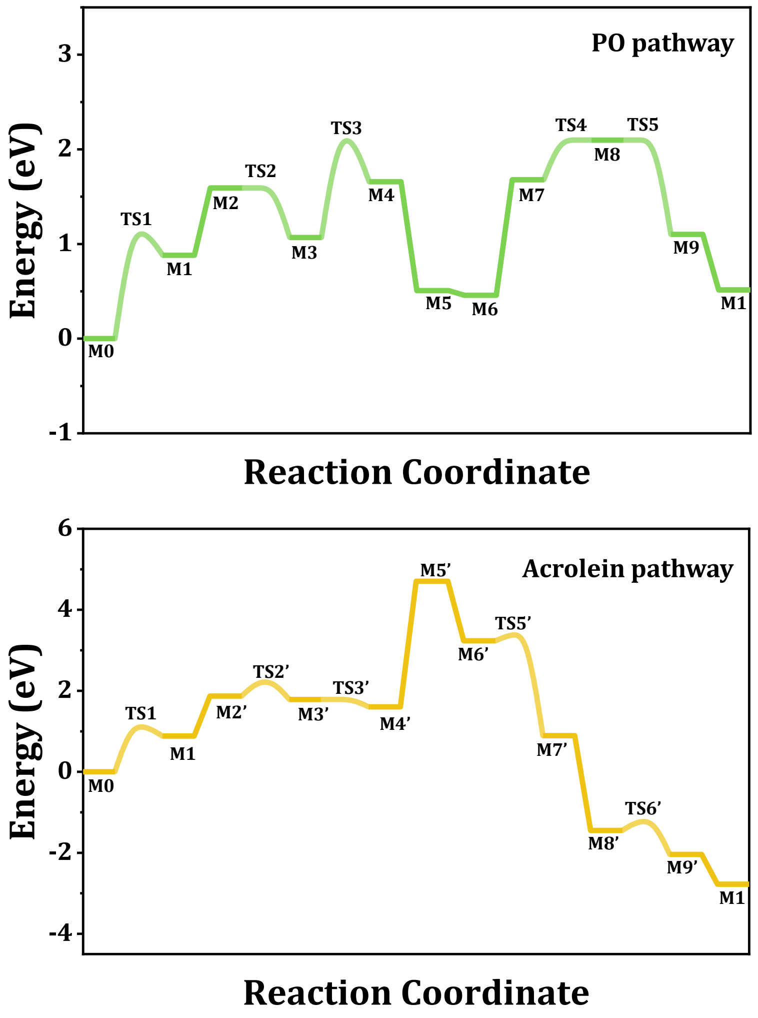

Supplementary Figure 54| Calculated energy profile of propylene oxidation with molecular oxygen over H-terminated pentacoordinated Ti site in Beta zeolite at 813 K (polymorph B).

Note: The preferential desorption of intermediate in **M4'** is a prerequisite for subsequent steps as the co-adsorption of this intermediate and dioxygen on Ti site is impossible. The desorption energy of this step is as high as 3.21 eV, which renders the occurrence of this step challenging. The energy required for this step can be regarded as an exceedingly high transition state energy barrier (**M4'**→**M5'**) in comparison to that of PO formation (**TS1**, E_a= 1.24 eV).

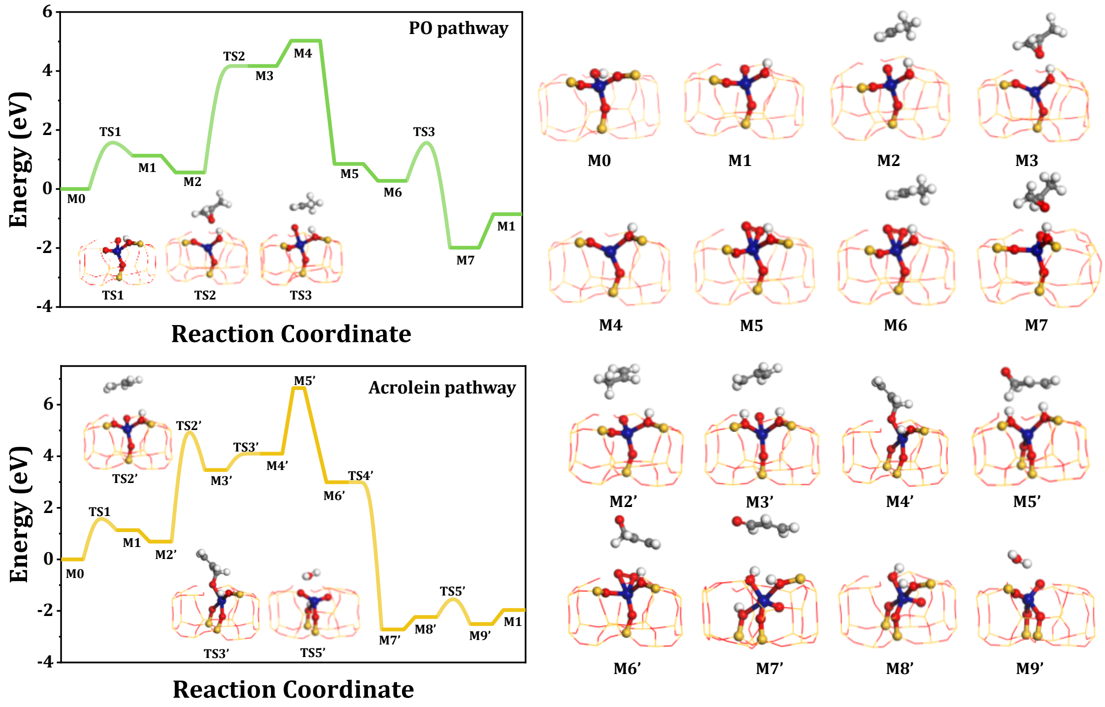

Supplementary Figure 55| Proposed reaction pathways, optimized intermediates structures, transition state structures and the corresponding energy profiles in the formation of PO and acrolein over H-terminated tetracoordinated open Ti site in Beta zeolite at 0 K (polymorph B).

Note: The energy barrier of PO formation over H-terminated tetracoordinated Ti site in Beta zeolite at 0 K (polymorph B) (**TS2**, E_a= 3.61 eV) is lower than that of acrolein formation (**TS2'**, E_a= 4.34 eV). The preferential desorption of intermediate in **M4'** is a prerequisite for subsequent steps as the co-adsorption of this intermediate and dioxygen on Ti site is impossible.

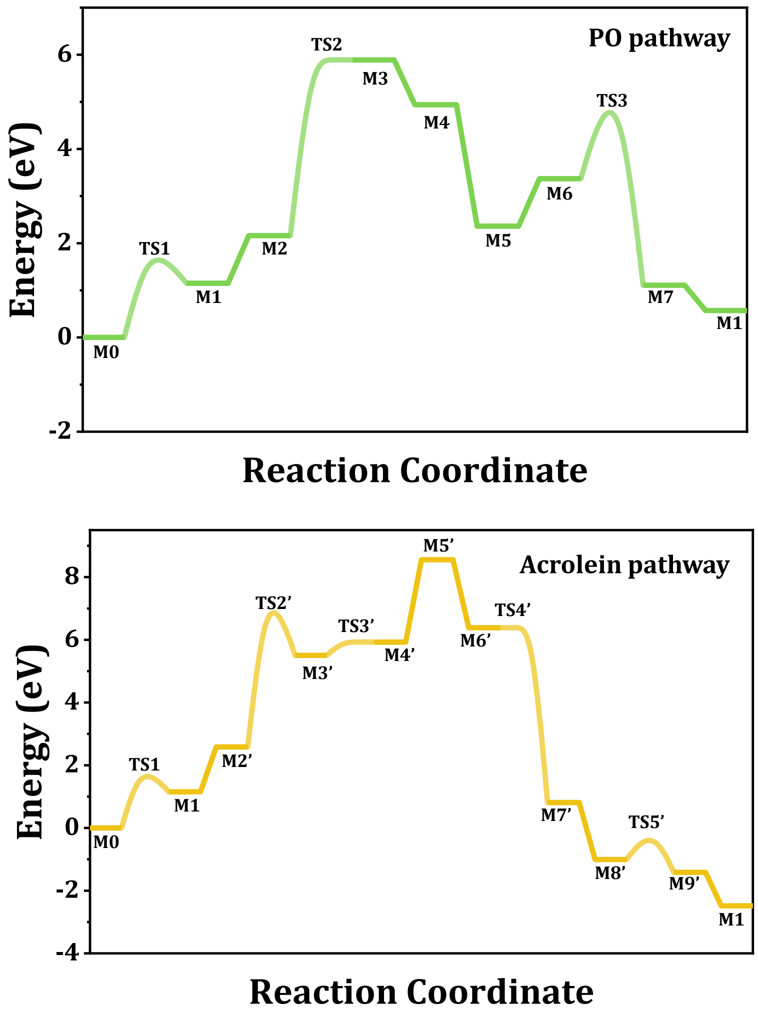

Supplementary Figure 56| Calculated energy profile of propylene oxidation with molecular oxygen over H-terminated tetracoordinated open Ti site in Beta zeolite at 813 K (polymorph B).

Note: The energy barrier of PO formation over H-terminated tetracoordinated Ti site in Beta zeolite at 813 K (polymorph B) (**TS2**, E_a= 3.73 eV) is lower than that of acrolein formation (**TS2'**, E_a= 4.37 eV). The preferential desorption of intermediate in **M4'** is a prerequisite for subsequent steps as the co-adsorption of this intermediate and dioxygen on Ti site is impossible.

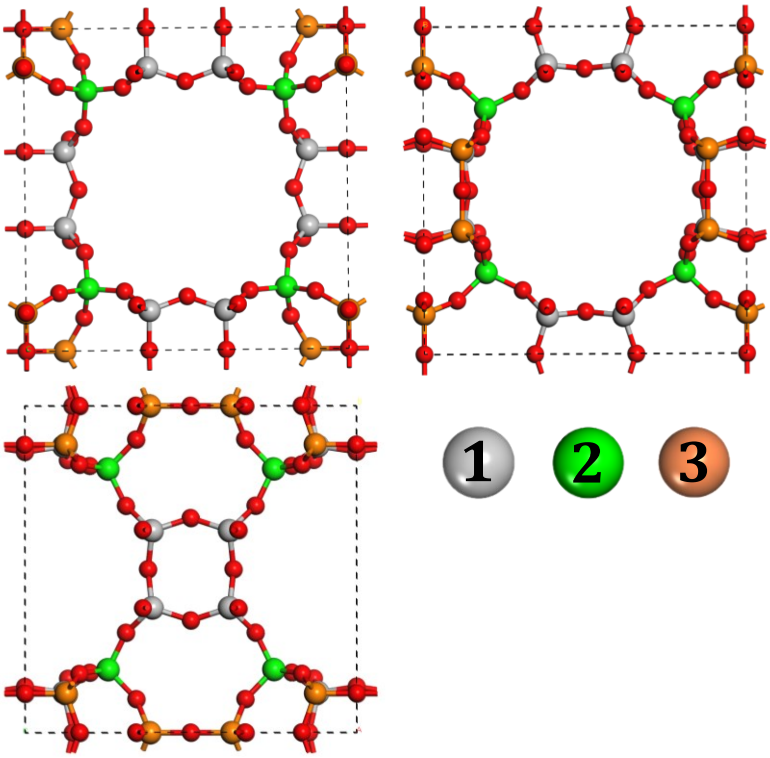

Supplementary Figure 57| Various crystallographic T sites in Beta framework (polymorph BEC).

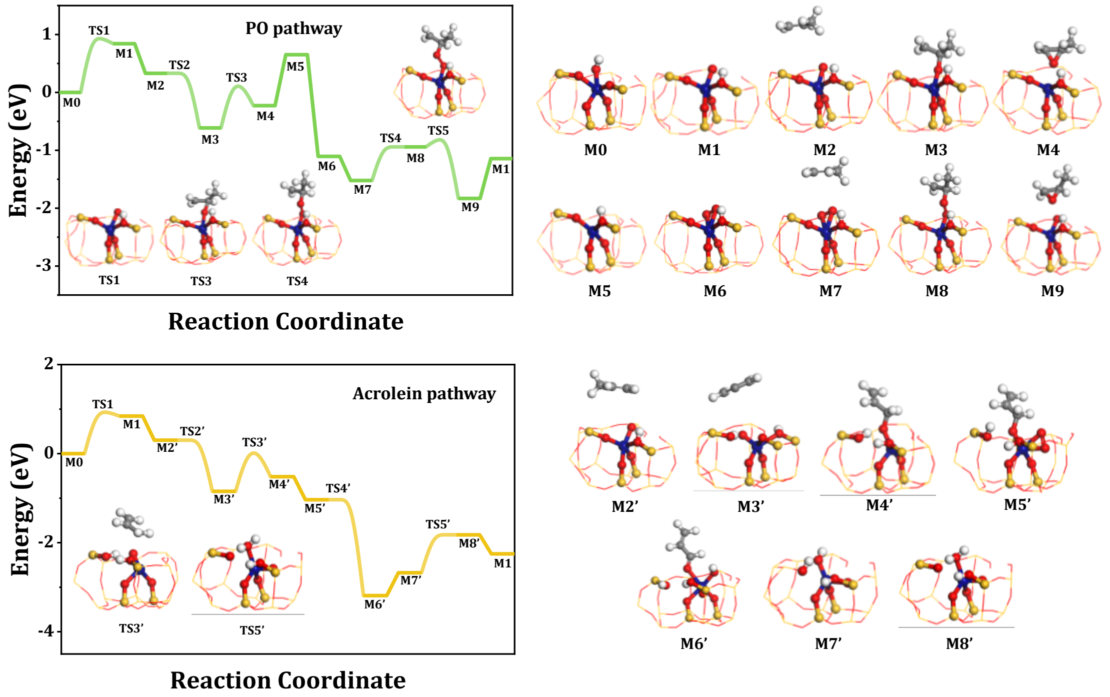

Supplementary Figure 58| Proposed reaction pathways, optimized intermediate structures, transition state structures and the corresponding energy profiles in the formation of PO and acrolein over H-terminated pentacoordinated Ti site in Beta zeolite at 0 K (polymorph C).

Note: The energy barrier of PO formation over H-terminated pentacoordinated Ti site in Beta zeolite at 0 K (polymorph C) (**TS1**, E_a= 0.94 eV; **TS3**, E_a= 0.74 eV) is slightly lower than that of acrolein formation (**TS1**, E_a= 0.94 eV; **TS3'**, E_a= 0.89 eV).

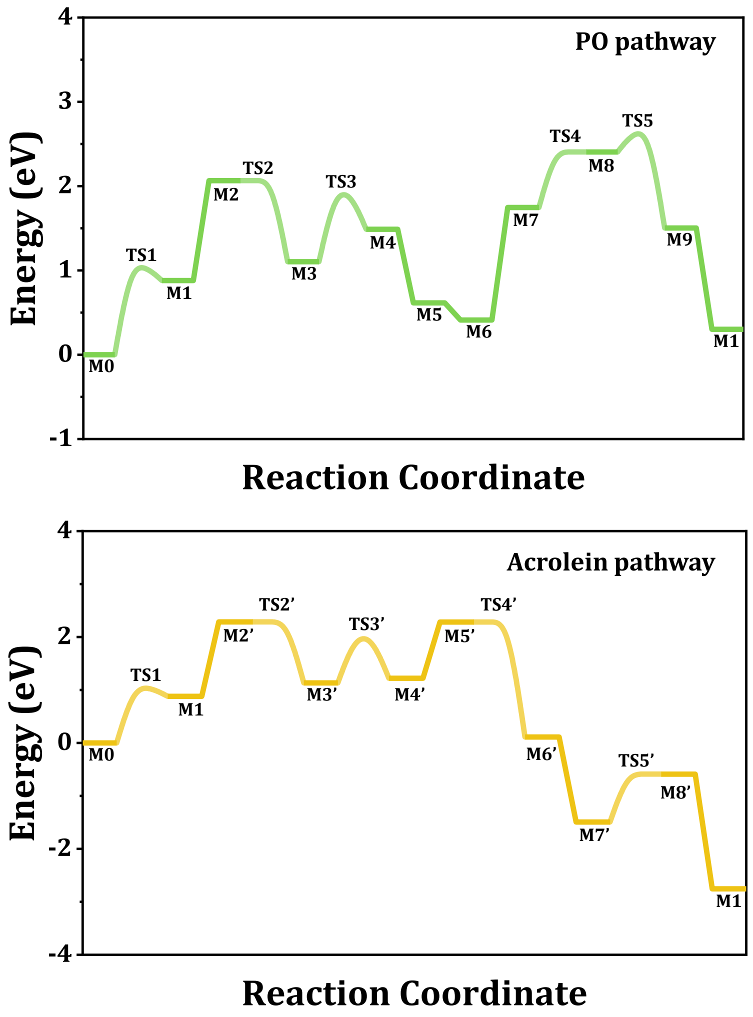

Supplementary Figure 59| Calculated energy profile of propylene oxidation with molecular oxygen over H-terminated pentacoordinated Ti site in Beta zeolite at 813 K (polymorph C).

Note: The energy barrier of PO formation over H-terminated pentacoordinated Ti site in Beta zeolite at 813 K (polymorph C) (**TS1**, E_a= 1.05 eV; **TS3**, E_a= 0.82 eV) is slightly lower than that of acrolein formation (**TS1**, E_a= 1.05 eV; **TS5'**, E_a= 0.90 eV).

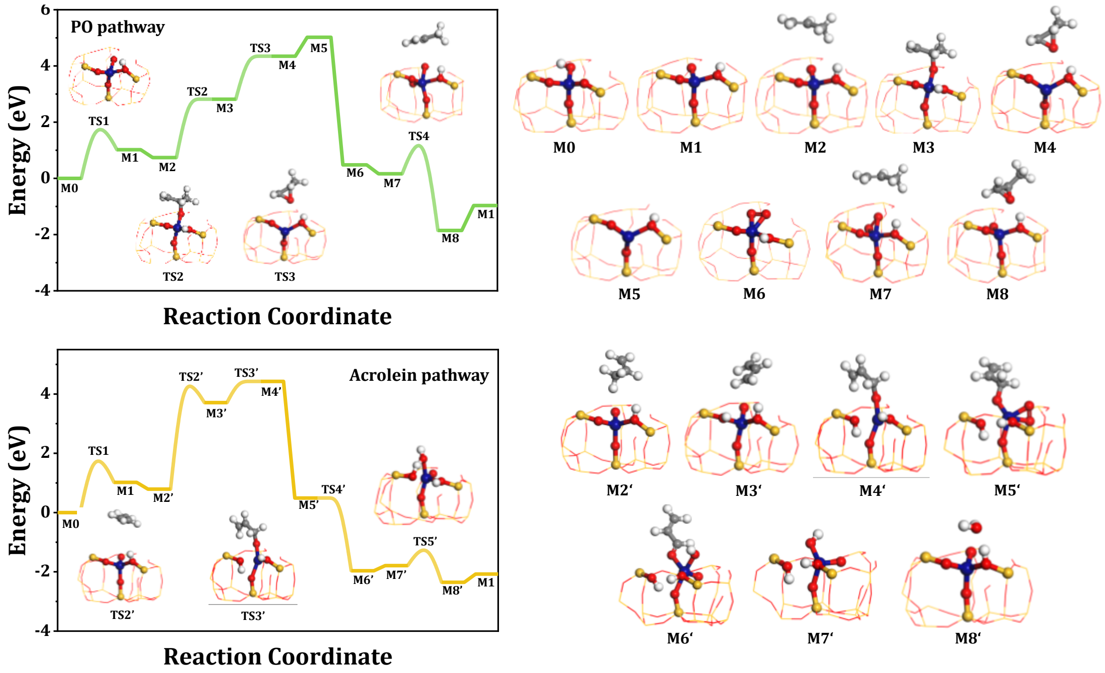

Supplementary Figure 60| Proposed reaction pathways, optimized intermediate structures, transition state structures and the corresponding energy profiles in the formation of PO and acrolein over H-terminated tetracoordinated open Ti site in Beta zeolite at 0 K (polymorph C).

Note: The energy barrier of PO formation over H-terminated tetracoordinated Ti site in Beta zeolite at 0 K (polymorph C) (**TS2**, E_a= 2.08 eV) is lower than that of acrolein formation (**TS2'**, E_a= 3.52 eV).

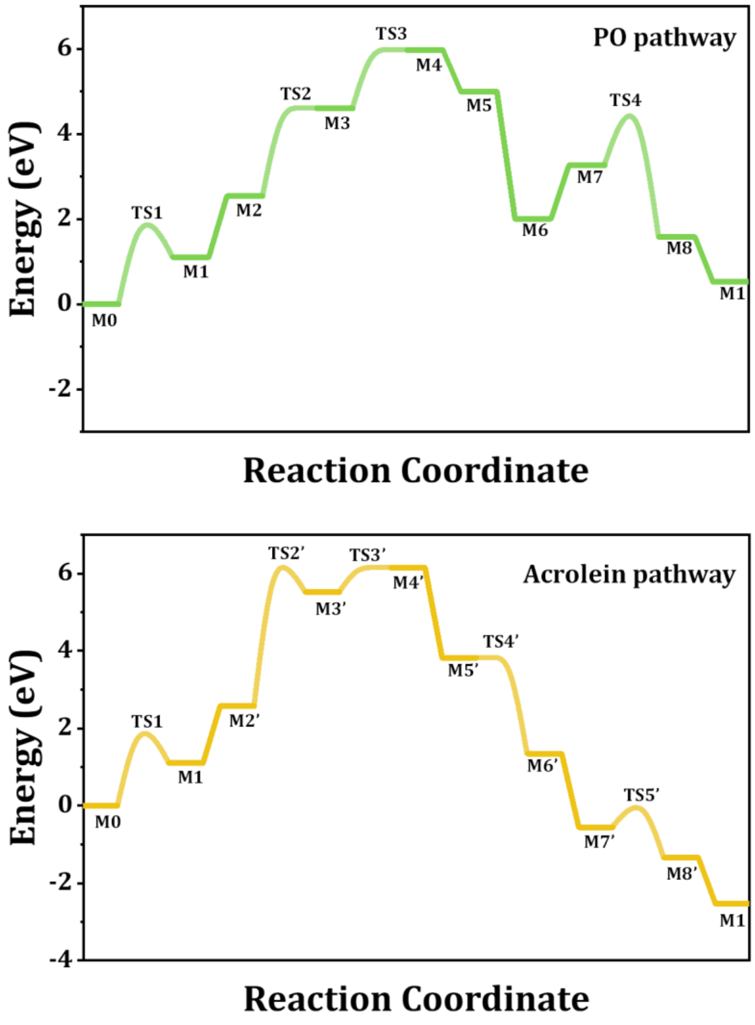

Supplementary Figure 61| Calculated energy profile of propylene oxidation with molecular oxygen over H-terminated tetracoordinated open Ti site in Beta zeolite at 813 K (polymorph C).

Note: The energy barrier of PO formation over H-terminated tetracoordinated Ti site in Beta zeolite at 813 K (polymorph C) (**TS2**, E_a= 2.06 eV) is lower than that of acrolein formation (**TS2'**, E_a= 3.63 eV).

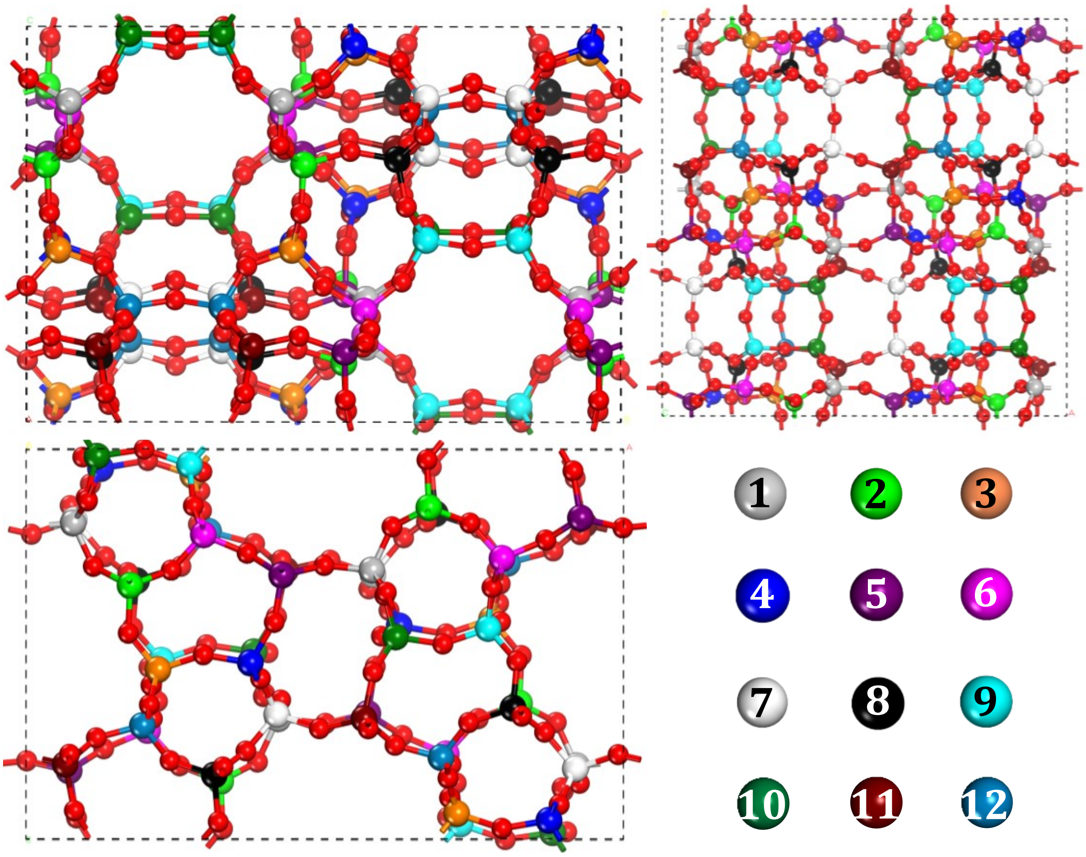

Supplementary Figure 62| Various crystallographic T sites in MFI framework.

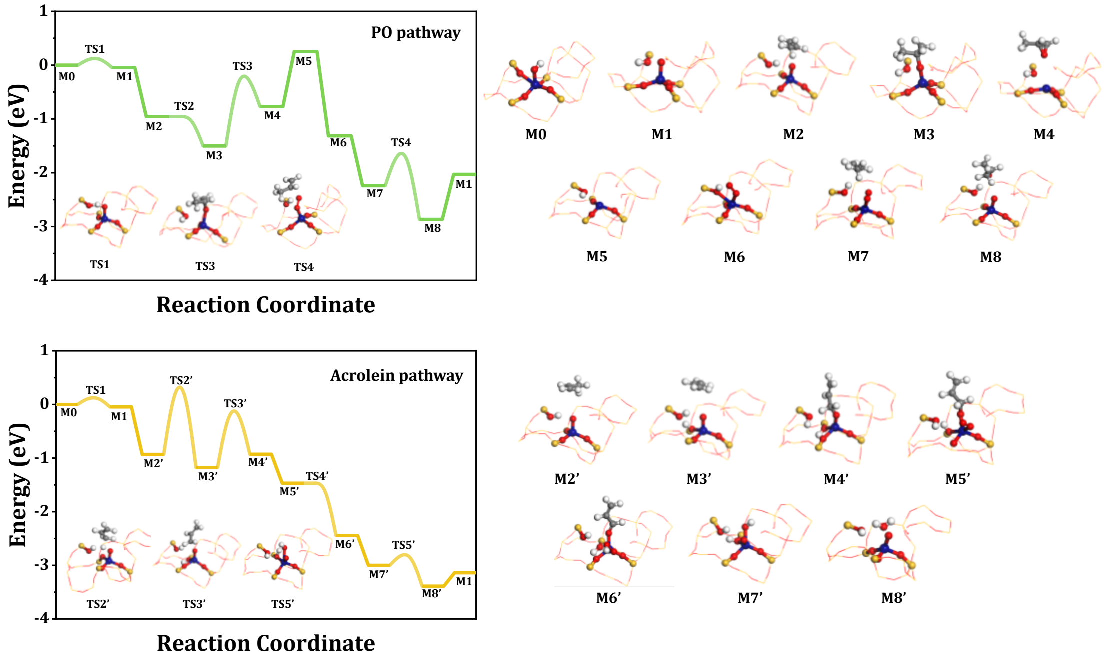

Supplementary Figure 63| Proposed reaction pathways, optimized intermediate structures, transition state structures and the corresponding energy profiles in the formation of PO and acrolein over H-terminated pentacoordinated Ti site in MFI zeolite at 0 K.

Note: The energy barrier of PO formation over H-terminated pentacoordinated Ti site in MFI zeolite at 0 K (**TS3**, E_a= 1.33 eV) is slightly higher than that of acrolein formation (**TS2'**, E_a= 1.31 eV).

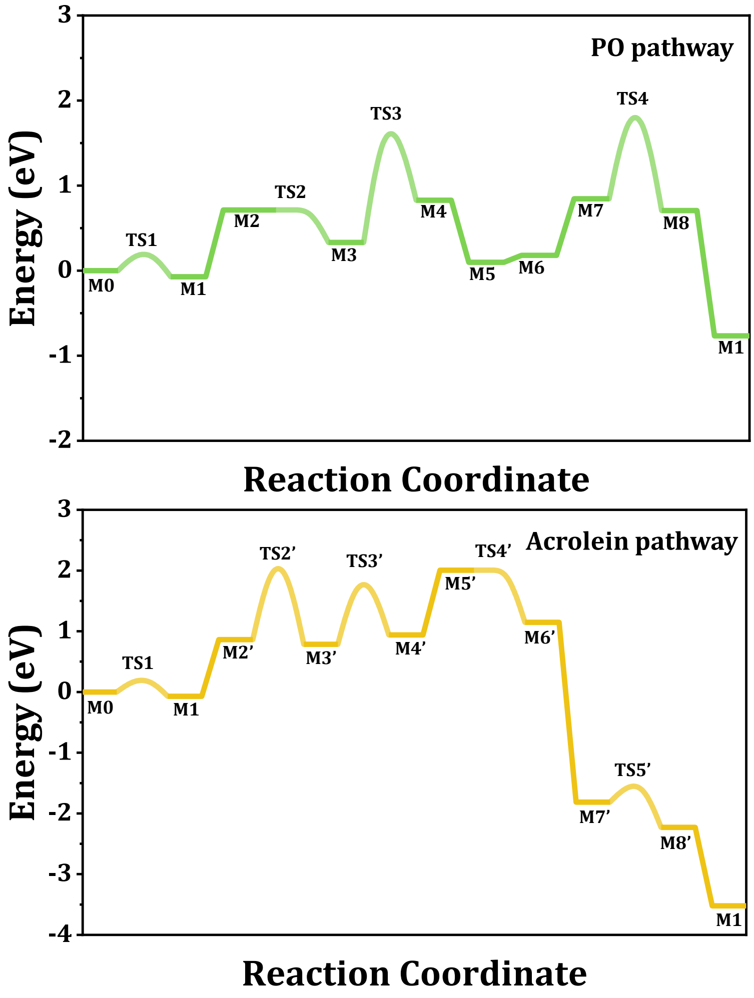

Supplementary Figure 64| Calculated energy profile of propylene oxidation with molecular oxygen over H-terminated pentacoordinated Ti site in MFI zeolite at 813 K.

Note: The energy barrier of PO formation over H-terminated pentacoordinated Ti site in MFI zeolite at 813 K (**TS3**, E_a= 1.32 eV) is higher than that of acrolein formation (**TS2'**, E_a= 1.22 eV).

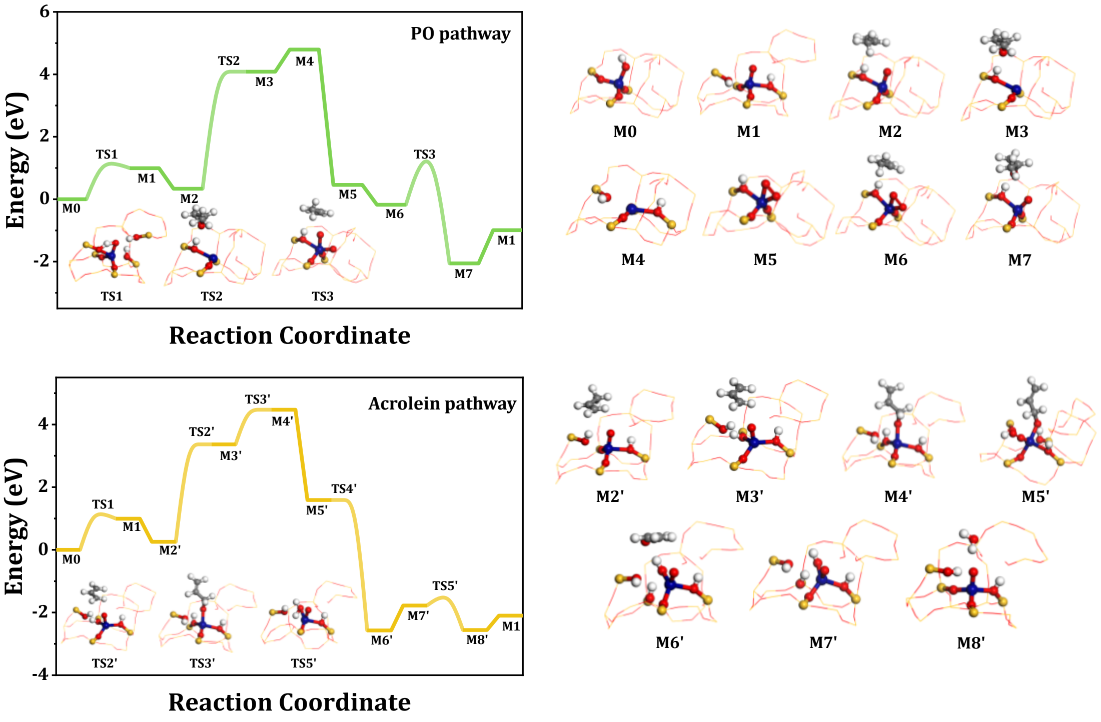

Supplementary Figure 65| Proposed reaction pathways, optimized intermediate structures, transition state structures and the corresponding energy profiles in the formation of PO and acrolein over H-terminated tetracoordinated open Ti site in MFI zeolite at 0 K.

Note: The energy barrier of PO formation over H-terminated tetracoordinated Ti site in MFI zeolite at 0 K (**TS2**, E_a= 3.75 eV) is higher than that of acrolein formation (**TS2'**, E_a= 3.11 eV).

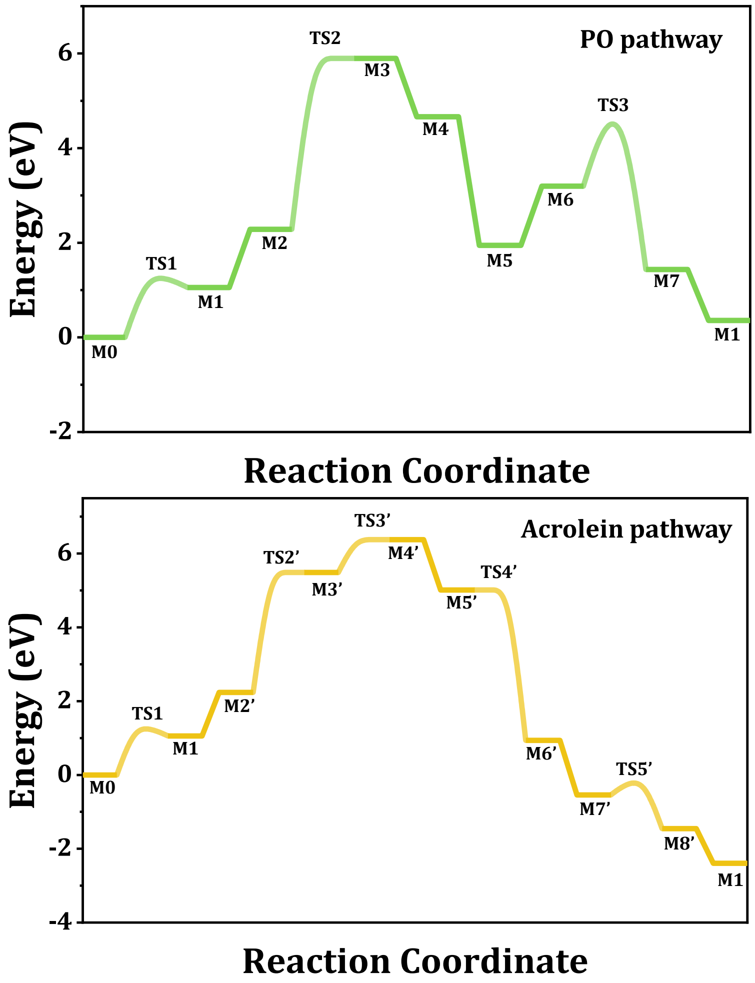

Supplementary Figure 66| Calculated energy profile of propylene oxidation with molecular oxygen over H-terminated tetracoordinated open Ti site in MFI zeolite at 813 K.

Note: The energy barrier of PO formation over H-terminated tetracoordinated Ti site in MFI zeolite at 813 K (**TS2**, E_a= 3.61 eV) is higher than that of acrolein formation (**TS2'**, E_a= 3.25 eV).

Supplementary Table 1| Texture properties of Ti-Beta zeolite samples.

Sample	Si/Ti [a]	Si/Al [a]	Micropore volume (cm^3/g zeolite) [b]	Micropore area (m^2/g zeolite) [b]	External surface area (m^2/g zeolite)
Ti-Beta-1%	54	>1800	0.178	448	154
Ti-Beta-2%	40	>1800	0.188	461	161
Ti-Beta-3%	27	>1800	0.179	456	158
Ti-Beta-4%	20	>1800	0.183	453	158
Ti-Beta-3%-spent	28	>1800	0.184	459	143

[a]: Determined by ICP-OES; [b]: Calculated *via* the de Boer t-plot method.

Supplementary Table 2| Comparison between EO and PO production *via* aerobic epoxidation[4,60]

Reaction	$2\ C_2H_4 + O_2 \rightarrow 2\ C_2H_4O$	$2\ C_3H_6 + O_2 \rightarrow 2\ C_3H_6O$
Atomic efficiency [a]	91.2/ 100 %	85.3/ 100 %
Reaction type	Gas-phase	Gas-phase
Reaction temperature	503-543 K	793-833 K
Catalyst	Modified Ag/Al_2O_3	Ti-Beta
Additive	$C_2H_4Cl_2$	None
Substrate conversion	C_2H_4: 12-15 %	C_3H_6: 11-25 %
Olefin recirculation	Yes	Yes
Product selectivity	EO: ~90 %	PO: 85-90 %

[a]: practical atomic efficiency / theoretical atomic efficiency

Supplementary Table 3| Comparison of various catalysts for propylene aerobic epoxidation.

Catalyst	Reaction conditions	Conv. (%)	PO Select. (%)	PO formation (mmol/g/h)	Ref.
Cu_2O (110)	8%C_3H_6-4%O_2-88%N_2<br>50 mL/min, 0.2 g catalyst, 473 K	0.2	50	0.06	*8*
Cl-RD-Cu_2O	13.3%C_3H_6-6.7%O_2-80%He<br>50 mL/min, 0.2 g catalyst, 473 K	1	63	0.5	*10*
NaCl-Ag/$CaCO_3$	16.7%C_3H_6-33.3%O_2-50%He<br>30 mL/min, 0.5 g catalyst, 533 K	3.7	45	1.24	*11*
Au/TS-1-KOH	10%C_3H_6-10%O_2-2%H_2O-78%Ar<br>20 mL/min, 0.3 g catalyst, 473 K	0.88	52	0.19	*13*
Co@Y	4%C_3H_6-3.2%O_2-92.8%He<br>60 mL/min, 0.2 g catalyst, 773 K	24.6	57	4.72	*17*
Na^+-CuO_x/SiO_2	16.7%C_3H_6-16.7%O_2-66.6%He<br>30 mL/min, 0.5 g catalyst, 623 K	5	14	1.38	*61*
Cubic Cu_2O	8%C_3H_6-4%O_2-88%Ar<br>50 mL/min, 0.2g catalyst, 383 K	0.06	82	0.0246	*62*
Cu/SiO_2	5%C_3H_6-5%O_2-90%He<br>50 mL/min, 0.1 g catalyst, 498 K	0.25	53	0.014	*63*
CuO-CeO_2	5%C_3H_6-5%CO-10%O_2-80%He<br>50 mL/min, 0.5 g catalyst, 353 K	0.68	95	0.078	*64*
K^+-CuO_x<br>/SBA-15	50%C_3H_6-32%O_2-18%He<br>60 mL/min, 0.2 g catalyst, 623 K	2.1	21	2.1	*65*
KAc-Cu/SiO_2	5.88%C_3H_6-5.88%O_2-88.24%N_2<br>100 mL/min, 0.2 mL catalyst, 523 K	1.3	61	0.24	*66*
MoO_3-Bi_2SiO_5<br>/SiO_2	8%C_3H_6-16%O_2-76%N_2<br>25 mL/min, 0.1g catalyst, 673 K	15.8	61	4.7	*67*
NaCl-$VCe_{0.5}Cu_{0.5}$	4%C_3H_6-3.2%O_2-92.8%He<br>60 mL/min, 0.2 g catalyst, 523 K	0.26	33	0.165	*68*
Ti-Beta-3%	5%C_3H_6-5%O_2-90%He<br>60 mL/min, 0.1 g catalyst, 833 K	25.0	90	14.0	This work
Ti-Beta-4%	5%C_3H_6-5%O_2-90%He<br>60 mL/min, 0.1 g catalyst, 833 K	33.1	78	15.6	This work

Supplementary Table 4| T-O distances (*r*, Å), cell volumes (V*c*, Å^3) and relative energies (ΔE, eV) for the nine distinct T sites in Ti-Beta and H-Beta (polymorph A).

Site	r_1 (Ti-O)	r_2 (Ti-O)	r_3 (Ti-O)	V*c*	ΔE
T1	1.800	1.812	1.816	4198.80	0.12
T2	1.794	1.810	1.813	4198.80	0.12
T3	1.799	1.811	1.814	4198.68	0.12
T4	1.795	1.813	1.814	4198.71	0.15
T5	1.804	1.805	1.809	4198.80	0.03
T6	1.806	1.811	1.811	4198.70	0.00
T7	1.801	1.806	1.811	4198.76	0.19
T8	1.809	1.809	1.812	4198.48	0.15
T9	1.807	1.807	1.807	4198.51	0.22
Site	r_1 (Al-O)	r_2 (Al-O)	r_3 (Al-O)	V*c*	ΔE
T1	1.741	1.743	1.750	4198.80	0.14
T2	1.731	1.743	1.748	4198.84	0.10
T3	1.733	1.750	1.751	4198.68	0.09
T4	1.734	1.747	1.749	4198.71	0.09
T5	1.731	1.744	1.750	4198.80	0.03
T6	1.735	1.743	1.750	4198.70	0.00
T7	1.736	1.743	1.747	4198.76	0.16
T8	1.741	1.741	1.743	4198.48	0.13
T9	1.734	1.746	1.746	4198.51	0.19

Supplementary Table 5| All symbols of intermediates in the energy profile in Figure 3 and Supplementary Figs. 39-47.

Product	Symbol	Intermediate
C_3H_6O	M0	$OH_{\_Ti}*$
	M1	$[O_{\_Ti}+H_{\_O}]*$
	M2	$[O_{\_Ti}+CH_2=CHCH_{3\_g}+H_{\_O}]*$
	M3	$[OCH(CH_2)CH_{3\_Ti}+H_{\_O}]*$
	M4	$[C_3H_6O_{\_g}+H_{\_O}]*$
	M5	$H_{\_O}*$
	M6	$[O_{2\_Ti}+H_{\_O}]*$
	M7	$[O_{2\_Ti}+CH_2=CHCH_{3\_g}+H_{\_O}]*$
	M8	$[OOCH(CH_2)CH_{3\_Ti}+H_{\_O}]*$
	M9	$[O_{\_Ti}+C_3H_6O_{\_g}+H_{\_O}]*$
$OCHCH=CH_2$ (C_3H_4O)	M2'	$[O_{\_Ti}+CH_2=CHCH_{3\_g}+H_{\_O}]*$
	M3'	$[O_{\_Ti}+CH_2=CHCH_{2\_g}+2H_{\_O}]*$
	M4'	$[OCH_2CH=CH_{2\_Ti}+2H_{\_O}]*$
	M5'	$[OCH_2CH=CH_{2\_Ti}+O_{2\_Ti}+2H_{\_O}]*$
	M6'	$[OCHCH=CH_{2\_Ti}+O_2H_{\_Ti}+2H_{\_O}]*$
	M7'	$[O_2H_{\_Ti}+2H_{\_O}]*$
	M8'	$[O_{\_Ti}+H_2O_{\_Ti}+H_{\_O}]*$

Note: Underline plus Ti (_Ti) means adsorbates at the Ti site; Underline plus g (_g) means that adsorbates adsorb above the Ti site. Underline plus O (_O) means adsorbates at the O site adjacent to the Ti site; Square brackets plus asterisk ([]*) represents the co-adsorption state of adsorbates.

Supplementary Table 6| Elementary reaction steps involved in the energy profile in Figure 3 and Supplementary Figs. 46-47.

Reaction step	TS	Simplification
$OH_{\_Ti}*\rightarrow[O_{\_Ti}+H_{\_O}]*$	TS1	M0 → M1
$[O_{\_Ti}+H_{\_O}]*+CH_2{=}CHCH_3(g)\rightarrow[O_{\_Ti}+CH_2{=}CHCH_{3\_g}+H_{\_O}]*$	–	M1 → M2
$[O_{\_Ti}+CH_2{=}CHCH_{3\_g}+H_{\_O}]*\rightarrow[OCH(CH_2)CH_{3\_Ti}+H_{\_O}]*$	TS2	M2 → M3
$[OCH(CH_2)CH_{3\_Ti}+H_{\_O}]*\rightarrow[C_3H_6O_{\_g}+H_{\_O}]*$	TS3	M3 → M4
$[C_3H_6O_{\_g}+H_{\_O}]*\rightarrow H_{\_O}*+C_3H_6O(g)$	–	M4 → M5
$H_{\_O}*+O_2(g)\rightarrow[O_{2\_Ti}+H_{\_O}]*$	–	M5 → M6
$[O_{2\_Ti}+H_{\_O}]*+CH_2{=}CHCH_3(g)\rightarrow[O_{2\_Ti}+CH_2{=}CHCH_{3\_g}+H_{\_O}]*$	–	M6 → M7
$[O_{2\_Ti}+CH_2{=}CHCH_{3\_g}+H_{\_O}]*\rightarrow[OOCH(CH_2)CH_{3\_Ti}+H_{\_O}]*$	TS4	M7 → M8
$[OOCH(CH_2)CH_{3\_Ti}+H_{\_O}]*\rightarrow[O_{\_Ti}+C_3H_6O_{\_g}+H_{\_O}]*$	TS5	M8 → M9
$[O_{\_Ti}+C_3H_6O_{\_g}+H_{\_O}]*\rightarrow[O_{\_Ti}+H_{\_O}]*+C_3H_6O(g)$	–	M9 → M1
$[O_{\_Ti}+H_{\_O}]*\rightarrow OH_{\_Ti}*$	$TS1^{*}$	M1 → M0
$[O_{\_Ti}+H_{\_O}]*+CH_2{=}CHCH_3(g)\rightarrow[O_{\_Ti}+CH_2{=}CHCH_{3\_g}+H_{\_O}]*$	–	M1 → M2'
$[O_{\_Ti}+CH_2{=}CHCH_{3\_g}+H_{\_O}]*\rightarrow[O_{\_Ti}+CH_2{=}CHCH_{2\_g}+2H_{\_O}]*$	TS2'	M2' → M3'
$[O_{\_Ti}+CH_2{=}CHCH_{2\_g}+2H_{\_O}]*\rightarrow[OCH_2CH{=}CH_{2\_Ti}+2H_{\_O}]*$	TS3'	M3' → M4'
$[OCH_2CH{=}CH_{2\_Ti}+2H_{\_O}]*+O_2(g)\rightarrow[OCH_2CH{=}CH_{2\_Ti}+O_{2\_Ti}+2H_{\_O}]*$	–	M4' → M5'
$[OCH_2CH{=}CH_{2\_Ti}+O_{2\_Ti}+2H_{\_O}]*\rightarrow[OCHCH{=}CH_{2\_Ti}+O_2H_{\_Ti}+2H_{\_O}]*$	TS4'	M5' → M6'
$[OCHCH{=}CH_{2\_Ti}+O_2H_{\_Ti}+2H_{\_O}]*\rightarrow[O_2H_{\_Ti}+2H_{\_O}]*+OCHCH{=}CH_2(g)$	–	M6' → M7'
$[O_2H_{\_Ti}+2H_{\_O}]*\rightarrow[O_{\_Ti}+H_2O_{\_Ti}+H_{\_O}]*$	TS5'	M7' → M8'
$[O_{\_Ti}+H_2O_{\_Ti}+H_{\_O}]*\rightarrow[O_{\_Ti}+H_{\_O}]*+H_2O(g)$	–	M8' → M1

Note: g in parenthesis, (g), indicates that gaseous molecules are outside the cage of the zeolite.

Supplementary Table 7| Elementary reaction steps involved in the energy profile in Supplementary Figs. 49-50.

Reaction step	TS	Simplification
$\mathrm{OH_{\_Ti}*\rightarrow[O_{\_Ti}+H_{\_O}]*}$	TS1	M0 → M1
$\mathrm{[O_{\_Ti}+H_{\_O}]*+CH_2{=}CHCH_3(g)\rightarrow[O_{\_Ti}+CH_2{=}CHCH_{3\_g}+H_{\_O}]*}$	–	M1 → M2
$\mathrm{[O_{\_Ti}+CH_2{=}CHCH_{3\_g}+H_{\_O}]*\rightarrow[OCH(CH_2)CH_{3\_Ti}+H_{\_O}]*}$	TS2	M2 → M3
$\mathrm{[OCH(CH_2)CH_{3\_Ti}+H_{\_O}]*\rightarrow[C_3H_6O_{\_Ti}+H_{\_O}]*}$	TS3	M3 → M4
$\mathrm{[C_3H_6O_{\_Ti}+H_{\_O}]*\rightarrow H_{\_O}*+C_3H_6O(g)}$	–	M4 → M5
$\mathrm{H_{\_O}*+O_2(g)\rightarrow[O_{2\_Ti}+H_{\_O}]*}$	–	M5 → M6
$\mathrm{[O_{2\_Ti}+H_{\_O}]*+CH_2{=}CHCH_3(g)\rightarrow[O_{2\_Ti}+CH_2{=}CHCH_{3\_g}+H_{\_O}]*}$	–	M6 → M7
$\mathrm{[O_{2\_Ti}+CH_2{=}CHCH_{3\_g}+H_{\_O}]*\rightarrow[O_{\_Ti}+C_3H_6O_{\_g}+H_{\_O}]*}$	TS4	M7 → M8
$\mathrm{[O_{\_Ti}+C_3H_6O_{\_g}+H_{\_O}]*\rightarrow[O_{\_Ti}+H_{\_O}]*+C_3H_6O(g)}$	–	M8 → M1
$\mathrm{[O_{\_Ti}+H_{\_O}]*\rightarrow OH_{\_Ti}*}$	TS1*	M1 → M0
$\mathrm{[O_{\_Ti}+H_{\_O}]*+CH_2{=}CHCH_3(g)\rightarrow[O_{\_Ti}+CH_2{=}CHCH_{3\_g}+H_{\_O}]*}$	–	M1 → M2'
$\mathrm{[O_{\_Ti}+CH_2{=}CHCH_{3\_g}+H_{\_O}]*\rightarrow[O_{\_Ti}+CH_2{=}CHCH_{2\_g}+2H_{\_O}]*}$	TS2'	M2' → M3'
$\mathrm{[O_{\_Ti}+CH_2{=}CHCH_{2\_g}+2H_{\_O}]*\rightarrow[OCH_2CH{=}CH_{2\_Ti}+2H_{\_O}]*}$	TS3'	M3' → M4'
$\mathrm{[OCH_2CH{=}CH_{2\_Ti}+2H_{\_O}]*+O_2(g)\rightarrow[OCH_2CH{=}CH_{2\_Ti}+O_{2\_Ti}+2H_{\_O}]*}$	–	M4' → M5'
$\mathrm{[OCH_2CH{=}CH_{2\_Ti}+O_{2\_Ti}+2H_{\_O}]*\rightarrow[OCHCH{=}CH_{2\_g}+O_2H_{\_Ti}+2H_{\_O}]*}$	TS4'	M5' → M6'
$\mathrm{[OCHCH{=}CH_{2\_g}+O_2H_{\_Ti}+2H_{\_O}]*\rightarrow[O_2H_{\_Ti}+2H_{\_O}]*+OCHCH{=}CH_2(g)}$	–	M6' → M7'
$\mathrm{[O_2H_{\_Ti}+2H_{\_O}]*\rightarrow[O_{\_Ti}+H_2O_{\_Ti}+H_{\_O}]*}$	TS5'	M7' → M8'
$\mathrm{[O_{\_Ti}+H_2O_{\_Ti}+H_{\_O}]*\rightarrow[O_{\_Ti}+H_{\_O}]*+H_2O(g)}$	–	M8' → M1

Supplementary Table 8| Elementary reaction steps involved in the energy profile in Supplementary Figs. 53-54.

Reaction step	TS	Simplification
$OH_{\_Ti}* \rightarrow [O_{\_Ti}+H_{\_O}]*$	TS1	M0 → M1
$[O_{\_Ti}+H_{\_O}]*+CH_2{=}CHCH_3(g) \rightarrow [O_{\_Ti}+CH_2{=}CHCH_{3\_g}+H_{\_O}]*$	–	M1 → M2
$[O_{\_Ti}+CH_2{=}CHCH_{3\_g}+H_{\_O}]* \rightarrow [OCH(CH_2)CH_{3\_Ti}+H_{\_O}]*$	TS2	M2 → M3
$[OCH(CH_2)CH_{3\_Ti}+H_{\_O}]* \rightarrow [C_3H_6O_{\_g}+H_{\_O}]*$	TS3	M3 → M4
$[C_3H_6O_{\_g}+H_{\_O}]* \rightarrow H_{\_O}*+C_3H_6O(g)$	–	M4 → M5
$H_{\_O}*+O_2(g) \rightarrow [O_{2\_Ti}+H_{\_O}]*$	–	M5 → M6
$[O_{2\_Ti}+H_{\_O}]*+CH_2{=}CHCH_3(g) \rightarrow [O_{2\_Ti}+CH_2{=}CHCH_{3\_g}+H_{\_O}]*$	–	M6 → M7
$[O_{2\_Ti}+CH_2{=}CHCH_{3\_g}+H_{\_O}]* \rightarrow [OOCH(CH_2)CH_{3\_Ti}+H_{\_O}]*$	TS4	M7 → M8
$[OOCH(CH_2)CH_{3\_Ti}+H_{\_O}]* \rightarrow [O_{\_Ti}+C_3H_6O_{\_g}+H_{\_O}]*$	TS5	M8 → M9
$[O_{\_Ti}+C_3H_6O_{\_g}+H_{\_O}]* \rightarrow [O_{\_Ti}+H_{\_O}]*+C_3H_6O(g)$	–	M9 → M1
$[O_{\_Ti}+H_{\_O}]* \rightarrow OH_{\_Ti}*$	TS1*	M1 → M0
$[O_{\_Ti}+H_{\_O}]*+CH_2{=}CHCH_3(g) \rightarrow [O_{\_Ti}+CH_2{=}CHCH_{3\_g}+H_{\_O}]*$	–	M1 → M2'
$[O_{\_Ti}+CH_2{=}CHCH_{3\_g}+H_{\_O}]* \rightarrow [O_{\_Ti}+CH_2{=}CHCH_{2\_g}+2H_{\_O}]*$	TS2'	M2' → M3'
$[O_{\_Ti}+CH_2{=}CHCH_{2\_g}+2H_{\_O}]* \rightarrow [OCH_2CH{=}CH_{2\_Ti}+2H_{\_O}]*$	TS3'	M3' → M4'
$[OCH_2CH{=}CH_{2\_Ti}+2H_{\_O}]* \rightarrow [OCH_2CH{=}CH_{2\_g}+2H_{\_O}]*$	–	M4' → M5'
$[OCH_2CH{=}CH_{2\_g}+2H_{\_O}]*+O_2(g) \rightarrow [O_{2\_Ti}+OCH_2CH{=}CH_{2\_g}+2H_{\_O}]*$	–	M5' → M6'
$[O_{2\_Ti}+OCH_2CH{=}CH_{2\_g}+2H_{\_O}]* \rightarrow [O_{\_Ti}+OH_{\_Ti}+OCHCH{=}CH_{2\_g}+2H_{\_O}]*$	TS4'	M6' → M7'
$[O_{\_Ti}+OH_{\_Ti}+OCHCH{=}CH_{2\_g}+2H_{\_O}]* \rightarrow [O_{\_Ti}+OH_{\_Ti}+2H_{\_O}]*+OCHCH{=}CH_2(g)$	–	M7' → M8'
$[O_{\_Ti}+OH_{\_Ti}+2H_{\_O}]* \rightarrow [O_{\_Ti}+H_2O_{\_g}+H_{\_O}]*$	TS5'	M8' → M9'
$[O_{\_Ti}+H_2O_{\_g}+H_{\_O}]* \rightarrow [O_{\_Ti}+H_{\_O}]*+H_2O(g)$	–	M9' → M1

Supplementary Table 9| Elementary reaction steps involved in the energy profile in Supplementary Figs. 55-56.

Reaction step	TS	Simplification
$OH_{\_Ti}* \rightarrow [O_{\_Ti}+H_{\_O}]*$	TS1	M0 → M1
$[O_{\_Ti}+H_{\_O}]*+CH_2{=}CHCH_3(g) \rightarrow [O_{\_Ti}+CH_2{=}CHCH_{3\_g}+H_{\_O}]*$	–	M1 → M2
$[O_{\_Ti}+CH_2{=}CHCH_{3\_g}+H_{\_O}]* \rightarrow [C_3H_6O_{\_g}+H_{\_O}]*$	TS2	M2 → M3
$[C_3H_6O_{\_g}+H_{\_O}]* \rightarrow H_{\_O}*+C_3H_6O(g)$	–	M3 → M4
$H_{\_O}*+O_2(g) \rightarrow [O_{2\_Ti}+H_{\_O}]*$	–	M4 → M5
$[O_{2\_Ti}+H_{\_O}]*+CH_2{=}CHCH_3(g) \rightarrow [O_{2\_Ti}+CH_2{=}CHCH_{3\_g}+H_{\_O}]*$	–	M5 → M6
$[O_{2\_Ti}+CH_2{=}CHCH_{3\_g}+H_{\_O}]* \rightarrow [O_{\_Ti}+C_3H_6O_{\_g}+H_{\_O}]*$	TS3	M6 → M7
$[O_{\_Ti}+C_3H_6O_{\_g}+H_{\_O}]* \rightarrow [O_{\_Ti}+H_{\_O}]*+C_3H_6O(g)$	–	M7 → M1
$[O_{\_Ti}+H_{\_O}]* \rightarrow OH_{\_Ti}*$	TS1*	M1 → M0
$[O_{\_Ti}+H_{\_O}]*+CH_2{=}CHCH_3(g) \rightarrow [O_{\_Ti}+CH_2{=}CHCH_{3\_g}+H_{\_O}]*$	–	M1 → M2'
$[O_{\_Ti}+CH_2{=}CHCH_{3\_g}+H_{\_O}]* \rightarrow [O_{\_Ti}+CH_2{=}CHCH_{2\_g}+2H_{\_O}]*$	TS2'	M2' → M3'
$[O_{\_Ti}+CH_2{=}CHCH_{2\_g}+2H_{\_O}]* \rightarrow [OCH_2CH{=}CH_{2\_Ti}+2H_{\_O}]*$	TS3'	M3' → M4'
$[OCH_2CH{=}CH_{2\_Ti}+2H_{\_O}]* \rightarrow [OCH_2CH{=}CH_{2\_g}+2H_{\_O}]*$	–	M4' → M5'
$[OCH_2CH{=}CH_{2\_g}+2H_{\_O}]*+O_2(g) \rightarrow [O_{2\_Ti}+OCH_2CH{=}CH_{2\_g}+2H_{\_O}]*$	–	M5' → M6'
$[O_{2\_Ti}+OCH_2CH{=}CH_{2\_g}+2H_{\_O}]* \rightarrow [O_{\_Ti}+OH_{\_Ti}+OCHCH{=}CH_{2\_g}+2H_{\_O}]*$	TS4'	M6' → M7'
$[O_{\_Ti}+OH_{\_Ti}+OCHCH{=}CH_{2\_g}+2H_{\_O}]* \rightarrow [O_{\_Ti}+OH_{\_Ti}+2H_{\_O}]*+OCHCH{=}CH_2(g)$	–	M7' → M8'
$[O_{\_Ti}+OH_{\_Ti}+2H_{\_O}]* \rightarrow [O_{\_Ti}+H_2O_{\_g}+H_{\_O}]*$	TS5'	M8' → M9'
$[O_{\_Ti}+H_2O_{\_g}+H_{\_O}]* \rightarrow [O_{\_Ti}+H_{\_O}]*+H_2O(g)$	–	M9' → M1

Supplementary Table 10| Elementary reaction steps involved in the energy profile in Supplementary Figs. 58-59.

Reaction step	TS	Simplification
$OH_{\_Ti}* \rightarrow [O_{\_Ti}+H_{\_O}]*$	TS1	M0 → M1
$[O_{\_Ti}+H_{\_O}]*+CH_2=CHCH_3(g) \rightarrow [O_{\_Ti}+CH_2=CHCH_{3\_g}+H_{\_O}]*$	–	M1 → M2
$[O_{\_Ti}+CH_2=CHCH_{3\_g}+H_{\_O}]* \rightarrow [OCH(CH_2)CH_{3\_Ti}+H_{\_O}]*$	TS2	M2 → M3
$[OCH(CH_2)CH_{3\_Ti}+H_{\_O}]* \rightarrow [C_3H_6O_{\_g}+H_{\_O}]*$	TS3	M3 → M4
$[C_3H_6O_{\_g}+H_{\_O}]* \rightarrow H_{\_O}*+C_3H_6O(g)$	–	M4 → M5
$H_{\_O}*+O_2(g) \rightarrow [O_{2\_Ti}+H_{\_O}]*$	–	M5 → M6
$[O_{2\_Ti}+H_{\_O}]*+CH_2=CHCH_3(g) \rightarrow [O_{2\_Ti}+CH_2=CHCH_{3\_g}+H_{\_O}]*$	–	M6 → M7
$[O_{2\_Ti}+CH_2=CHCH_{3\_g}+H_{\_O}]* \rightarrow [OOCH(CH_2)CH_{3\_Ti}+H_{\_O}]*$	TS4	M7 → M8
$[OOCH(CH_2)CH_{3\_Ti}+H_{\_O}]* \rightarrow [O_{\_Ti}+C_3H_6O_{\_g}+H_{\_O}]*$	TS5	M8 → M9
$[O_{\_Ti}+C_3H_6O_{\_g}+H_{\_O}]* \rightarrow [O_{\_Ti}+H_{\_O}]*+C_3H_6O(g)$	–	M9 → M1
$[O_{\_Ti}+H_{\_O}]* \rightarrow OH_{\_Ti}*$	TS1*	M1 → M0
$[O_{\_Ti}+H_{\_O}]*+CH_2=CHCH_3(g) \rightarrow [O_{\_Ti}+CH_2=CHCH_{3\_g}+H_{\_O}]*$	–	M1 → M2'
$[O_{\_Ti}+CH_2=CHCH_{3\_g}+H_{\_O}]* \rightarrow [O_{\_Ti}+CH_2=CHCH_{2\_g}+2H_{\_O}]*$	TS2'	M2' → M3'
$[O_{\_Ti}+CH_2=CHCH_{2\_g}+2H_{\_O}]* \rightarrow [OCH_2CH=CH_{2\_Ti}+2H_{\_O}]*$	TS3'	M3' → M4'
$[OCH_2CH=CH_{2\_Ti}+2H_{\_O}]*+O_2(g) \rightarrow [OCH_2CH=CH_{2\_Ti}+O_{2\_Ti}+2H_{\_O}]*$	–	M4' → M5'
$[OCH_2CH=CH_{2\_Ti}+O_{2\_Ti}+2H_{\_O}]* \rightarrow [OCHCH=CH_{2\_Ti}+O_2H_{\_Ti}+2H_{\_O}]*$	TS4'	M5' → M6'
$[OCHCH=CH_{2\_Ti}+O_2H_{\_Ti}+2H_{\_O}]* \rightarrow [O_2H_{\_Ti}+2H_{\_O}]*+OCHCH=CH_2(g)$	–	M6' → M7'
$[O_2H_{\_Ti}+2H_{\_O}]* \rightarrow [O_{\_Ti}+H_2O_{\_Ti}+H_{\_O}]*$	TS5'	M7' → M8'
$[O_{\_Ti}+H_2O_{\_Ti}+H_{\_O}]* \rightarrow [O_{\_Ti}+H_{\_O}]*+H_2O(g)$	–	M8' → M1

Supplementary Table 11| Elementary reaction steps involved in the energy profile in Supplementary Figs. 60-61.

Reaction step	TS	Simplification
$OH_{\_Ti}{*}\rightarrow[O_{\_Ti}+H_{\_O}]{*}$	TS1	M0 → M1
$[O_{\_Ti}+H_{\_O}]{*}+CH_2=CHCH_3(g)\rightarrow[O_{\_Ti}+CH_2=CHCH_{3\_g}+H_{\_O}]{*}$	–	M1 → M2
$[O_{\_Ti}+CH_2=CHCH_{3\_g}+H_{\_O}]{*}\rightarrow[OCH(CH_2)CH_{3\_Ti}+H_{\_O}]{*}$	TS2	M2 → M3
$[OCH(CH_2)CH_{3\_Ti}+H_{\_O}]{*}\rightarrow[C_3H_6O_{\_g}+H_{\_O}]{*}$	TS3	M3 → M4
$[C_3H_6O_{\_g}+H_{\_O}]{*}\rightarrow H_{\_O}{*}+C_3H_6O(g)$	–	M4 → M5
$H_{\_O}{*}+O_2(g)\rightarrow[O_{2\_Ti}+H_{\_O}]{*}$	–	M5 → M6
$[O_{2\_Ti}+H_{\_O}]{*}+CH_2=CHCH_3(g)\rightarrow[O_{2\_Ti}+CH_2=CHCH_{3\_g}+H_{\_O}]{*}$	–	M6 → M7
$[O_{2\_Ti}+CH_2=CHCH_{3\_g}+H_{\_O}]{*}\rightarrow[O_{\_Ti}+C_3H_6O_{\_g}+H_{\_O}]{*}$	TS4	M7 → M8
$[O_{\_Ti}+C_3H_6O_{\_g}+H_{\_O}]{*}\rightarrow[O_{\_Ti}+H_{\_O}]{*}+C_3H_6O(g)$	–	M8 → M1
$[O_{\_Ti}+H_{\_O}]{*}\rightarrow OH_{\_Ti}{*}$	TS1*	M1 → M0
$[O_{\_Ti}+H_{\_O}]{*}+CH_2=CHCH_3(g)\rightarrow[O_{\_Ti}+CH_2=CHCH_{3\_g}+H_{\_O}]{*}$	–	M1 → M2'
$[O_{\_Ti}+CH_2=CHCH_{3\_g}+H_{\_O}]{*}\rightarrow[O_{\_Ti}+CH_2=CHCH_{2\_g}+2H_{\_O}]{*}$	TS2'	M2' → M3'
$[O_{\_Ti}+CH_2=CHCH_{2\_g}+2H_{\_O}]{*}\rightarrow[OCH_2CH=CH_{2\_Ti}+2H_{\_O}]{*}$	TS3'	M3' → M4'
$[OCH_2CH=CH_{2\_Ti}+2H_{\_O}]{*}+O_2(g)\rightarrow[OCH_2CH=CH_{2\_Ti}+O_{2\_Ti}+2H_{\_O}]{*}$	–	M4' → M5'
$[OCH_2CH=CH_{2\_Ti}+O_{2\_Ti}+2H_{\_O}]{*}\rightarrow[OCHCH=CH_{2\_Ti}+O_2H_{\_Ti}+2H_{\_O}]{*}$	TS4'	M5' → M6'
$[OCHCH=CH_{2\_Ti}+O_2H_{\_Ti}+2H_{\_O}]{*}\rightarrow[O_2H_{\_Ti}+2H_{\_O}]{*}+OCHCH=CH_2(g)$	–	M6' → M7'
$[O_2H_{\_Ti}+2H_{\_O}]{*}\rightarrow[O_{\_Ti}+H_2O_{\_g}+H_{\_O}]{*}$	TS5'	M7' → M8'
$[O_{\_Ti}+H_2O_{\_g}+H_{\_O}]{*}\rightarrow[O_{\_Ti}+H_{\_O}]{*}+H_2O(g)$	–	M8' → M1

Supplementary Table 12| Elementary reaction steps involved in the energy profile in Supplementary Figs. 63-64.

Reaction step	TS	Simplification
$OH_{\_Ti}* \rightarrow [O_{\_Ti}+H_{\_O}]*$	TS1	M0 → M1
$[O_{\_Ti}+H_{\_O}]*+CH_2{=}CHCH_3(g) \rightarrow [O_{\_Ti}+CH_2{=}CHCH_{3\_g}+H_{\_O}]*$	–	M1 → M2
$[O_{\_Ti}+CH_2{=}CHCH_{3\_g}+H_{\_O}]* \rightarrow [OCH(CH_2)CH_{3\_Ti}+H_{\_O}]*$	TS2	M2 → M3
$[OCH(CH_2)CH_{3\_Ti}+H_{\_O}]* \rightarrow [C_3H_6O_{\_g}+H_{\_O}]*$	TS3	M3 → M4
$[C_3H_6O_{\_g}+H_{\_O}]* \rightarrow H_{\_O}*+C_3H_6O(g)$	–	M4 → M5
$H_{\_O}*+O_2(g) \rightarrow [O_{2\_Ti}+H_{\_O}]*$	–	M5 → M6
$[O_{2\_Ti}+H_{\_O}]*+CH_2{=}CHCH_3(g) \rightarrow [O_{2\_Ti}+CH_2{=}CHCH_{3\_g}+H_{\_O}]*$	–	M6 → M7
$[O_{2\_Ti}+CH_2{=}CHCH_{3\_g}+H_{\_O}]* \rightarrow [O_{\_Ti}+C_3H_6O_{\_g}+H_{\_O}]*$	TS4	M7 → M8
$[O_{\_Ti}+C_3H_6O_{\_g}+H_{\_O}]* \rightarrow [O_{\_Ti}+H_{\_O}]*+C_3H_6O(g)$	–	M8 → M1
$[O_{\_Ti}+H_{\_O}]* \rightarrow OH_{\_Ti}*$	TS1*	M1 → M0
$[O_{\_Ti}+H_{\_O}]*+CH_2{=}CHCH_3(g) \rightarrow [O_{\_Ti}+CH_2{=}CHCH_{3\_g}+H_{\_O}]*$	–	M1 → M2'
$[O_{\_Ti}+CH_2{=}CHCH_{3\_g}+H_{\_O}]* \rightarrow [O_{\_Ti}+CH_2{=}CHCH_{2\_g}+2H_{\_O}]*$	TS2'	M2' → M3'
$[O_{\_Ti}+CH_2{=}CHCH_{2\_g}+2H_{\_O}]* \rightarrow [OCH_2CH{=}CH_{2\_Ti}+2H_{\_O}]*$	TS3'	M3' → M4'
$[OCH_2CH{=}CH_{2\_Ti}+2H_{\_O}]*+O_2(g) \rightarrow [OCH_2CH{=}CH_{2\_Ti}+O_{2\_Ti}+2H_{\_O}]*$	–	M4' → M5'
$[OCH_2CH{=}CH_{2\_Ti}+O_{2\_Ti}+2H_{\_O}]* \rightarrow [OCHCH{=}CH_{2\_Ti}+O_2H_{\_Ti}+2H_{\_O}]*$	TS4'	M5' → M6'
$[OCHCH{=}CH_{2\_Ti}+O_2H_{\_Ti}+2H_{\_O}]* \rightarrow [O_2H_{\_Ti}+2H_{\_O}]*+OCHCH{=}CH_2(g)$	–	M6' → M7'
$[O_2H_{\_Ti}+2H_{\_O}]* \rightarrow [O_{\_Ti}+H_2O_{\_g}+H_{\_O}]*$	TS5'	M7' → M8'
$[O_{\_Ti}+H_2O_{\_g}+H_{\_O}]* \rightarrow [O_{\_Ti}+H_{\_O}]*+H_2O(g)$	–	M8' → M1

Supplementary Table 13| Elementary reaction steps involved in the energy profile in Supplementary Figs. 65-66.

Reaction step	TS	Simplification
$OH_{\_Ti}*\rightarrow[O_{\_Ti}+H_{\_O}]*$	TS1	M0 → M1
$[O_{\_Ti}+H_{\_O}]*+CH_2=CHCH_3(g)\rightarrow[O_{\_Ti}+CH_2=CHCH_{3\_g}+H_{\_O}]*$	–	M1 → M2
$[O_{\_Ti}+CH_2=CHCH_{3\_g}+H_{\_O}]*\rightarrow[C_3H_6O_{\_g}+H_{\_O}]*$	TS2	M2 → M3
$[C_3H_6O_{\_g}+H_{\_O}]*\rightarrow H_{\_O}*+C_3H_6O(g)$	–	M3 → M4
$H_{\_O}*+O_2(g)\rightarrow[O_{2\_Ti}+H_{\_O}]*$	–	M4 → M5
$[O_{2\_Ti}+H_{\_O}]*+CH_2=CHCH_3(g)\rightarrow[O_{2\_Ti}+CH_2=CHCH_{3\_g}+H_{\_O}]*$	–	M5 → M6
$[O_{2\_Ti}+CH_2=CHCH_{3\_g}+H_{\_O}]*\rightarrow[O_{\_Ti}+C_3H_6O_{\_g}+H_{\_O}]*$	TS3	M6 → M7
$[O_{\_Ti}+C_3H_6O_{\_g}+H_{\_O}]*\rightarrow[O_{\_Ti}+H_{\_O}]*+C_3H_6O(g)$	–	M7 → M1
$[O_{\_Ti}+H_{\_O}]*\rightarrow OH_{\_Ti}*$	$TS1^{*}$	M1 → M0
$[O_{\_Ti}+H_{\_O}]*+CH_2=CHCH_3(g)\rightarrow[O_{\_Ti}+CH_2=CHCH_{3\_g}+H_{\_O}]*$	–	M1 → M2'
$[O_{\_Ti}+CH_2=CHCH_{3\_g}+H_{\_O}]*\rightarrow[O_{\_Ti}+CH_2=CHCH_{2\_g}+2H_{\_O}]*$	TS2'	M2' → M3'
$[O_{\_Ti}+CH_2=CHCH_{2\_g}+2H_{\_O}]*\rightarrow[OCH_2CH=CH_{2\_Ti}+2H_{\_O}]*$	TS3'	M3' → M4'
$[OCH_2CH=CH_{2\_Ti}+2H_{\_O}]*+O_2(g)\rightarrow[OCH_2CH=CH_{2\_Ti}+O_{2\_Ti}+2H_{\_O}]*$	–	M4' → M5'
$[OCH_2CH=CH_{2\_Ti}+O_{2\_Ti}+2H_{\_O}]*\rightarrow[OCHCH=CH_{2\_g}+O_2H_{\_Ti}+2H_{\_O}]*$	TS4'	M5' → M6'
$[OCHCH=CH_{2\_g}+O_2H_{\_Ti}+2H_{\_O}]*\rightarrow[O_2H_{\_Ti}+2H_{\_O}]*+OCHCH=CH_2(g)$	–	M6' → M7'
$[O_2H_{\_Ti}+2H_{\_O}]*\rightarrow[O_{\_Ti}+H_2O_{\_g}+H_{\_O}]*$	TS5'	M7' → M8'
$[O_{\_Ti}+H_2O_{\_g}+H_{\_O}]*\rightarrow[O_{\_Ti}+H_{\_O}]*+H_2O(g)$	–	M8' → M1

References

1. Nexant Inc. Market Analytics: Propylene Oxide- 2018 (2018).
2. H. Baer *et al.*, Propylene Oxide. in *Ullmann's Encyclopedia of Industrial Chemistry* 1-29 Wiley-VCH (2012).
3. T. A. Nijhuis *et al.*, The Production of Propene Oxide: Catalytic Processes and Recent Developments. *Ind. Eng. Chem. Res.* **45**, 3447-3459 (2006).
4. T. Pu, et al. Overview of Selective Oxidation of Ethylene to Ethylene Oxide by Ag Catalysts. *ACS Catal.* **9**, 10727-10750 (2019).
5. S. J. Khatib, S. T. Oyama, Direct Oxidation of Propylene to Propylene Oxide with Molecular Oxygen: A Review. *Catal. Rev. Sci. Eng.* **57**, 306-344 (2015).
6. J. Teržan *et al.*, Propylene Epoxidation Using Molecular Oxygen over Copper-and Silver-Based Catalysts: A Review. *ACS Catal.* **10**, 13415-13436 (2020).
7. A. Marimuthu, J. Zhang, S. Linic, Tuning Selectivity in Propylene Epoxidation by Plasmon Mediated Photo-Switching of Cu Oxidation State. *Science* **340**, 1590-1593 (2013).
8. Q. Hua, *et al.*, Crystal-Plane-Controlled Selectivity of Cu_2O Catalysts in Propylene Oxidation with Molecular Oxygen. *Angew. Chem. Int. Ed.* **53**, 4856-4861 (2014).
9. X. Yang *et al.*, Direct Epoxidation of Propylene over Stabilized Cu^+ Surface Sites on Titanium-Modified Cu_2O. *Angew. Chem. Int. Ed.* **54**, 11946-11951 (2015).
10. C. Zhan, *et al.*, Critical Roles of Doping Cl on Cu_2O Nanocrystals for Direct Epoxidation of Propylene by Molecular Oxygen. *J. Am. Chem. Soc.* **142**, 14134-14141 (2020).
11. J. Lu *et al.*, Direct Propylene Epoxidation over Modified Ag/$CaCO_3$ Catalysts. *Appl. Catal. A Gen.* **302**, 283-295 (2006).
12. Y. Lei *et al.*, Increased Silver Activity for Direct Propylene Epoxidation *via* Subnanometer Size Effects. *Science* **328**, 224-228 (2010).
13. M. D. Hughes *et al.*, Tunable Gold Catalysts for Selective Hydrocarbon Oxidation under Mild Conditions. *Nature* **437**, 1132-1135 (2005).
14. J. Huang *et al.*, Propene Epoxidation with Dioxygen Catalyzed by Gold Clusters. *Angew. Chem. Int. Ed.* **48**, 7862-7866 (2009).
15. A. S. K. Hashmi, G. J. Hutchings, Gold Catalysis. *Angew. Chem. Int. Ed.* **45**, 7896-7936 (2006).
16. C. L. Bracey *et al.*, Understanding the Effect of Thermal Treatments on the Structure of CuAu/SiO_2 Catalysts and Their Performance in Propene Oxidation. *Catal. Sci. Technol.* **1**, 76-85 (2011).
17. W. Li *et al.*, Direct Propylene Epoxidation with Molecular Oxygen over Cobalt-Containing Zeolites. *J. Am. Chem. Soc.* **144**, 4260-4268 (2022).
18. M. McCoy, New Routes to Propylene Oxide. *Chem. Eng. News* **79**, 19-20 (2001).
19. B. Tang *et al.*, A Procedure for the Preparation of Ti-Beta Zeolites for Catalytic Epoxidation with Hydrogen Peroxide. *Green Chem.* **16**, 2281-2291 (2014).
20. B. S. Lane, K. Burgess, Metal-catalyzed Epoxidations of Alkenes with Hydrogen Peroxide. *Chem. Rev.* **103**, 2457-2473 (2003).
21. C. P. Gordon *et al.*, Efficient Epoxidation over Dinuclear Sites in Titanium Silicalite-1. *Nature* **586**, 708-713 (2020).
22. V. Russo *et al.*, Chemical and Technical Aspects of Propene Oxide Production via Hydrogen Peroxide (HPPO Process). *Ind. Eng. Chem. Res.* **52**, 1168-1178 (2013).

23. A. Corma, H García, Lewis Acids as Catalysts in Oxidation Reactions: From Homogeneous to Heterogeneous Systems. *Chem. Rev.* **102**, 3837-3892 (2002).
24. J. R. H. Ross, The Kinetics and Mechanisms of Catalytic Reactions. In *Contemporary Catalysis: Fundamentals and Current Applications* 161-186 Elsevier (2019).
25. W. Chen *et al.*, Mesokinetics as a Tool Bridging the Microscopic-to-Macroscopic Transition to Rationalize Catalyst Design. *Acc. Chem. Res.* **22**, 3230-3241 (2022).
26. W. Chen *et al.*, Taming Electrons in Pt/C Catalysts to Boost the Mesokinetics of Hydrogen Production. *Engineering* **14**, 124-133 (2022).
27. Q. Guo *et al.*, A Thorough Investigation of the Active Titanium Species in TS-1 Zeolite by *In Situ* UV Resonance Raman Spectroscopy. *Chem. Eur. J.* **18**, 13854-13860 (2012).
28. M. Signorile *et al.*, Titanium Defective Sites in TS-1: Structural Insights by Combining Spectroscopy and Simulation. *Angew. Chem. Int. Ed.* **59**, 18145-18150 (2020).
29. G. Yang, E. A. Pidko, E. J. M. Hensen, Structure, Stability, and Lewis Acidity of Mono and Double Ti, Zr, and Sn Framework Substitutions in BEA Zeolites: A Periodic Density Functional Theory Study. *J. Phys. Chem. C.* **117**, 3976-3986 (2013).
30. Y. P. Li, M. Head-Gordon, A. T. Bell, Analysis of the Reaction Mechanism and Catalytic Activity of Metal-substituted Beta Zeolite for the Isomerization of Glucose to Fructose. *ACS Catal.* **4**, 1537-1545 (2014).
31. D. H. Wells, W. N. Delgass, K. T. Thomson, Evidence of Defect-Promoted Reactivity for Epoxidation of Propylene in Titanosilicate (TS-1) Catalysts: A DFT Study. *J. Am. Chem. Soc.* **126**, 2956-2962 (2004).
32. W. O. Parker, R. Millini, Ti Coordination in Titanium Silicalite-1. *J. Am. Chem. Soc.* **128**, 1450-1451 (2006).
33. T. Maschmeyer *et al.*, Heterogeneous Catalysts Obtained by Grafting Metallocene Complexes onto Mesoporous Silica. *Nature* **378**, 159-162 (1995).
34. M. G. Clerici, U. Romano, Process for the Epoxidation of Olefinic Compounds and Catalysts Used therein., U.S. Patent 4,937,216 (1990).
35. Blasco, T. *et al.* Unseeded Synthesis of Al-free Ti-β Zeolite in Fluoride Medium: A Hydrophobic Selective Oxidation Catalyst. *Chem. Commun.* **3**, 2367-2368 (1996).
36. G. C. Sinke, D. L. Hildenbrand, Heat of Formation of Propylene Oxide. *J. Chem. Eng. Data* **7**, 74-74 (1962).
37. M. Zhang *et al.*, How to Measure the Reaction Performance of Heterogeneous Catalytic Reactions Reliably. *Joule* **3**, 2876-2883 (2019).
38. H. Zeng *et al.*, Orthogonal-array Dynamic Molecular Sieving of Propylene/Propane Mixtures. *Nature* **595**, 542-548 (2021).
39. Z. X. Yan *et al.*, Shock-induced Thermal Behavior of Aluminum Nanoparticles in Propylene Oxide. *J. Appl. Phys.* **101**, 024905 (2007).
40. E. L. First *et al.*, Computational Characterization of Zeolite Porous Networks: An Automated Approach. *Phys. Chem. Chem. Phys.* **13**, 17339 (2011).
41. R. J. Madon, M. Boudart, Experimental Criterion for the Absence of Artifacts in the Measurement of Rates of Heterogeneous Catalytic Reactions. *Ind. Eng. Chem. Fundam.* **21**, 438-447 (1982).
42. G. Kresse, J. Furthmüller, Efficiency of Ab-initio Total Energy Calculations for Metals and Semiconductors Using a Plane-Wave Basis Set, *Comp. Mater. Sci.* **6**, 15-50 (1996).

43. G. Kresse, J. Furthmüller, Efficient Iterative Schemes for Ab Initio Total-Energy Calculations Using a Plane-Wave Basis Set, *Phys. Rev. B* **54**, 11169-11186 (1996).
44. J.P. Perdew, K. Burke, M. Ernzerhof, Generalized Gradient Approximation Made Simple, *Phys. Rev. Lett.* **77**, 3865-3868 (1996).
45. P.E. Blöchl, Projector Augmented-Wave Method, *Phys. Rev.* B **50**, 17953-17979 (1994).
46. J. Wellendorff, K.T. Lundgaard, A. Møgelhøj, V. Petzold, D.D. Landis, J.K. Nørskov, T. Bligaard, K.W. Jacobsen, Density Functionals for Surface Science: Exchange-correlation Model Development with Bayesian Error Estimation, *Phys. Rev.* B **85**, 235149-235171 (2012).
47. L. Sun, Y. Wang, C. Wang, Z. Xie, N. Guan, L. Li, Water-involved Methane-Selective Catalytic Oxidation by Dioxygen over Copper Zeolites, *Chem* **7**, 1557-1568 (2021).
48. G. Henkelman, H. Jónsson, Improved Tangent Estimate in the Nudged Elastic Band Method for Finding Minimum Energy Paths and Saddle Points, *J. Chem. Phys.* **113**, 9978-9985 (2000).
49. G. Henkelman, B.P. Uberuaga, H. Jónsson, A Climbing Image Nudged Elastic Band Method for Finding Saddle Points and Minimum Energy Paths, *J. Chem. Phys.* **113**, 9901-9904 (2000).
50. M. Martínez-Iñesta, I. Peral, T. Proffen, R. Lobo, A Pair Distribution Function Analysis of Zeolite Beta. *Micropor. Mesopor. Mater.* **77**, 55-66 (2005).
51. A. Corma *et al.,* Pure Polymorph C of Zeolite Beta Synthesized by Using Framework Isomorphous Substitution as a Structure-Directing Mechanism. *Angew. Chem. Int. Ed.* **40**, 2277-2280 (2001).
52. C. Lamberti *et al.,* Ti Location in the MFI Framework of Ti-Silicalite-1: A Neutron Powder Diffraction Study. *J. Am. Chem. Soc.* **123**, 2204-2212 (2001).
53. S. Dzwigaj *et al.*, Role of Silanol Groups in the Incorporation of V in β Zeolite. *J. Mol. Catal. A Chem.* **155**, 169-182 (2000).
54. N. Topsoe, Characterization of the Nature of Surface Sites on Vanadia-Titania Catalysts by FTIR. *J. Catal.* **128**, 499-511 (1991).
55. M. S. Morey *et al.*, Hydrothermal and postsynthesis Surface Modification of Cubic, MCM-48, and ultralarge Pore SBA-15 Mesoporous Silica with Titanium. *Chem. Mater.* **12**, 898-911 (2000).
56. W. Lin, H. Frei, Photochemical and FT-IR Probing of the Active Site of Hydrogen Peroxide in Ti Silicalite Sieve. *J. Am. Chem. Soc.* **124**, 9292-9298 (2002).
57. Y. Jiang *et al.*, Solid-state Nuclear Magnetic Resonance Investigations of the Nature, Property, and Activity of Acid Sites on Solid Catalysts. *Solid State Nucl. Magn. Reson.* **39**, 116-141 (2011).
58. V. M. Mastikhin, A. V. Nosov, ^{1}H NMR Studies of the OH Groups of Anatase. *React. Kinet. Catal. Lett.* **46**, 123-130 (1992).
59. M. Crocker *et al.*, ^{1}H NMR spectroscopy of titania. Chemical Shift Assignments for Hydroxy Groups in Crystalline and Amorphous Forms of TiO_2. *J. Chem. Soc. Faraday Trans.* **92**, 2791-2798 (1996).
60. S. Haukka, E. L. Lakomaa, A. Root, An IR and NMR Study of the Chemisorption of Titanium Tetrachloride on Silica. *J. Phys. Chem.* **97**, 5085-5094 (1993).
61. L. Peng, H. Huo, Y. Liu, C.P. Grey, ^{17}O Magic Angle Spinning NMR Studies of Brønsted Acid

Sites in Zeolites HY and HZSM-5. *J. Am. Chem. Soc.* **129**, 335-346 (2007).

62. H. van Milligen, B. VanderWilp, G. J. Wells, Enhancements in Ethylene Oxide/Ethylene Glycol Manufacturing. *Shell Catalysts & Technologies* 2021.

63. J. Teržan *et al.*, Effect of Na, Cs and Ca on Propylene Epoxidation Selectivity over CuO_x/SiO_2 Catalysts Studied by Catalytic Tests, in-situ XAS and DFT. *Appl. Surf. Sci.* **528**, 146854 (2020).

64. W. Xiong *et al.*, Fine Cubic Cu_2O Nanocrystals as Highly Selective Catalyst for Propylene Epoxidation with Molecular Oxygen. *Nat. Commun.* **12**, 5921-5928 (2021).

65. O. P. H. Vaughan *et al.*, Copper as a Selective Catalyst for the Epoxidation of Propene. *J. Catal.* **236**, 401-404 (2005).

66. T. Baidya *et al.*, Low-temperature Propylene Epoxidation Activity of CuO–CeO_2 Catalyst with CO + O_2: Role of Metal–support Interaction on the Reducibility and Catalytic Property of CuO_x Species. *J. Phys. Chem. C* **124**, 14131-14146 (2020).

67. H. Chu *et al.*, Copper-catalyzed Propylene Epoxidation by Molecular Oxygen: Superior Catalytic Performances of Halogen-free K^+-Modified CuO_x/SBA-15. *J. Catal.* **241**, 225-228 (2006).

68. W. Su *et al.*, A Molecular Insight into Propylene Epoxidation on Cu/SiO_2 Catalysts using O_2 as Oxidant. *J. Catal.* **268**, 165-174 (2009).

69. Y. Lei *et al.*, Enhanced Catalytic Performance in the Gas-Phase Epoxidation of Propylene over Ti-Modified MoO_3 –Bi_2SiO_5 /SiO_2 Catalysts. *J. Catal.* **321**, 100-112 (2015).

70. J. Lu *et al.*, Epoxidation of Propylene on NaCl-Modified $VCe_{1-x}Cu_x$ Oxide Catalysts with Direct Molecular Oxygen as the Oxidant. *J. Catal.* **211**, 552-555 (2002).
